# Genetic variation in hippocampal microRNA expression differences in C57BL/6 J X DBA/2 J (BXD) recombinant inbred mouse strains

**DOI:** 10.1186/1471-2164-13-476

**Published:** 2012-09-13

**Authors:** Michael J Parsons, Christina Grimm, Jose L Paya-Cano, Cathy Fernandes, Lin Liu, Vivek M Philip, Elissa J Chesler, Wilfried Nietfeld, Hans Lehrach, Leonard C Schalkwyk

**Affiliations:** 1 Mammalian Genetics Unit, Medical Research Council – Harwell, Oxfordshire, United Kingdom; 2 Max Planck Institute for Molecular Genetics (MPIMG), Department of Vertebrate Genomics, Berlin, Germany; 3 Social, Genetic, and Developmental Psychiatry Centre, Institute of Psychiatry, London, King's College London, UK; 4 Graduate School of Genome Science and Technology, University of Tennessee, Knoxville, TN, USA; 5 The Jackson Laboratory, Bar Harbor, Maine, USA

## Abstract

**Background:**

miRNAs are short single-stranded non-coding RNAs involved in post-transcriptional gene regulation that play a major role in normal biological functions and diseases. Little is currently known about how expression of miRNAs is regulated. We surveyed variation in miRNA abundance in the hippocampus of mouse inbred strains, allowing us to take a genetic approach to the study of miRNA regulation, which is novel for miRNAs. The BXD recombinant inbred panel is a very well characterized genetic reference panel which allows quantitative trait locus (QTL) analysis of miRNA abundance and detection of correlates in a large store of brain and behavioural phenotypes.

**Results:**

We found five suggestive *trans* QTLs for the regulation of miRNAs investigated. Further analysis of these QTLs revealed two genes, *Tnik* and *Phf17,* under the *miR-212* regulatory QTLs, whose expression levels were significantly correlated with miR-212 expression. We found that miR-212 expression is correlated with cocaine-related behaviour, consistent with a reported role for this miRNA in the control of cocaine consumption. miR-31 is correlated with anxiety and alcohol related behaviours. KEGG pathway analysis of each miRNA’s expression correlates revealed enrichment of pathways including MAP kinase, cancer, long-term potentiation, axonal guidance and WNT signalling.

**Conclusions:**

The BXD reference panel allowed us to establish genetic regulation and characterize biological function of specific miRNAs. QTL analysis enabled detection of genetic loci that regulate the expression of these miRNAs. eQTLs that regulate miRNA abundance are a new mechanism by which genetic variation influences brain and behaviour. Analysis of one of these QTLs revealed a gene, *Tnik,* which may regulate the expression of a miRNA, a molecular pathway and a behavioural phenotype. Evidence of genetic covariation of miR-212 abundance and cocaine related behaviours is strongly supported by previous functional studies, demonstrating the value of this approach for discovery of new functional roles and downstream processes regulated by miRNA.

## Background

Micro-ribonucleic acids (miRNAs) are short single-stranded non-coding RNAs that are involved in the post-transcriptional regulation of genes. MiRNAs are processed from primary transcripts into smaller stem-loop precursor RNAs in the nucleus by the enzyme DROSHA and then further processed by DICER in the cytoplasm into the mature miRNA [1]. Only the mature form of miRNAs cause post-transcriptional gene silencing by imperfect base pairing, as part of a larger molecular complex, with its target sites which in turn can lead to mRNA cleavage or translational repression [2,3]. A single miRNA can have predicted target sites on hundreds of different mRNAs and thus have wide ranging effects on mRNA expression [1]. Roughly one-third of all genes have at least one predicted miRNA target site [4]. However, the small size of the miRNAs, combined with the complex nature of the miRNA and miRNA target interactions, makes prediction of miRNA target genes difficult [5].

MiRNAs play a major role in a wide range of functions including development, immune processes, apoptosis and synapse formation [3,6-11]. MiRNAs have also been shown to play an important role in numerous diseases including cancer, heart disease and mental disorders [12-14]. The associations between miRNAs and these phenotypes and diseases are likely due to the effects of miRNAs on the expression of specific groups of mRNAs via post-transcriptional regulation. Thus, genetic variation that effects miRNA expression will likely affect these phenotypes. Numerous studies have used genetic correlation of gene expression with a variety of phenotypes to discover gene functions in biological processes including brain and behaviour [15,16]. By exploring patterns in the networks of gene expression genetically correlated with individual miRNAs we may be able to determine each miRNA’s underlying function.

While many miRNAs are widely expressed, the expression of some miRNAs has a spatial specificity [17,18]. This is the case in adult brain, where miRNAs expression is often exclusive to particular regions [19] or where there are families of miRNAs that are preferentially expressed in specific brain areas, such as the hippocampus or frontal cortex [17]. This suggests that individual miRNAs may only play a role in defined anatomical areas.

Though much is known about the general function and regulation of miRNAs, much work is still needed to determine the specific functioning of individual miRNAs. A high-throughput functional genomics strategy to investigate the functions of individual miRNAs makes use of the genetic variation across mouse inbred strains and allows one to see how this variation affects miRNA expression. To date, three studies have used this approach to establish variation in miRNA expression across inbred mouse strains, one in the hippocampus [20] and two in the liver [21,22]. We previously identified several differentially expressed hippocampal miRNAs in the C57BL/6 J and DBA/2 J strains [20]. Finding the genetic source of this variation and identifying the functional associates requires a genetic reference population that has been broadly characterized for brain and behavioural function.

In this study we further characterized differentially expressed hippocampal miRNAs between the inbred mouse strains C57BL/6 J and DBA/2 J [20] in a C57BL/6 J x DBA/2 J recombinant inbred panel (BXD). This is the first study to use BXD RI mice to investigate the functioning of specific miRNAs in the brain. The wide array of cumulative data available for these strains allowed us to evaluate associations with a wide range of phenotypes including mRNA expression, neurological and behavioural phenotypes, and perform QTL mapping. This approach allowed us to identify upstream genetic loci that influence functioning of these specific miRNA and downstream gene expression and behavioural correlates which may be influenced by the same loci.

## Results

### Genetic variation in miRNA expression in the BXD RI lines

The means (± standard deviations) of hippocampal miRNA expression for all the BXD RI strains investigated are listed in Table 1. We replicated the reported expression differences across the C57BL/6 J vs. DBA/2 J strains for the five miRNAs we previously found to be differentially expressed [20]. The *η*^2^ values for miRNA gene expression by strain are 0.21, 0.33, 0.28, 0.27 and 0.37 for miR-15b, miR-31, miR-34c, miR-212 and miR-301a, respectively. 

**Table 1:** Strain means and standard deviation for miRNA expression

**strain**	***miR-15b***			***miR-31***			***miR-34c***			***miR-212***			***miR-301a***		
	mean		st dev	mean		st dev	mean		st dev	mean		st dev	mean		st dev
BXD 01TY	0.063	±	0.03	0.099	±	0.04	0.064	±	0.03	0.190	±	0.12	0.219	±	0.09
BXD 05TY	0.058	±	0.01	0.070	±	0.02	0.034	±	0.01	0.117	±	0.04	0.164	±	0.04
BXD 06TY	0.053	±	0.02	0.072	±	0.01	0.053	±	0.03	0.112	±	0.02	0.183	±	0.03
BXD 08TY	0.056	±	0.01	0.090	±	0.02	0.041	±	0.01	0.137	±	0.01	0.176	±	0.02
BXD 09TY	0.050	±	0.02	0.132	±	0.10	0.076	±	0.04	0.141	±	0.05	0.171	±	0.04
BXD 11TY	0.053	±	0.02	0.074	±	0.01	0.045	±	0.02	0.148	±	0.10	0.180	±	0.06
BXD 12TY	0.048	±	0.00	0.066	±	0.01	0.040	±	0.01	0.114	±	0.02	0.192	±	0.04
BXD 16TY	0.044	±	0.02	0.099	±	0.01	0.054	±	0.01	0.115	±	0.03	0.210	±	0.04
BXD 18TY	0.042	±	0.01	0.083	±	0.01	0.044	±	0.01	0.142	±	0.03	0.173	±	0.02
BXD 19TY	0.053	±	0.01	0.060	±	0.01	0.061	±	0.03	0.139	±	0.03	0.194	±	0.02
BXD 21TY	0.055	±	0.01	0.072	±	0.02	0.060	±	0.02	0.152	±	0.04	0.188	±	0.04
BXD 22TY	0.055	±	0.02	0.094	±	0.03	0.055	±	0.01	0.097	±	0.02	0.186	±	0.02
BXD 23TY	0.051	±	0.01	0.127	±	0.07	0.061	±	0.01	0.114	±	0.01	0.167	±	0.02
BXD 24TY	0.045	±	0.01	0.071	±	0.03	0.045	±	0.03	0.091	±	0.03	0.157	±	0.03
BXD 27TY	0.049	±	0.01	0.064	±	0.01	0.063	±	0.02	0.128	±	0.02	0.159	±	0.02
BXD 28TY	0.045	±	0.02	0.071	±	0.02	0.044	±	0.01	0.124	±	0.06	0.137	±	0.08
BXD 30TY	0.036	±	0.00	0.057	±	0.02	0.033	±	0.02	0.104	±	0.03	0.132	±	0.03
BXD 31TY	0.055	±	0.01	0.074	±	0.02	0.040	±	0.01	0.123	±	0.02	0.187	±	0.02
BXD 32TY	0.038	±	0.02	0.071	±	0.01	0.049	±	0.03	0.118	±	0.05	0.158	±	0.03
BXD 33TY	0.066	±	0.03	0.074	±	0.01	0.080	±	0.03	0.154	±	0.01	0.228	±	0.04
BXD 34TY	0.056	±	0.01	0.063	±	0.01	0.075	±	0.01	0.159	±	0.04	0.184	±	0.02
BXD 39TY	0.049	±	0.02	0.074	±	0.01	0.050	±	0.02	0.118	±	0.05	0.162	±	0.02
BXD 40TY	0.055	±	0.00	0.074	±	0.01	0.060	±	0.02	0.160	±	0.03	0.173	±	0.02
BXD 42TY	0.047	±	0.01	0.095	±	0.02	0.047	±	0.02	0.133	±	0.01	0.147	±	0.02
DBA2/J	0.037	±	0.01	0.079	±	0.01	0.034	±	0.00	0.093	±	0.01	0.128	±	0.02
C57BL/6 J	0.051	±	0.01	0.058	±	0.02	0.098	±	0.05	0.143	±	0.04	0.159	±	0.01

### MRNA expression correlations of miRNA expression

The correlations among hippocampal miRNA expression and genomewide mRNA expression, independent of the presence of a predicted miRNA target site, are summarized in Table 2 (for a complete list of nominally significant genes (p-value < 0.05) for each miRNA see Additional file 1: Table S1). We controlled the false detection rate for each gene list individually using the Benjamini and Hochberg method, q < 0.2 [23]. Only a single correlation remained significant following FDR multiple testing correction, which was the correlation between miR-15b and probe set 1437110_at (p-value = 2.6e-6, q = 0.04). This probe set corresponds to the gene *2810474O19Rik.*

**Table 2:** No increase in correlations between miRNA expression and mRNA expression for genes with miRNA target sites

**miRNA**	**Target sites**	**All correlations (p < 0.05)**			**Negative correlations (p < 0.05)**		
		**Target sites**	**Total**	**Chi square(Χ**^**2**^**,df, p-value)**	**Target sites**	**Total**	**Chi square(Χ**^**2**^**,df, p-value)**
*miR-15b*	526	5	541	(8.6,1,0.003)	4	302	(2.6,1,0.11)
*miR-31*	404	10	586	(1.1, 1, 0.30)	3	312	(0.1, 1, 0.15)
*miR-34c*	494	12	532	(0.7, 1, 0.39)	6	174	(0.7, 1, 0.82)
*miR-212*	519	17	858	(3.3, 1, 0.07)	3	271	(2.8, 1, 0.10)
*miR-301a*	456	26	927	(0.1, 1, 0.76)	7	362	(0.5, 1, 0.49)

The total number of probes investigated was the same for all five miRNAs (n = 17208).

Χ^2^ analysis was used to determine whether those genes with predicted miRNA binding sites were overrepresented within the list of nominally significant correlations between miRNA and mRNA. We used target sites predicted by the MIRANDA algorithm as this is one of the most widely used and has a higher sensitivity rate than the other commonly used algorithms [24]. As increases in miRNA expression can degrade the corresponding target mRNA, we conducted these analyses first using just the significant negative correlations, then using all significant correlations (summarized on Table 2). None of the miRNAs showed an overrepresentation of genes with predicted miRNA binding sites when either just the negative correlations or all correlations were used.

### KEGG pathway analysis

We used the list of all genes for which there were nominally significant correlations between miRNA vs. mRNA expression, independent of the presence of a miRNA target site, to conduct KEGG pathway analyses for each miRNA. We found a number of pathways that were significantly enhanced for these miRNAs (summarized on Table 3), including numerous neurobehavioural pathways. In particular, we found that genes correlated with miR-15b expression were enriched in a number of pathways related to neuronal plasticity, including axon guidance, long-term potentiation, long-term depression and regulation of the actin cytoskeleton.

**Table 3:** KEGG pathway analysis for those genes whose mRNA expression was significantly associated with miRNA expression (p < 0.05)

**miRNA**	**Correlated genes (n = 17208)**		**SIGNIFICANTLY ENHANCED KEGG PATHWAYS (4 gene threshold)**	
	**p < 0.05**	**q < 0.2**	**KEGG Pathway name**	**(observe, expected, p-value)**
**miR-15b**	541	1	Focal adhesion	(o = 10; e = 1.8; p = 2.8e-5)
			Regulation of actin cytoskeleton	(o = 10; e = 1.9; p = 4.2e-5)
			Long-term potentiation	(o = 5; e = 0.6; p = 6.3e-4)
			Pancreatic cancer	(o = 4; e = 0.7; p = 7.1e-3)
			Long-term depression	(o = 4; e = 0.7; p = 7.7e-3)
			Axon guidance	(o = 4; e = 1.3; p = 4.0e-2)
**miR-212**	858	0	MAPK signaling pathway	(o = 12; e = 3.8; p = 7.1e-4)
			Regulation og actin cytoskeleton	(o = 10; e = 2.8; p = 7.4e-4)
			Calcium signaling pathway	(o = 8; e = 2.4; p = 4.5e-3)
			Tight junction	(o = 8; e = 1.6; p = 3.2e-4)
**miR-34c**	532	0	MAPK signaling pathway	(o = 12; e = 2.7; p = 3.3e-5)
			Jak-STAT signaling pathway	(o = 7; e = 1.5; p = 9.9e-4)
			Focal adhesion	(o = 7; e = 1.9; p = 3.6e-3)
			Long-term depresion	(o = 4; e = 0.7; p = 8.4e-3)
**miR-31**	586	0	Regulation of actin cytoskeleton	(o = 11; e = 2.1; p = 1.5e-5)
			MAPK signaling pathway	(o = 10; e = 2.9; p = 8.5e-4)
			Axon guidance	(o = 9; e = 1.3; p = 1.5e-5)
			GnRH signaling pathway	(o = 7; e = 1.0; p = 1.0e-4)
			Wnt signaling pathway	(o = 6; e = 1.5; p = 5.5e-3)
**miR-301a**	927	0	Focal adhesion	(o = 13; e = 3.1; p = 2.5e-5)
			Regulation of actin cytoskeleton	(o = 11; e = 3.2; p = 6.0e-4)
			MAPK signaling pathway	(o = 10; e = 4.4; p = 1.6e-2)
			Colorectal cancer	(o = 7; e = 1.4; p = 6.5e-4)
			Long-term potentiation	(o = 4; e = 1.1; p = 2.5e-2)

### QTL analysis of miRNA expression

**Figure 1:**
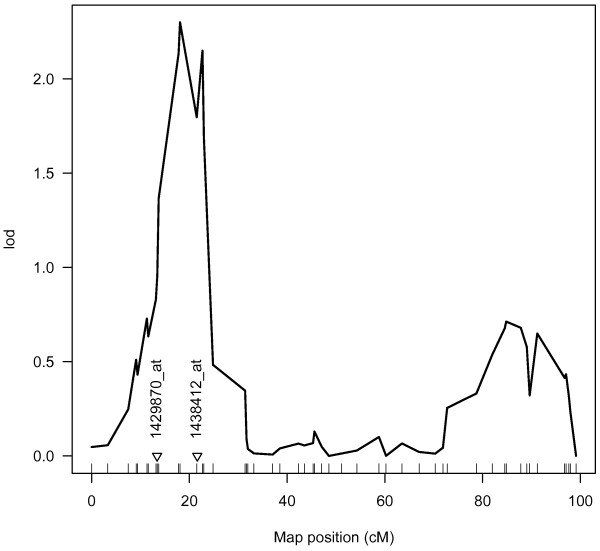
**eQTL peak for *miR-212 *on chromosome 3. ** The eQTL for * miR-212 * expression is shown for chromosome 3. The triangles on the x-axis represent the location of the probe sets that were significantly associated with *miR-212* expression (q < 0.2)

We conducted quantitative trait loci analysis of miRNA expression for each miRNA using R/QTL. We found a total of five suggestive QTLs which are summarized on Table 4. In order to further investigate what genes may be responsible for these QTLs, we conducted correlations of miRNA vs. mRNA gene expression for those genes within the 1.5 LOD confidence intervals for each of these peaks. We controlled the false detection rate for each gene list individually using the Benjamini and Hochberg method, q < 0.2 [23]. Two of these correlations survived multiple testing correction, 1429870_at (gene *Tnik*) and 1438412_at (gene *Phf17*), both lying underneath the expression QTL (eQTL) for miR-212 on chromosome 3 (see Figure 1). 

**Table 4:** eQTL peaks reaching suggestive significance

**miRNA**	**chr**	**position (Mb)**	**LOD score**	**1 LOD C.I.**	**1.5 LOD C.I.**
*miR-15b*	9	41.3	2.28	(24.4, 47.4)	(24.4, 119.2)
*miR-212*	3	37.3	2.29	(28.6, 46)	(27.5, 46)
*miR-301a*	1	20.6	2.82	(14.0, 96.9)	(11.1, 186.1)
*miR-301a*	11	19.4	2.37	(11.1, 22.9)	(4.4, 82.1)
*miR-301a*	17	69.2	2.30	(68.4, 89.9)	(57.5, 92.1)

The LOD score confidence intervals for a 1 LOD and 1.5 LOD score drop-off (LOD C.I. and 1.5 LOD C.I., respectively) are listed in Mb and were calculated using the lodint() function in the QTL package in R. We used the NCBI37 genome build for this analysis.

We examined whether there were any genes under these eQTL peaks that contained predicted miRNA binding sites. There were genes with predicted miRNA target sites under all of the eQTL peaks, except the *miR-301a* peak on chr11 (summarized in Additional file 2: Table S2). The gene *Acad9*, which codes for a mitochondrial Acyl-CoA dehydrogenase, had two predicted miR-212 target sites and was negatively correlated with miR-212 expression (r = -.446, p = .029). Neither of the genes, *Phf17* and *Tnik*, have a predicted miR-212 target site.

### Correlations of miRNA expression and phenotype

The correlations of miRNA expression and phenotype revealed a total of 69 nominally significant correlations (p < 0.05), summarized in Additional file 3: Table S3. Two of these correlations survived FDR multiple testing correction (q < 0.2), miR-31 expression and both blood ethanol concentration (following 2.25 mg/kg ethanol) (r = 0.7, p = 0.001) and percent time spent in the centre area of the open field test (r = 0.68, p = 0.002) (measures from [25]). In order to determine if the number of significant correlations within a behavioural category was overrepresented, we conducted Fishers Exact Tests which showed that behaviour traits in general were overrepresented with miR-31 expression (p < 0.05) and cocaine related traits were overrepresented with both *miR-34c* expression (p < 0.05) and miR-212 expression (p < 0.1) (see Table 5). 

**Table 5:** Summary of the significant correlations of miRNA expression with behaviour

**miRNA**	**Category**	**Number of traits**	
		**p < 0.05**	**q < 0.2**
*mIR-15b*	General Behaviour	3	0
*mIR-15b*	Morphine	1	0
*miR-31*	Cocaine	7	0
*miR-31*	Ethanol	1	1
*miR-31*	General Behaviour**	13	1
*miR-31*	Morphine	1	0
*miR-34c*	Cocaine**	7	0
*miR-34c*	General Behaviour	3	0
*miR-212*	Cocaine*	11	0
*miR-212*	Ethanol	1	0
*miR-212*	General Behaviour	11	0
*miR-301a*	General Behaviour	10	0

Categories with a Fisher’s exact test (one-way, overrepresented) of p < 0.05 are noted with a **, while categories with a Fisher’s exact test (one-way, overrepresented) of 0.05 < p < 0.1 are represented with a *.

## Discussion

BXD-RI lines have been successfully used to characterize the roles that genetic variation plays in various molecular and functional phenotypes, including gene expression, and thus in complex behaviour and disease [16,26,27]. The genetic and phenotypic variation across the RI strains, combined with extensive collections of cumulative data available in extensive web-based databases including gene expression data, genetic mapping panels and phenotypic data [28] underlies the utility of these reference strains in such characterization.

One of the greatest advantages of using BXD RI, and similar RI populations, is that existing dense genotypic data allows variation of any quantitative phenotype to be readily mapped using QTL mapping approaches, thus allowing for the discovery of putative local (cis) and distant (trans) genetic loci governing this variation. We were able to find five suggestive distant QTLs for the miRNAs investigated. Of particular interest was the QTL for miR-212 expression on chromosome 3, within which we found two expression probe sets that were significantly correlated with miR-212 expression. *Phf17*, PHD finger protein17 (also know as *Jade1*), has also been shown to be involved in WNT pathway signalling [29], particularly playing a role in anteroposterior axis development [30]. *Tnik*, TRAF2 and NCK-interacting protein kinase, is a member of the Ste20 group of kinases, known to be regulators of MAP kinase cascades [31]. It has previously been shown to be an activator of WNT target genes [32] and regulation of the cytoskeleton [33]. KEGG pathway analysis for co-expressed transcripts of miR-212 supports a role for *miR-212* in the regulation of the cytoskeleton and of MAPK kinase pathways, functions that overlap with those of *Tnik* and suggesting that the miR-212 and *Tnik* correlation may underlie a real functional relationship between these genes.

It should be noted that neither of the genes corresponding to these probe sets, *Phf17* and *Tnik*, have a predicted miR-212 binding site or any RNA genes within their transcripts. The existence of such a site is not required to posit a mechanism by which genetic variation in that a functional process can indirectly regulate the abundance of miR-212. Further studies are required to determine which genetic variant potentially underlies this correlation, and what genes and mechanisms this variant acts upon to indirectly regulate miR-212 expression.

We found that the gene *Acad9*, which lies under the QTL for miR-212 expression on chromosome 3, had two predicted miR-212 target sites. Furthermore its expression was also negatively correlated with miR-212 expression. This suggests that there is the potential for *Acad9* to both indirectly regulate miR-212 expression and in turn be directly regulated by miR-212 expression. While we do not expect to find such a mechanism for every QTL locus, this is an additional mechanism by which genetic variation could influence gene expression.

While we did find five suggestive distant QTLs, we did not find any local QTLs, indicating that polymorphisms in the miRNA we evaluated do not directly influence their abundance in the hippocampus. Two previous studies investigating liver miRNA expression found a local QTL for *miR-31* in BXD and F2 panels [21,22]. The failure to replicate this local QTL in the present study may be due to the use of a different tissue (hippocampus) in our study. A study conducted in human samples looking for eQTLs across numerous tissues found that while 30% of all eQTLs were shared across the three tissues investigated, 29% were tissue-specific [34].

We found significant correlations between both miR-34c and miR-212 expression and cocaine-related behaviours. This is particularly interesting for *miR-212*, which has previously been shown to control cocaine intake [35]. In particular, it was shown that striatal miR-212 expression is increased following extended cocaine use in rats and that increases in striatal *miR-212* expression leads to decreases in cocaine intake following extended access conditions. Furthermore, *miR-212* is known to be regulated by MeCP2, an important regulator of neuroplasticity, and to affect BDNF expression and neuroplasticity in postmitotic neurons [36]. As cocaine addiction is widely believed to result in changes in neurocircuitry [37], *miR-212* is an excellent candidate for susceptibility to cocaine addiction, further supported by a previous finding that miR-212 expression affects dendrite growth and arborisation [38]. Our present result augments this finding by demonstrating that genetic polymorphisms can cause phenotypic variation in this process. The detection of this relationship also demonstrates the utility of a systems genetics strategy for the discovery of specific molecular and functional roles of miRNAs.

We found significant correlations (q < 0.2) of miR-31 expression with both blood ethanol concentration and a measure of anxiety (percent time spent in the centre area of the open field test). We further found an overrepresentation of traits in general behaviour with miR-31 expression, with a particular number from open field and light-dark box measures, suggesting that *miR-31* may play a role in anxiety. These are the first indications of *miR-31* potentially being involved in alcohol or anxiety related traits. A number of miRNAs have previously been shown to be altered in the brain of alcoholics, including miR-15b, miR-34c and miR-301a [39]. This suggests that *miR-31* should be investigated further for a role in anxiety and susceptibility to alcohol use, but that it may not be associated with long term alcohol exposure.

The WNT signalling pathway has previously been associated with adult neurogenesis in the hippocampus [40]. Both the MAPK and WNT signalling pathways were enhanced within the KEGG pathway analysis for miR-31 expression. These pathways have been associated with *miR-31* in a previous KEGG pathway analysis in human tumour cells [41]. Over expression of miR-31 increased Wnt-5a expression, which adds further support for the involvement of *miR-31* in the WNT signalling pathway [42]. Together this suggests that *miR-31* may possibly play a role in adult neurogenesis via the WNT signalling pathway.

*MiR-301a* plays a role in wide range of cancers including lung cancer [43], breast cancer [44], pancreatic cancer [45]. KEGG analysis revealed that *miR-301a* was associated with colorectal cancer. The expression of miR-301a is altered in p53-deficient mice, a known tumor suppressor gene, further supporting its involvement in cancer [46].

A recent study has shown that *miR-34c* plays a role in anxiety at the level of the amygdala [47]. More specifically, this gene was upregulated following acute and chronic stress, and lenti-virus mediated overexpression of miR-34c in the amygdala induced anxiolytic behaviour after challenge. We found the miR-34c expression was positively correlated with open arm duration (r = 0.43, p = 0.03), a measure of anxiety. Additionally, miR-34c has been shown to reduce the cellular response to corticotrophin releasing factor receptor type 1 (*CRFR1*), possibly acting via a miR-34c target site on the CRFR1 mRNA [47]. Together these findings suggest a role for *miR-34c* in regulating the central stress response. This gene has also been shown to be elevated in the hippocampus of Alzheimer’s disease patients and the corresponding mouse models, and overexpressing miR-34c leads to memory impairment [48]. Together this data suggests a role for this gene in the mechanisms of anxiety and memory which should be further investigated.

*MiR-34c* has also been shown to be involved in various cancers [49-51]. More specifically, *miR-34c* is thought to act as a tumor suppressor as part of a negative feedback loop including *Myc* and *Mapkapk5*, part of the MAPK signalling pathway [52]. We found that the MAPK signalling pathway is enhanced for *miR-34c*, and while *miR-34c* was not significantly associated with *Mapkapk5* expression (r = -0.24, p = 0.27), it was with *Mapkapk3* (r = 0.55, p = 0.009).

Our KEGG pathway analysis for *miR-15b* suggests it plays a role in long-term potentiation, long-term depression and axon guidance. A study investigating the localization of miRNAs in sympathetic neurons revealed that miR-15b is more highly abundant in the distal axons compared to the cell bodies [53]. Taken together, these findings suggest a role for *miR-15b* in neuronal plasticity. Expression studies have also linked miR-15b expression with various cancers [46,54,55], including pancreatic cancer [56]. Similarly, we found an enhanced KEGG pathway for pancreatic cancer for this gene.

The only correlation between miRNA and mRNA expression that had a genome-wide significance level was between *miR-15b* and *2810474O19Rik* expression. *2810474O19Rik*, which has no predicted miR-15b binding sites, is thought to play a role in development, being expressed in both gonadal [57] and preimplantation mouse development [58]. This gene also has been suggested to play a role in cell potency, more specifically in pluripotent cell identity via an interaction with O*ct4*[59]. There is limited evidence that *miR-15b* plays a role in cell potency, with it being expressed in multipotent cells during osteogenic differentiation [60]. The expression of miR-15b was upregulated in the umbilical vein [61] and human placenta [62], but there is no known link between *miR-15b* and development.

Our study made use of existing mRNA data collected in independent mice. While this enables one to clearly ascribe correlation to genetic factors, each sample came from environmentally distinct mice. Thus, one may conclude that environmental variation in mRNA across these two population samples was sufficient to exceed the genetic variation accounted for by the loci we detected. Nonetheless, relevant co-expression was detected and future studies in which samples are collected from a single population for phenotype, miRNA and gene expression are warranted. This may be done in a genetic reference population to exploit the breadth of existing data or a large experimental cross or mapping population such as the Diversity Outbred to improve mapping power and precision.

In our study we investigated the potential effects of miRNAs on mRNA by correlating their gene expression. For this approach to be successful, we need a significant percentage of miRNAs to regulate their target genes via RNA degradations rather than by blocking translation, as blocking translation would not necessarily change the mRNA levels. Numerous groups have successfully used this approach to show that miRNAs do reduce the expression of a significant fraction of their targets [63-66]. This approach thus allows us to investigate the effects of miRNA on their potential gene targets for a great number of genes.

If a significant fraction of predicted miRNA target sites are real and if miRNAs commonly cause RNA degradation of their target genes then we should see an over-representation of miRNA vs. mRNA correlations for genes with predicted target sites. We failed to find this for the miRNAs that we investigated (both all correlated genes and just the negatively correlated genes). This generally held true in another study investigating liver miRNA expression in inbred and BXD RI strains [22]. It is possible that there is little degradation of mRNA transcripts by miRNA in mammals, and instead miRNAs block translation of the targeted mRNA. The difficulty in accurate prediction of miRNA binding sites due to the small size of the recognition sequences could also account for this result [5]. An alternative interpretation is that additional cellular processes, possibility including indirect mechanisms of miRNA gene regulation, act in an indirect fashion to modulate or mask the regulatory effects of miRNAs *in vivo*[66].

## Conclusions

Systems genetic analyses make use of naturally occurring genetic polymorphisms to simultaneously map sources of genetic variation and identify relations among biological entities across biological scale (in this case miRNA, mRNA and behaviour). Genetic analysis of miRNA expression and co-expression in the BXD recombinant inbred panel, takes advantage of the extensive cumulative data available for this panel, to further characterize five miRNAs that were previously shown to be differentially expressed in the BXD progenitor strains [20]. This is the first study of brain miRNA abundance in the BXD genetic reference panel, and it revealed that a well-characterized miRNA to behaviour relationship is subject to genetic control.

Using this approach, we conducted QTL analysis that enabled the detection of genetic loci that regulate the expression of these miRNAs. eQTLs that regulate miRNA abundance are a new mechanism by which genetic variation influences brain and behaviour. Analysis of one of these QTLs revealed a gene, *Tnik,* which may regulate the expression of a miRNA, a molecular pathway and a behavioural phenotype. Furthermore, we found evidence of genetic covariation of miR-212 abundance and cocaine related behaviours that is strongly supported by previous functional studies, demonstrating the value of this approach for discovery of new functional roles and downstream processes regulated by miRNA.

In summary, systems genetic analysis of miRNA abundance is a promising approach to discovery of the functional significance or particular miRNA in phenotypic variation and disease. With a simple profiling study across the original BXD RI strains, robust associations of miRNA to gene expression and behaviour can be detected. Simultaneous estimation of gene expression and miRNA abundance in the full BXD RI panel or high-power, high precision populations such as large experimental crosses and advanced mouse populations recently developed by the Complex Trait Consortium may yield many more such associations to bridge the gap between molecular discovery of miRNA and functional biology.

## Methods

### Animals

Hippocampal tissue was collected from 24 strains of C57BL/6 J x DBA/2 J recombinant inbred mice (BXD): BXD1/TyJ, BXD5/TyJ, BXD6/TyJ, BXD8/TyJ, BXD9/TyJ, BXD11/TyJ, BXD12/TyJ, BXD16/TyJ, BXD18/TyJ, BXD19/TyJ, BXD21/TyJ, BXD22/TyJ, BXD23/TyJ, BXD24/TyJ, BXD27/TyJ, BXD28/TyJ, BXD30/TyJ, BXD31/TyJ, BXD32/TyJ, BXD33/TyJ, BXD34/TyJ, BXD39/TyJ, BXD40/TyJ, BXD42/TyJ (n = 4 for all BXD strains except BXD30/TyJ where n = 2). The colony of BXD RI strains was maintained at the Institute of Psychiatry using original stocks purchased fromsbl;lll The Jackson Laboratory (Bar Harbor, ME, USA). All animals were males aged between 100-110 days when killed. After cervical dislocation, bilateral hippocampi were dissected in their entirety from fresh brains within two minutes from the time of death. Any connecting tissue was trimmed off and the hippocampi were immediately snap frozen on dry ice and stored at -80^0^C. All housing and experimental procedures were carried out in accordance with the UK Home Office *Animals (Scientific Procedures) Act 1986* under License PPL No. 70/5113.

### miRNA isolation

In order to obtain reliable levels of miRNA, total RNA was isolated from hippocampus using a miRNA isolation kit (Abmbion, Life Technologies, UK). The total RNA concentration was determined using a nanodrop ND-1000 (Thermo Fisher Scientific).

### Taqman real-time polymerase chain reactions (RT-PCR)

The mature miRNA expression for the five miRNAs *miR-15b, miR-31, miR-34c*, *miR-212,* and *miR-301a* were quantified in the individual BXD animals using Taqman RT-PCR assays (Applied Biosystems, Life Technologies, Foster City, CA, USA). The reverse transcription (RT) reactions were conducted using the miRNA reverse transcription primer specific for each assay, using the standard TaqMan® micro RNA Reverse Transcription protocol (http://www.appliedbiosystems.com, document #: 4364031). The RT-PCR reactions were performed in triplicate using 0.5 μl of 20x PCR Probe/Primer Mix, 1.5 μl of product from the RT reaction (diluted 1:10), 5 μl of 2x TaqMan Master Mix (No UNG) and 3 μls nuclease-free water. A sample minus reverse transcription buffer was used as a negative control for the RT-PCR reactions. (All of the negative controls used failed to reach threshold by 45 cycles).

Reactions were run on a 7900HT Fast real-Time PCR System (Applied Biosystems, Foster City, CA, USA) in 384 well format. RNU19, *miR-9* and *miR-99a* were used as controls for each of the test assays investigated in the individual samples. These assays were chosen as controls because their expression did not differ across strain and they had low variability within strain.

### Taqman RT-PCR statistical analysis

Relative expression was calculated using the standard 2$-\left(\Delta \left(\text{Ct}\right)\right)$ relative expression method in Microsoft Excel. The relative expression of the individual samples was normalized to the geometric mean of RNU19, *miR-9* and *miR-99a* (previously shown not to differ between C57BL/6 J and DBA 2 J, [20]). The *η*^2^ values were calculated from the genetic and environmental (error-term) sums of squares derived from a one-way ANOVA using the open source statistical program R.

### Correlations of behaviour and miRNA expression

Correlations were conducted between miRNA expression and the phenotypic measures from the behavioural batteries previously described in [67] (n = 40) and [25] (n = 243). We limited our analysis to those measures for which we had the corresponding phenotype and miRNA expression data in at least 18 BXD RI strains. Correlations were conducted using PASW Statistics 18, Release Version 18.0.0 (SPSS Inc., 2009, Chicago, IL, USA). False Discovery Rate control [23] was used to account for multiple testing (q < 0.2). For each miRNA, we conducted Fisher’s exact tests (one-sided) to determine if the number of significantly correlated behavioural traits within a given behavioural category was greater than that predicted by chance.

### Correlations of mRNA and miRNA expression

Lists of genes with MIRANDA predicted miRNA sites [68] were downloaded from the online miRNA database, miRBase (http://microrna.sanger.ac.uk/). Hippocampal mRNA expression for the BXD RI mice was obtained from the Hippocampus Expression Consortium [69]. The hippocampus, excluding most of the subiculum, from two to three animals was dissected and pooled for hybridization to a single Affymetrix M430 2.0 array. Raw microarray data was transformed using the PDNN, MAS5 and RMA methods. to 2*z* + 8, thus yielding a data set with a standard deviation of 2 and an overall mean of 8.

The expression data and predicted target site information were merged and correlations between miRNA and mRNA expression were conducted using the open source statistical program R [70]. False discovery rate [23] was used to account for multiple testing. KEGG pathway analysis was conducted using the lists of nominally significant genes for each miRNA using WebGestalt (http://bioinfo.vanderbilt.edu/webgestalt***).*** Chi squared tests for independence (two-sided without Yates correction) were calculated to see if there was a relationship between the presence of a target site and a significant correlation between mRNA and miRNA expression (p <0.05).

### QTL analysis of miRNA expression

QTL analysis was conducted for miRNA expression for those miRNAs investigated in the BXD RI strains (*miR-15b, miR-31, miR-34c*, *miR-212,* and *miR- 301a).* These analyses were done using the R-QTL package in the open source statistical program R as previously described in [71]. Confidence intervals (1 and 1.5 LOD score drops) were calculated for any suggestive QTL peaks. A series of correlations between miRNA expression and mRNA expression were conducted for all of the genes underlying all the suggestive eQTL peaks using PASW Statistics 18, Release Version 18.0.0 (SPSS Inc., 2009, Chicago, IL, USA). False discovery rate control [23] was used to account for multiple testing (q < 0.2).

## Competing interests

The authors declare that they have no competing interests.

## Authors’ contributions

MJP conducted the real-time PCRs, the correlational analysis for miRNA vs. mRNAs, the KEGG pathway analysis and drafted the manuscript. LS carried out the QTL analysis and helped conceive the study. LL, JLPC and CF assisted with the RNA extractions and the behavioural analysis from [67]. EJC and VMP assisted with the analysis and interpretation of behavioural data from [25]. CG helped with the real-time PCR analysis. WN and HL assisted with the conceiving and planning of the project. All authors read and approved the final manuscript.

## Supplementary Material

Additional file 1 **Table S1.** Summary of all significant correlations of miRNA expression and mRNA expression (p-values < 0.05). Click here for file

Additional file 2 **Table S2.** Genes with miRNA target sites under the QTL for miRNA gene expression. Click here for file

Additional file 3 **Table S3.** Summary of all significant correlations of miRNA expression and phenotype measures (p-values < 0.05). Click here for file
