# Supplementary material for: Genetic variation in hippocampal microRNA expression differences in C57BL/6 J X DBA/2 J (BXD) recombinant inbred mouse strains
Source: BMC Genomics. 2012 Sep 13;13:476. doi: 10.1186/1471-2164-13-476 (PMC3496628; doi:10.1186/1471-2164-13-476)
Supplement: Additional file 1 — Table S1. Summary of all significant correlations of miRNA expression and mRNA expression (p-values < 0.05). [file 1471-2164-13-476-S1.doc]

**Supplemental Table1**  Summary of all significant correlations of miRNA expression and mRNA expression (p-values < 0.05)

| **miRNA** | **probe** | **Gene** | **miR-15b-corr** | **miR-15b-p** |
| --- | --- | --- | --- | --- |
| miR-15b | 1437110_at | 2810474O19Rik | -0.823 | 0.000 |
| miR-15b | 1427334_s_at | 2810474O19Rik | -0.727 | 0.000 |
| miR-15b | 1444811_at | Sec62 | -0.715 | 0.000 |
| miR-15b | 1441843_s_at | 5230400M03Rik | -0.679 | 0.001 |
| miR-15b | 1426777_a_at | Wasl | -0.674 | 0.001 |
| miR-15b | 1460328_at | Brd3 | -0.660 | 0.001 |
| miR-15b | 1434031_at | Zfp692 | -0.650 | 0.001 |
| miR-15b | 1425072_at | Skp2 | -0.642 | 0.001 |
| miR-15b | 1460438_at | LOC100044319 /// Lysmd1 | -0.627 | 0.002 |
| miR-15b | 1415999_at | Hey1 | 0.621 | 0.002 |
| miR-15b | 1426843_at | Atg2a | -0.620 | 0.002 |
| miR-15b | 1452113_a_at | Rab23 | 0.619 | 0.002 |
| miR-15b | 1422863_s_at | Pdlim5 | 0.617 | 0.002 |
| miR-15b | 1417248_at | Ralbp1 | -0.615 | 0.002 |
| miR-15b | 1429096_at | 2810455D13Rik | -0.611 | 0.003 |
| miR-15b | 1423117_at | Pum1 | -0.608 | 0.003 |
| miR-15b | 1452673_at | Ranbp3 | -0.606 | 0.003 |
| miR-15b | 1440061_at | Rbx1 | -0.605 | 0.003 |
| miR-15b | 1437633_at | Ankrd11 /// LOC629623 | -0.603 | 0.003 |
| miR-15b | 1457041_at | NA | 0.600 | 0.003 |
| miR-15b | 1427418_a_at | Hif1a | 0.600 | 0.003 |
| miR-15b | 1456341_a_at | 2310051E17Rik /// Klf9 | -0.598 | 0.003 |
| miR-15b | 1452370_s_at | B230208H17Rik | -0.594 | 0.004 |
| miR-15b | 1457707_at | Mctp2 | 0.592 | 0.004 |
| miR-15b | 1443969_at | Irs2 | -0.587 | 0.004 |
| miR-15b | 1437675_at | C130026L21Rik /// Slc8a1 | -0.586 | 0.004 |
| miR-15b | 1454664_a_at | Eif5 /// LOC100047658 | -0.584 | 0.004 |
| miR-15b | 1456357_at | A930041I02Rik | 0.583 | 0.004 |
| miR-15b | 1451420_at | Ccdc47 | 0.583 | 0.004 |
| miR-15b | 1439028_at | Ufm1 | -0.580 | 0.005 |
| miR-15b | 1448623_at | Tmem123 | 0.574 | 0.005 |
| miR-15b | 1447933_at | Kif26a | -0.574 | 0.005 |
| miR-15b | 1437253_at | A630054L15Rik | 0.574 | 0.005 |
| miR-15b | 1418181_at | Ptp4a3 | -0.572 | 0.005 |
| miR-15b | 1445481_at | AI317158 | -0.571 | 0.005 |
| miR-15b | 1425331_at | Zfp106 | 0.570 | 0.006 |
| miR-15b | 1418144_a_at | Pip5k1a | -0.566 | 0.006 |
| miR-15b | 1430503_at | 6330522J23Rik | 0.566 | 0.006 |
| miR-15b | 1436896_at | Ints9 | -0.564 | 0.006 |
| miR-15b | 1436983_at | Crebbp | -0.563 | 0.006 |
| miR-15b | 1422320_x_at | Phxr5 | -0.563 | 0.006 |
| miR-15b | 1430875_a_at | Pak1ip1 | -0.563 | 0.006 |
| miR-15b | 1456795_at | D330027G24Rik | 0.561 | 0.007 |
| miR-15b | 1454683_at | Sfrs8 | -0.561 | 0.007 |
| miR-15b | 1419038_a_at | Csnk2a1 /// LOC100039026 | -0.560 | 0.007 |
| miR-15b | 1416702_at | Serpini1 | 0.560 | 0.007 |
| miR-15b | 1427060_at | Mapk3 | -0.558 | 0.007 |
| miR-15b | 1448443_at | Serpini1 | 0.557 | 0.007 |
| miR-15b | 1455013_at | Arih2 | -0.557 | 0.007 |
| miR-15b | 1428877_at | Srp72 | -0.554 | 0.007 |
| miR-15b | 1415859_at | Eif3c | -0.552 | 0.008 |
| miR-15b | 1417539_at | LOC100046775 /// Slc35a1 | 0.552 | 0.008 |
| miR-15b | 1455798_at | Galk2 | 0.551 | 0.008 |
| miR-15b | 1450534_x_at | H2-K1 | 0.551 | 0.008 |
| miR-15b | 1419978_s_at | D10Ertd610e | -0.550 | 0.008 |
| miR-15b | 1426727_s_at | LOC100039405 /// LOC677319 /// Ppp1r10 | -0.548 | 0.008 |
| miR-15b | 1421236_at | Ripk2 | 0.546 | 0.009 |
| miR-15b | 1429318_a_at | LOC100046895 /// Qk | -0.546 | 0.009 |
| miR-15b | 1439367_x_at | Arf4 | 0.546 | 0.009 |
| miR-15b | 1448244_at | Lypla1 | 0.545 | 0.009 |
| miR-15b | 1420396_at | Cd160 | 0.543 | 0.009 |
| miR-15b | 1428508_at | Tbc1d2b | -0.543 | 0.009 |
| miR-15b | 1423215_at | Spcs2 | 0.541 | 0.009 |
| miR-15b | 1451003_at | Map3k7ip2 | -0.541 | 0.009 |
| miR-15b | 1453146_at | Gapvd1 | -0.540 | 0.009 |
| miR-15b | 1447631_at | Myst2 | -0.540 | 0.009 |
| miR-15b | 1429489_at | Rexo1 | -0.539 | 0.010 |
| miR-15b | 1424743_at | 2610003J06Rik | -0.539 | 0.010 |
| miR-15b | 1415728_at | Pabpn1 | -0.538 | 0.010 |
| miR-15b | 1416035_at | Hif1a | 0.538 | 0.010 |
| miR-15b | 1450650_at | Myo10 | -0.537 | 0.010 |
| miR-15b | 1442064_at | AW556556 | -0.537 | 0.010 |
| miR-15b | 1423194_at | Arhgap5 | -0.535 | 0.010 |
| miR-15b | 1452100_at | Dullard /// LOC100048221 | -0.534 | 0.011 |
| miR-15b | 1435428_at | BC037112 | -0.533 | 0.011 |
| miR-15b | 1419574_at | Zfp292 | 0.533 | 0.011 |
| miR-15b | 1434054_at | Mafg | -0.532 | 0.011 |
| miR-15b | 1426805_at | Smarca4 | -0.530 | 0.011 |
| miR-15b | 1456054_a_at | Pum1 | -0.530 | 0.011 |
| miR-15b | 1437864_at | Adipor2 | -0.530 | 0.011 |
| miR-15b | 1435427_x_at | BC037112 | -0.530 | 0.011 |
| miR-15b | 1437849_x_at | Armcx2 | 0.529 | 0.011 |
| miR-15b | 1451274_at | Ogdh | -0.529 | 0.011 |
| miR-15b | 1417228_at | Capn1 | -0.529 | 0.011 |
| miR-15b | 1417377_at | Cadm1 | -0.528 | 0.011 |
| miR-15b | 1435259_s_at | Tmem141 | -0.528 | 0.011 |
| miR-15b | 1426631_at | LOC100047009 /// Pus7 | -0.528 | 0.012 |
| miR-15b | 1437061_at | Mbd1 | -0.528 | 0.012 |
| miR-15b | 1442116_at | Gpr176 | 0.528 | 0.012 |
| miR-15b | 1416536_at | Mum1 | -0.526 | 0.012 |
| miR-15b | 1429329_at | Cox10 | -0.526 | 0.012 |
| miR-15b | 1434228_at | Ppm2c | 0.526 | 0.012 |
| miR-15b | 1423449_a_at | Actn4 | -0.526 | 0.012 |
| miR-15b | 1448916_at | LOC100047868 /// Mafg | -0.526 | 0.012 |
| miR-15b | 1456355_s_at | Srrd | -0.525 | 0.012 |
| miR-15b | 1450937_at | Lin7c | 0.524 | 0.012 |
| miR-15b | 1438650_x_at | Gja1 | 0.524 | 0.012 |
| miR-15b | 1441197_at | 9530059O14Rik | -0.524 | 0.012 |
| miR-15b | 1424454_at | Tmem87a | -0.523 | 0.012 |
| miR-15b | 1429502_at | Stch | 0.523 | 0.013 |
| miR-15b | 1427379_at | Pnpla6 | -0.523 | 0.013 |
| miR-15b | 1443220_at | NA | -0.522 | 0.013 |
| miR-15b | 1427030_at | Ccdc52 | -0.521 | 0.013 |
| miR-15b | 1453734_at | Atrx | 0.521 | 0.013 |
| miR-15b | 1434383_at | Pja2 | 0.520 | 0.013 |
| miR-15b | 1447160_at | Nono | 0.519 | 0.013 |
| miR-15b | 1455644_at | Vps53 | -0.518 | 0.013 |
| miR-15b | 1424133_at | Tmem98 | 0.517 | 0.014 |
| miR-15b | 1426855_at | D10Ertd610e | -0.517 | 0.014 |
| miR-15b | 1421640_a_at | Tank | 0.517 | 0.014 |
| miR-15b | 1437224_at | Rtn4 | -0.517 | 0.014 |
| miR-15b | 1450874_at | Matr3 | 0.517 | 0.014 |
| miR-15b | 1435174_at | LOC100045795 /// Rsbn1 | 0.517 | 0.014 |
| miR-15b | 1444493_at | NA | -0.517 | 0.014 |
| miR-15b | 1424924_at | Sec63 | 0.516 | 0.014 |
| miR-15b | 1451020_at | Gsk3b | -0.516 | 0.014 |
| miR-15b | 1438476_a_at | Chd4 | -0.515 | 0.014 |
| miR-15b | 1449042_at | Ctcf | -0.514 | 0.014 |
| miR-15b | 1427906_at | 1110037F02Rik | 0.514 | 0.014 |
| miR-15b | 1426997_at | Thra | -0.513 | 0.015 |
| miR-15b | 1446644_at | NA | -0.513 | 0.015 |
| miR-15b | 1429066_at | 4930565B19Rik | -0.513 | 0.015 |
| miR-15b | 1427978_at | 4732418C07Rik | -0.513 | 0.015 |
| miR-15b | 1450814_a_at | Ipo4 | -0.512 | 0.015 |
| miR-15b | 1455131_at | Opa3 | -0.512 | 0.015 |
| miR-15b | 1428520_at | 1110032A13Rik | -0.511 | 0.015 |
| miR-15b | 1424500_at | Utp6 | 0.511 | 0.015 |
| miR-15b | 1437210_a_at | Brd2 | -0.511 | 0.015 |
| miR-15b | 1437728_at | Alkbh5 | -0.511 | 0.015 |
| miR-15b | 1450978_at | Dvl1 | -0.511 | 0.015 |
| miR-15b | 1422857_at | Trip4 | 0.510 | 0.015 |
| miR-15b | 1418288_at | Lpin1 | -0.510 | 0.015 |
| miR-15b | 1429588_at | 2810474O19Rik | -0.509 | 0.015 |
| miR-15b | 1452338_s_at | Itsn1 | -0.509 | 0.015 |
| miR-15b | 1452826_s_at | Fbxl20 | -0.509 | 0.015 |
| miR-15b | 1426221_at | Loh11cr2a | 0.509 | 0.016 |
| miR-15b | 1417084_at | Eif4ebp2 | -0.508 | 0.016 |
| miR-15b | 1436343_at | Chd4 | -0.508 | 0.016 |
| miR-15b | 1417987_at | Btd | 0.508 | 0.016 |
| miR-15b | 1417010_at | Zfp238 | 0.507 | 0.016 |
| miR-15b | 1452360_a_at | Jarid1a | -0.507 | 0.016 |
| miR-15b | 1427425_at | 9130208E07Rik | -0.506 | 0.016 |
| miR-15b | 1428126_a_at | 4921506J03Rik | -0.506 | 0.016 |
| miR-15b | 1444004_at | Thoc2 | -0.506 | 0.016 |
| miR-15b | 1460276_a_at | Gpr175 | -0.505 | 0.017 |
| miR-15b | 1426840_at | Ythdf3 | 0.505 | 0.017 |
| miR-15b | 1416668_at | Ttc35 | 0.504 | 0.017 |
| miR-15b | 1456544_at | Tmem38b | 0.504 | 0.017 |
| miR-15b | 1439336_at | NA | 0.503 | 0.017 |
| miR-15b | 1456066_a_at | Rpo1-4 | -0.503 | 0.017 |
| miR-15b | 1456022_at | Hipk2 | -0.503 | 0.017 |
| miR-15b | 1434020_at | Pdap1 | -0.503 | 0.017 |
| miR-15b | 1450086_at | Gmeb1 | -0.503 | 0.017 |
| miR-15b | 1436746_at | Wnk1 | -0.503 | 0.017 |
| miR-15b | 1444258_at | NA | -0.502 | 0.017 |
| miR-15b | 1442670_at | NA | 0.502 | 0.017 |
| miR-15b | 1449355_a_at | Eps15l1 | -0.502 | 0.017 |
| miR-15b | 1435885_s_at | Itsn1 | -0.501 | 0.017 |
| miR-15b | 1417376_a_at | Cadm1 | -0.501 | 0.018 |
| miR-15b | 1454923_at | Iws1 | 0.501 | 0.018 |
| miR-15b | 1438945_x_at | Gja1 | 0.500 | 0.018 |
| miR-15b | 1425780_a_at | Tmem167 | 0.500 | 0.018 |
| miR-15b | 1438419_at | Rbm16 | -0.499 | 0.018 |
| miR-15b | 1420397_a_at | Spen | -0.499 | 0.018 |
| miR-15b | 1417087_at | Glg1 | -0.499 | 0.018 |
| miR-15b | 1428289_at | 2310051E17Rik /// Klf9 | -0.499 | 0.018 |
| miR-15b | 1459657_s_at | LOC100039220 /// Rpo1-3 | 0.498 | 0.018 |
| miR-15b | 1460383_at | Gnao1 | 0.497 | 0.019 |
| miR-15b | 1440222_at | Sod1 | -0.496 | 0.019 |
| miR-15b | 1459854_s_at | Dynlt3 | 0.496 | 0.019 |
| miR-15b | 1457455_at | Suhw4 | -0.496 | 0.019 |
| miR-15b | 1433488_x_at | Gns | 0.496 | 0.019 |
| miR-15b | 1440467_at | 4922501C03Rik | -0.496 | 0.019 |
| miR-15b | 1415952_at | Mark2 | -0.495 | 0.019 |
| miR-15b | 1455356_at | Camsap1 | -0.494 | 0.020 |
| miR-15b | 1460649_at | Irak1 | -0.494 | 0.020 |
| miR-15b | 1448661_at | Plcb3 | 0.493 | 0.020 |
| miR-15b | 1415842_at | Gbl | -0.493 | 0.020 |
| miR-15b | 1420934_a_at | Srrm1 | -0.493 | 0.020 |
| miR-15b | 1448320_at | LOC100045432 /// Stim1 | -0.492 | 0.020 |
| miR-15b | 1434690_at | Lycat | 0.491 | 0.020 |
| miR-15b | 1421894_a_at | Tpp2 | 0.491 | 0.020 |
| miR-15b | 1415826_at | Atp6v1h | 0.490 | 0.021 |
| miR-15b | 1436805_at | Ubash3b | -0.490 | 0.021 |
| miR-15b | 1429497_s_at | Snx6 | 0.489 | 0.021 |
| miR-15b | 1434468_at | Otud4 | 0.488 | 0.021 |
| miR-15b | 1425845_a_at | Shoc2 | 0.488 | 0.021 |
| miR-15b | 1423403_at | Mapkbp1 | -0.487 | 0.022 |
| miR-15b | 1442305_at | Gtpbp2 | -0.486 | 0.022 |
| miR-15b | 1431232_a_at | Mga | 0.486 | 0.022 |
| miR-15b | 1416860_s_at | Ing1 | 0.486 | 0.022 |
| miR-15b | 1447939_a_at | 4933409K07Rik /// EG545605 /// LOC100039909 /// LOC100041516 /// LOC100041599 /// LOC100042524 /// LOC100042536 /// LOC665845 | -0.486 | 0.022 |
| miR-15b | 1416945_at | Ptov1 | -0.486 | 0.022 |
| miR-15b | 1424740_at | Creb3 | 0.486 | 0.022 |
| miR-15b | 1438504_x_at | Tm7sf3 | -0.486 | 0.022 |
| miR-15b | 1440280_at | Ccdc45 | -0.486 | 0.022 |
| miR-15b | 1443377_at | Adam1a | 0.485 | 0.022 |
| miR-15b | 1455307_at | BC037112 | -0.485 | 0.022 |
| miR-15b | 1416392_a_at | Atp6v0c /// Atp6v0c-ps2 | -0.485 | 0.022 |
| miR-15b | 1423052_at | Arf4 | 0.484 | 0.022 |
| miR-15b | 1421504_at | Sp4 | -0.484 | 0.022 |
| miR-15b | 1431081_a_at | Plscr3 | -0.483 | 0.023 |
| miR-15b | 1425195_a_at | Acat2 /// Acat3 | -0.483 | 0.023 |
| miR-15b | 1448334_a_at | Ccni | 0.482 | 0.023 |
| miR-15b | 1448570_at | Gmfb | 0.482 | 0.023 |
| miR-15b | 1450093_s_at | Zbtb7a | -0.482 | 0.023 |
| miR-15b | 1449405_at | Tns1 | -0.482 | 0.023 |
| miR-15b | 1438087_at | Tpmt | 0.482 | 0.023 |
| miR-15b | 1449516_a_at | Rgs3 | 0.481 | 0.023 |
| miR-15b | 1427254_at | Zfp445 | -0.481 | 0.023 |
| miR-15b | 1440253_at | Psmd11 | 0.481 | 0.023 |
| miR-15b | 1424628_a_at | 1500032D16Rik | -0.481 | 0.023 |
| miR-15b | 1433887_at | Dnajc3a | 0.481 | 0.024 |
| miR-15b | 1447349_s_at | Ep400 | -0.481 | 0.024 |
| miR-15b | 1457268_at | Dot1l | -0.480 | 0.024 |
| miR-15b | 1450424_a_at | Il18bp | 0.480 | 0.024 |
| miR-15b | 1455552_at | Snapc4 | -0.480 | 0.024 |
| miR-15b | 1429119_at | Iah1 | 0.480 | 0.024 |
| miR-15b | 1433719_at | Slc9a9 | -0.480 | 0.024 |
| miR-15b | 1418622_at | Rab2a | 0.479 | 0.024 |
| miR-15b | 1455714_at | Vstm2l | 0.479 | 0.024 |
| miR-15b | 1428659_at | Phf7 | 0.479 | 0.024 |
| miR-15b | 1439841_at | Zfyve27 | -0.478 | 0.025 |
| miR-15b | 1451124_at | Sod1 | -0.478 | 0.025 |
| miR-15b | 1420811_a_at | Ctnnb1 | 0.478 | 0.025 |
| miR-15b | 1433774_x_at | Cog1 | -0.478 | 0.025 |
| miR-15b | 1443436_at | NA | -0.478 | 0.025 |
| miR-15b | 1455183_at | Stk38l | 0.477 | 0.025 |
| miR-15b | 1416686_at | Plod2 | 0.477 | 0.025 |
| miR-15b | 1439826_at | Hspa14 | 0.477 | 0.025 |
| miR-15b | 1460316_at | Acsl1 | 0.477 | 0.025 |
| miR-15b | 1451519_at | Rnf2 | 0.477 | 0.025 |
| miR-15b | 1456131_x_at | Dag1 | -0.476 | 0.025 |
| miR-15b | 1455884_at | Dpp9 | -0.476 | 0.025 |
| miR-15b | 1452813_a_at | Tmem188 | -0.475 | 0.025 |
| miR-15b | 1448580_at | Glg1 | -0.475 | 0.025 |
| miR-15b | 1423792_a_at | Cmtm6 | 0.475 | 0.026 |
| miR-15b | 1418316_a_at | LOC100047588 /// Mark3 | -0.475 | 0.026 |
| miR-15b | 1428106_at | 1300001I01Rik | -0.475 | 0.026 |
| miR-15b | 1428971_at | Ccny /// LOC100044842 | 0.474 | 0.026 |
| miR-15b | 1426118_a_at | Tomm40 | -0.474 | 0.026 |
| miR-15b | 1420815_at | Gdi2 | 0.474 | 0.026 |
| miR-15b | 1430680_a_at | 5830417I10Rik /// LOC677582 | -0.474 | 0.026 |
| miR-15b | 1450026_a_at | B3gnt2 | 0.474 | 0.026 |
| miR-15b | 1416116_at | Orc3l | 0.473 | 0.026 |
| miR-15b | 1434821_at | Brd1 /// LOC100045983 | 0.473 | 0.026 |
| miR-15b | 1425267_a_at | Pear1 | 0.473 | 0.026 |
| miR-15b | 1428251_at | Smchd1 | 0.473 | 0.026 |
| miR-15b | 1434588_x_at | EG620313 /// Tbca | 0.472 | 0.026 |
| miR-15b | 1424239_at | 2310066E14Rik | -0.472 | 0.026 |
| miR-15b | 1447937_a_at | 4933409K07Rik /// EG545605 /// ENSMUSG00000073868 /// LOC100039909 /// LOC100041599 /// LOC100042524 /// LOC100042536 /// LOC665845 | -0.472 | 0.026 |
| miR-15b | 1433741_at | Cd38 | 0.472 | 0.027 |
| miR-15b | 1424604_s_at | Sumf1 | 0.472 | 0.027 |
| miR-15b | 1423141_at | Lipa | -0.472 | 0.027 |
| miR-15b | 1448109_a_at | Rpl26 | 0.472 | 0.027 |
| miR-15b | 1438771_at | Brd1 /// LOC100045983 | -0.471 | 0.027 |
| miR-15b | 1422242_at | Defcr-rs10 | 0.471 | 0.027 |
| miR-15b | 1447234_s_at | Snx6 | 0.470 | 0.027 |
| miR-15b | 1431485_at | 4833447I15Rik | 0.470 | 0.027 |
| miR-15b | 1452024_a_at | Ldb1 | -0.470 | 0.027 |
| miR-15b | 1424957_at | Ahdc1 | 0.470 | 0.027 |
| miR-15b | 1426875_s_at | Srxn1 | -0.470 | 0.027 |
| miR-15b | 1423829_at | 0910001A06Rik | 0.470 | 0.027 |
| miR-15b | 1424254_at | Ifitm1 | 0.469 | 0.028 |
| miR-15b | 1428156_at | Gng2 | 0.469 | 0.028 |
| miR-15b | 1433686_at | Cabin1 | -0.469 | 0.028 |
| miR-15b | 1434352_at | B630005N14Rik | 0.469 | 0.028 |
| miR-15b | 1438063_at | Mphosph9 | -0.469 | 0.028 |
| miR-15b | 1452801_at | Pigk | 0.468 | 0.028 |
| miR-15b | 1440254_at | LOC100041277 | -0.468 | 0.028 |
| miR-15b | 1435008_at | Slc9a6 | 0.468 | 0.028 |
| miR-15b | 1458710_at | NA | -0.468 | 0.028 |
| miR-15b | 1452102_at | Copb2 | 0.468 | 0.028 |
| miR-15b | 1444987_at | LOC100042978 | -0.467 | 0.028 |
| miR-15b | 1448579_at | Glg1 | -0.467 | 0.028 |
| miR-15b | 1448637_at | Med25 | -0.467 | 0.028 |
| miR-15b | 1429360_at | Klf3 /// LOC100046855 | -0.467 | 0.029 |
| miR-15b | 1437550_at | Dhx36 | 0.467 | 0.029 |
| miR-15b | 1437009_a_at | Zfp364 | 0.466 | 0.029 |
| miR-15b | 1417028_a_at | Trim2 | -0.466 | 0.029 |
| miR-15b | 1447883_x_at | Map1lc3a | -0.466 | 0.029 |
| miR-15b | 1425492_at | Bmpr1a | 0.466 | 0.029 |
| miR-15b | 1459439_at | Mrps5 | 0.465 | 0.029 |
| miR-15b | 1452737_at | 2810008M24Rik /// LOC100046418 | 0.465 | 0.029 |
| miR-15b | 1425523_at | Rbm25 | -0.465 | 0.029 |
| miR-15b | 1459838_s_at | Btbd11 | 0.464 | 0.029 |
| miR-15b | 1455922_at | Rab3gap1 | -0.464 | 0.030 |
| miR-15b | 1434707_at | Sbf1 | -0.464 | 0.030 |
| miR-15b | 1449388_at | Thbs4 | 0.464 | 0.030 |
| miR-15b | 1436796_at | Matr3 | -0.463 | 0.030 |
| miR-15b | 1429047_at | Rtf1 | 0.463 | 0.030 |
| miR-15b | 1415869_a_at | Trim28 | -0.463 | 0.030 |
| miR-15b | 1436316_at | 9430029L20Rik | -0.462 | 0.030 |
| miR-15b | 1426366_at | Eif2c2 | -0.462 | 0.030 |
| miR-15b | 1415773_at | Ncl | 0.462 | 0.030 |
| miR-15b | 1428097_at | 2510009E07Rik | 0.462 | 0.030 |
| miR-15b | 1452223_s_at | Gcap14 | 0.462 | 0.030 |
| miR-15b | 1455164_at | Cdgap | 0.462 | 0.030 |
| miR-15b | 1456740_x_at | Cog1 | -0.462 | 0.031 |
| miR-15b | 1423673_at | Ldoc1l | 0.461 | 0.031 |
| miR-15b | 1428411_at | 1700020I14Rik | 0.461 | 0.031 |
| miR-15b | 1448242_at | Sec61a1 | -0.460 | 0.031 |
| miR-15b | 1424684_at | Rab5c | -0.460 | 0.031 |
| miR-15b | 1415724_a_at | Cdc42 | 0.460 | 0.031 |
| miR-15b | 1457649_x_at | Ptchd1 | 0.460 | 0.031 |
| miR-15b | 1418628_at | Khdrbs1 | -0.460 | 0.031 |
| miR-15b | 1422910_s_at | Smc6 | -0.460 | 0.031 |
| miR-15b | 1439400_x_at | 5430433E21Rik | 0.459 | 0.031 |
| miR-15b | 1419370_a_at | Mfap1a /// Mfap1b | 0.459 | 0.032 |
| miR-15b | 1419470_at | Gnb4 | 0.458 | 0.032 |
| miR-15b | 1423858_a_at | Hmgcs2 | 0.458 | 0.032 |
| miR-15b | 1416369_at | Hiatl1 | 0.458 | 0.032 |
| miR-15b | 1457282_x_at | Tubgcp5 | -0.458 | 0.032 |
| miR-15b | 1451319_at | Senp1 | -0.458 | 0.032 |
| miR-15b | 1433518_at | Lcmt2 | 0.457 | 0.032 |
| miR-15b | 1429109_at | Msl2l1 | -0.457 | 0.032 |
| miR-15b | 1424442_a_at | Pja2 | -0.457 | 0.032 |
| miR-15b | 1457711_at | Ranbp3 | -0.457 | 0.033 |
| miR-15b | 1442079_at | Sgms1 | -0.457 | 0.033 |
| miR-15b | 1451587_a_at | Tiprl | 0.457 | 0.033 |
| miR-15b | 1437992_x_at | Gja1 | 0.457 | 0.033 |
| miR-15b | 1438705_at | Cbfa2t3 | -0.456 | 0.033 |
| miR-15b | 1456110_at | Ankrd11 /// LOC629623 | -0.456 | 0.033 |
| miR-15b | 1447320_x_at | LOC100039220 /// Rpo1-3 | 0.456 | 0.033 |
| miR-15b | 1438062_at | 4832420A03Rik /// Rsf1 | 0.456 | 0.033 |
| miR-15b | 1460211_a_at | Kdelr1 | -0.456 | 0.033 |
| miR-15b | 1439178_at | Adrbk2 | -0.455 | 0.033 |
| miR-15b | 1429487_at | Ppp1r12a | -0.455 | 0.033 |
| miR-15b | 1431277_at | Pla2g6 | 0.455 | 0.033 |
| miR-15b | 1426862_at | Aftph | -0.455 | 0.033 |
| miR-15b | 1423667_at | Mat2a | -0.455 | 0.034 |
| miR-15b | 1439016_x_at | Sprr2a | 0.455 | 0.034 |
| miR-15b | 1420956_at | Apc | 0.455 | 0.034 |
| miR-15b | 1437740_at | Plekhm2 | -0.454 | 0.034 |
| miR-15b | 1447612_x_at | NA | -0.454 | 0.034 |
| miR-15b | 1455252_at | Tsc1 | -0.454 | 0.034 |
| miR-15b | 1437200_at | Fcho2 | -0.453 | 0.034 |
| miR-15b | 1460269_at | Pnmt | -0.453 | 0.034 |
| miR-15b | 1460024_at | Tnrc6b | -0.453 | 0.034 |
| miR-15b | 1450984_at | Tjp2 | -0.453 | 0.034 |
| miR-15b | 1450848_at | Dap3 | -0.453 | 0.034 |
| miR-15b | 1422495_a_at | Hmgn1 /// LOC100044391 | 0.452 | 0.035 |
| miR-15b | 1416060_at | Tbc1d15 | 0.451 | 0.035 |
| miR-15b | 1441370_at | Tmcc1 | -0.451 | 0.035 |
| miR-15b | 1456587_x_at | 2010005J08Rik | -0.451 | 0.035 |
| miR-15b | 1422284_at | Nkx2-9 | 0.451 | 0.035 |
| miR-15b | 1417820_at | Tor1b | -0.451 | 0.035 |
| miR-15b | 1419945_s_at | Rab2a | 0.451 | 0.035 |
| miR-15b | 1418024_at | Narg1 | -0.451 | 0.035 |
| miR-15b | 1423451_at | Pgrmc1 | 0.451 | 0.035 |
| miR-15b | 1426681_at | Unk | -0.451 | 0.035 |
| miR-15b | 1433618_at | C330006A16Rik | -0.450 | 0.036 |
| miR-15b | 1423314_s_at | Pde7a | 0.450 | 0.036 |
| miR-15b | 1456471_x_at | EG627427 /// EG666422 /// EG666875 /// EG668771 /// LOC385344 /// LOC630761 /// LOC630896 /// LOC637235 /// LOC665516 /// LOC668506 /// LOC668576 /// LOC675316 /// Phgdh | -0.450 | 0.036 |
| miR-15b | 1460004_x_at | Stx6 | 0.450 | 0.036 |
| miR-15b | 1416629_at | Slc1a5 | -0.450 | 0.036 |
| miR-15b | 1451615_at | BC026374 | 0.449 | 0.036 |
| miR-15b | 1450083_at | Cnot4 | -0.449 | 0.036 |
| miR-15b | 1421841_at | Fgfr3 | 0.449 | 0.036 |
| miR-15b | 1418656_at | Lsm5 | 0.448 | 0.036 |
| miR-15b | 1441594_at | NA | -0.448 | 0.036 |
| miR-15b | 1438580_at | Zcchc7 | 0.448 | 0.036 |
| miR-15b | 1422848_a_at | Pabpn1 | -0.448 | 0.036 |
| miR-15b | 1447818_x_at | Rhebl1 | 0.448 | 0.036 |
| miR-15b | 1416974_at | Stam2 | 0.448 | 0.037 |
| miR-15b | 1435039_a_at | Pip5k1a | -0.447 | 0.037 |
| miR-15b | 1436422_at | BC026590 | 0.447 | 0.037 |
| miR-15b | 1439460_a_at | Arfgap2 | 0.447 | 0.037 |
| miR-15b | 1423834_s_at | Gga1 | -0.447 | 0.037 |
| miR-15b | 1416320_at | Sec22a | 0.447 | 0.037 |
| miR-15b | 1417891_at | Spsb3 | -0.447 | 0.037 |
| miR-15b | 1440392_at | Akap13 | -0.447 | 0.037 |
| miR-15b | 1436330_x_at | EG631624 | 0.446 | 0.037 |
| miR-15b | 1417974_at | Kpna4 | 0.446 | 0.037 |
| miR-15b | 1437908_a_at | Ergic1 | 0.446 | 0.037 |
| miR-15b | 1417378_at | Cadm1 | -0.446 | 0.037 |
| miR-15b | 1427460_at | LOC100046932 /// Taf4a | -0.446 | 0.037 |
| miR-15b | 1438246_at | Csnk1g1 | -0.446 | 0.037 |
| miR-15b | 1436389_at | NA | 0.446 | 0.037 |
| miR-15b | 1416979_at | Pomp | 0.446 | 0.038 |
| miR-15b | 1453189_at | Ube2i | -0.446 | 0.038 |
| miR-15b | 1418250_at | Arl4d /// LOC100044157 | 0.446 | 0.038 |
| miR-15b | 1438557_x_at | Dnpep | 0.445 | 0.038 |
| miR-15b | 1420445_at | Slc16a8 | 0.445 | 0.038 |
| miR-15b | 1419263_a_at | Adrm1 | -0.445 | 0.038 |
| miR-15b | 1442881_at | NA | 0.445 | 0.038 |
| miR-15b | 1422673_at | Prkd1 | 0.445 | 0.038 |
| miR-15b | 1460593_at | Susd4 | 0.445 | 0.038 |
| miR-15b | 1436860_at | Senp7 | 0.445 | 0.038 |
| miR-15b | 1448333_at | Adprh | 0.445 | 0.038 |
| miR-15b | 1458200_at | NA | -0.444 | 0.038 |
| miR-15b | 1452329_at | Plekhn1 | 0.444 | 0.038 |
| miR-15b | 1418927_a_at | Habp4 | -0.444 | 0.038 |
| miR-15b | 1425975_a_at | Mapk8ip3 | -0.444 | 0.038 |
| miR-15b | 1456316_a_at | Acbd3 | -0.444 | 0.038 |
| miR-15b | 1441338_at | 5930412G12Rik | 0.444 | 0.038 |
| miR-15b | 1455905_at | 2610507B11Rik | -0.444 | 0.038 |
| miR-15b | 1416440_at | Cd164 | 0.444 | 0.039 |
| miR-15b | 1434066_at | Gtf3c1 | -0.444 | 0.039 |
| miR-15b | 1451025_at | Arl1 | 0.443 | 0.039 |
| miR-15b | 1445411_at | NA | -0.443 | 0.039 |
| miR-15b | 1423758_at | G3bp2 | 0.443 | 0.039 |
| miR-15b | 1456118_at | Mettl2 | -0.443 | 0.039 |
| miR-15b | 1420351_at | Tnfrsf4 | 0.443 | 0.039 |
| miR-15b | 1419313_at | Ccnt1 /// LOC100047121 | -0.443 | 0.039 |
| miR-15b | 1430293_a_at | Fdx1l /// Glp1 | -0.443 | 0.039 |
| miR-15b | 1441945_s_at | Abhd14a | -0.442 | 0.039 |
| miR-15b | 1440444_at | Fads1 | -0.442 | 0.039 |
| miR-15b | 1418520_at | LOC100038890 /// Tgoln1 | 0.442 | 0.039 |
| miR-15b | 1433993_at | 4931406P16Rik | -0.442 | 0.039 |
| miR-15b | 1456868_at | NA | -0.442 | 0.040 |
| miR-15b | 1418381_at | Zfp148 | 0.442 | 0.040 |
| miR-15b | 1432403_at | 4933402C06Rik | 0.442 | 0.040 |
| miR-15b | 1418210_at | Pfn2 | 0.442 | 0.040 |
| miR-15b | 1431400_a_at | Gas7 | 0.441 | 0.040 |
| miR-15b | 1427349_x_at | 2810021G02Rik | 0.441 | 0.040 |
| miR-15b | 1455955_s_at | Snx17 | 0.441 | 0.040 |
| miR-15b | 1439726_at | Tmem186 | 0.441 | 0.040 |
| miR-15b | 1420000_s_at | Igbp1 | 0.440 | 0.040 |
| miR-15b | 1447636_x_at | Tmco4 | 0.440 | 0.040 |
| miR-15b | 1436307_at | Myo9a | -0.440 | 0.040 |
| miR-15b | 1449211_at | Bpnt1 | 0.440 | 0.040 |
| miR-15b | 1424700_at | Tmem38b | 0.440 | 0.040 |
| miR-15b | 1456739_x_at | Armcx2 | 0.440 | 0.041 |
| miR-15b | 1426664_x_at | Slc45a3 | -0.439 | 0.041 |
| miR-15b | 1454611_a_at | Calm1 | 0.439 | 0.041 |
| miR-15b | 1430614_at | 4632415K11Rik | 0.439 | 0.041 |
| miR-15b | 1448370_at | Ulk1 | -0.439 | 0.041 |
| miR-15b | 1450035_a_at | Prpf40a | -0.439 | 0.041 |
| miR-15b | 1448505_at | C1d | 0.439 | 0.041 |
| miR-15b | 1437748_at | Fut11 | -0.439 | 0.041 |
| miR-15b | 1453174_at | 2310076G13Rik | 0.439 | 0.041 |
| miR-15b | 1438736_at | Thoc2 | -0.439 | 0.041 |
| miR-15b | 1418427_at | Kif5b | -0.439 | 0.041 |
| miR-15b | 1454992_at | Slc7a1 | -0.438 | 0.041 |
| miR-15b | 1422692_at | Sub1 | 0.438 | 0.042 |
| miR-15b | 1424598_at | Ddx6 | -0.438 | 0.042 |
| miR-15b | 1425273_s_at | Emp2 | 0.438 | 0.042 |
| miR-15b | 1426369_at | Mlstd2 | 0.437 | 0.042 |
| miR-15b | 1437729_at | EG665189 | -0.437 | 0.042 |
| miR-15b | 1419183_at | Papd4 | 0.437 | 0.042 |
| miR-15b | 1423619_at | Rasd1 | -0.437 | 0.042 |
| miR-15b | 1456205_x_at | Tbca | 0.437 | 0.042 |
| miR-15b | 1456381_x_at | Mcl1 | 0.436 | 0.042 |
| miR-15b | 1458322_x_at | NA | -0.436 | 0.042 |
| miR-15b | 1434643_at | Tbl1x | -0.436 | 0.042 |
| miR-15b | 1428347_at | Cyfip2 | 0.436 | 0.042 |
| miR-15b | 1423982_at | Fusip1 | 0.436 | 0.043 |
| miR-15b | 1447046_at | NA | -0.436 | 0.043 |
| miR-15b | 1418835_at | Phlda1 | 0.436 | 0.043 |
| miR-15b | 1424814_a_at | Bcl2l14 | 0.436 | 0.043 |
| miR-15b | 1419189_at | Vti1a | 0.435 | 0.043 |
| miR-15b | 1451005_at | Sumo1 | 0.435 | 0.043 |
| miR-15b | 1451207_at | Cbara1 | -0.435 | 0.043 |
| miR-15b | 1455064_at | Rab36 | -0.435 | 0.043 |
| miR-15b | 1449001_at | Ivd | 0.435 | 0.043 |
| miR-15b | 1455871_s_at | LOC100039683 /// LOC100045937 /// Rpl13 /// Tax1bp3 | 0.435 | 0.043 |
| miR-15b | 1431189_a_at | Fahd2a | -0.435 | 0.043 |
| miR-15b | 1455089_at | Gng12 | 0.434 | 0.043 |
| miR-15b | 1435067_at | B230208H17Rik | -0.434 | 0.043 |
| miR-15b | 1455071_at | Zbtb7b | -0.434 | 0.043 |
| miR-15b | 1428617_at | Hcfc2 | -0.434 | 0.044 |
| miR-15b | 1431997_at | 3000002C10Rik | 0.434 | 0.044 |
| miR-15b | 1429152_at | Zkscan1 | 0.434 | 0.044 |
| miR-15b | 1455351_at | 2610101N10Rik | -0.434 | 0.044 |
| miR-15b | 1423162_s_at | Spred1 | -0.434 | 0.044 |
| miR-15b | 1448315_a_at | Pycr2 | -0.434 | 0.044 |
| miR-15b | 1452252_at | Utp20 | -0.433 | 0.044 |
| miR-15b | 1446385_at | 9430083A17Rik | 0.433 | 0.044 |
| miR-15b | 1452694_at | Ihpk1 | -0.433 | 0.044 |
| miR-15b | 1437995_x_at | sept7 | 0.433 | 0.044 |
| miR-15b | 1415752_at | BC031181 | -0.433 | 0.044 |
| miR-15b | 1415800_at | Gja1 | 0.432 | 0.044 |
| miR-15b | 1419248_at | Rgs2 | 0.432 | 0.045 |
| miR-15b | 1426389_at | Camk1d | -0.432 | 0.045 |
| miR-15b | 1436505_at | Ppig | -0.432 | 0.045 |
| miR-15b | 1457324_at | NA | -0.432 | 0.045 |
| miR-15b | 1430369_at | Epb4.1 | -0.432 | 0.045 |
| miR-15b | 1418627_at | Gclm | -0.432 | 0.045 |
| miR-15b | 1433765_at | Ube2o | -0.431 | 0.045 |
| miR-15b | 1423849_a_at | Clk3 | -0.431 | 0.045 |
| miR-15b | 1417571_at | Mpg | -0.431 | 0.045 |
| miR-15b | 1429539_at | Bcl2l13 | -0.431 | 0.045 |
| miR-15b | 1456716_s_at | 3110002H16Rik | -0.431 | 0.045 |
| miR-15b | 1445718_at | NA | -0.431 | 0.045 |
| miR-15b | 1447705_at | Nsl1 | 0.431 | 0.045 |
| miR-15b | 1443213_at | Gtdc1 | 0.431 | 0.045 |
| miR-15b | 1426725_s_at | Ets1 | 0.431 | 0.045 |
| miR-15b | 1454646_at | Tcp11l2 | -0.430 | 0.046 |
| miR-15b | 1456102_a_at | Cul5 | -0.430 | 0.046 |
| miR-15b | 1451380_at | Zfyve19 | 0.430 | 0.046 |
| miR-15b | 1427199_at | Fryl | -0.430 | 0.046 |
| miR-15b | 1416731_at | Top2b | 0.429 | 0.046 |
| miR-15b | 1451803_a_at | Vegfb | -0.429 | 0.046 |
| miR-15b | 1422539_at | Extl2 | 0.429 | 0.046 |
| miR-15b | 1417270_at | Wdr12 | -0.429 | 0.046 |
| miR-15b | 1436077_a_at | Fcho1 | -0.429 | 0.046 |
| miR-15b | 1418633_at | Notch1 | -0.429 | 0.046 |
| miR-15b | 1423656_x_at | 1500010J02Rik | -0.429 | 0.047 |
| miR-15b | 1417000_at | Abtb1 | -0.429 | 0.047 |
| miR-15b | 1422506_a_at | Cstb | 0.428 | 0.047 |
| miR-15b | 1450409_a_at | 4930570C03Rik | -0.428 | 0.047 |
| miR-15b | 1454722_at | Pten | 0.428 | 0.047 |
| miR-15b | 1452346_at | B3gnt1 | 0.428 | 0.047 |
| miR-15b | 1431020_a_at | Fgfr1op2 | -0.428 | 0.047 |
| miR-15b | 1448782_at | Txndc11 | -0.428 | 0.047 |
| miR-15b | 1418433_at | Cab39 | 0.428 | 0.047 |
| miR-15b | 1454675_at | Thra | -0.428 | 0.047 |
| miR-15b | 1439477_at | Ube2b | -0.427 | 0.047 |
| miR-15b | 1429415_at | Prkcbp1 | -0.427 | 0.047 |
| miR-15b | 1423875_at | AI450540 /// LOC100044843 | -0.427 | 0.047 |
| miR-15b | 1429384_at | Csnk1g3 /// LOC100047516 | 0.427 | 0.047 |
| miR-15b | 1419186_a_at | St8sia4 | 0.426 | 0.048 |
| miR-15b | 1424048_a_at | Cyb5r1 | -0.426 | 0.048 |
| miR-15b | 1417842_at | Caml | 0.426 | 0.048 |
| miR-15b | 1440332_at | Cdv3 | 0.426 | 0.048 |
| miR-15b | 1428957_at | Tmem177 | 0.426 | 0.048 |
| miR-15b | 1453821_at | N6amt1 | -0.426 | 0.048 |
| miR-15b | 1424741_s_at | Creb3 | 0.426 | 0.048 |
| miR-15b | 1423210_a_at | Nola3 | 0.426 | 0.048 |
| miR-15b | 1428734_at | 3200002M19Rik | 0.425 | 0.048 |
| miR-15b | 1446957_s_at | N4bp1 | -0.425 | 0.048 |
| miR-15b | 1452837_at | Lpin2 | -0.425 | 0.048 |
| miR-15b | 1415694_at | Wars | 0.425 | 0.049 |
| miR-15b | 1454614_at | 1810013D10Rik | 0.425 | 0.049 |
| miR-15b | 1435233_at | Ncoa2 | 0.425 | 0.049 |
| miR-15b | 1422858_at | Trip4 | 0.424 | 0.049 |
| miR-15b | 1448828_at | Smc6 | 0.424 | 0.049 |
| miR-15b | 1455157_a_at | BC039210 | -0.424 | 0.049 |
| miR-15b | 1428791_at | Ube2h | -0.424 | 0.049 |
| miR-15b | 1428926_at | 1110003O08Rik | -0.424 | 0.049 |
| miR-15b | 1418258_s_at | Dynll2 | -0.424 | 0.050 |
| miR-15b | 1456540_s_at | Mtmr6 | 0.423 | 0.050 |
| miR-15b | 1422881_s_at | Sypl | 0.423 | 0.050 |
| miR-15b | 1455870_at | Akap2 /// Palm2 /// Palm2-akap2 | 0.423 | 0.050 |
| miR-15b | 1424380_at | Vps37b | -0.423 | 0.050 |
| miR-15b | 1416663_at | Ndufa9 | 0.423 | 0.050 |
| miR-15b | 1430205_a_at | Cdc37l1 | 0.423 | 0.050 |
| miR-15b | 1417260_at | U2af2 | -0.423 | 0.050 |
| miR-15b | 1426354_at | Bap1 | -0.423 | 0.050 |
| miR-15b | 1415888_at | Hdgf | -0.423 | 0.050 |
|  |  | Gene.Symbol | miR-31-corr | miR-31-p |
| miR-31 | 1447953_at | Egfl8 | -0.744 | 0.000 |
| miR-31 | 1431132_x_at | 0610007P22Rik | 0.695 | 0.000 |
| miR-31 | 1416347_at | Men1 | -0.693 | 0.000 |
| miR-31 | 1416597_at | Hdgfrp2 | -0.682 | 0.000 |
| miR-31 | 1432827_x_at | Gm1821 /// LOC100048105 /// Rps27a /// Ubb /// Ubc | -0.681 | 0.000 |
| miR-31 | 1429610_a_at | Zfp511 | -0.679 | 0.001 |
| miR-31 | 1437389_x_at | Khdrbs1 | -0.677 | 0.001 |
| miR-31 | 1454931_at | Eid2 | -0.674 | 0.001 |
| miR-31 | 1449586_at | Pkp1 | 0.674 | 0.001 |
| miR-31 | 1424189_at | Pigc | 0.663 | 0.001 |
| miR-31 | 1416675_s_at | Plcd1 | -0.655 | 0.001 |
| miR-31 | 1426860_at | Ep400 | 0.647 | 0.001 |
| miR-31 | 1419040_at | Cyp2d22 | 0.641 | 0.001 |
| miR-31 | 1433912_at | Tiprl | 0.640 | 0.001 |
| miR-31 | 1416006_at | Mdk | -0.639 | 0.001 |
| miR-31 | 1422687_at | Nras | 0.637 | 0.001 |
| miR-31 | 1434541_x_at | Khdrbs1 | -0.634 | 0.002 |
| miR-31 | 1441900_x_at | Hspbap1 | 0.632 | 0.002 |
| miR-31 | 1449343_s_at | Sin3a | 0.630 | 0.002 |
| miR-31 | 1449664_s_at | Rnf20 | 0.629 | 0.002 |
| miR-31 | 1458931_at | 6030427F01Rik | -0.629 | 0.002 |
| miR-31 | 1416821_at | Es2el | -0.629 | 0.002 |
| miR-31 | 1430575_a_at | Tpp2 | -0.617 | 0.002 |
| miR-31 | 1418017_at | Pum2 | -0.616 | 0.002 |
| miR-31 | 1426793_a_at | LOC100040970 /// LOC100046610 /// Rpl14 | -0.615 | 0.002 |
| miR-31 | 1454717_at | Ankrd27 | 0.612 | 0.002 |
| miR-31 | 1444206_at | Zfp595 | 0.602 | 0.003 |
| miR-31 | 1459773_x_at | Snip1 | 0.602 | 0.003 |
| miR-31 | 1450799_at | Adcyap1r1 | -0.602 | 0.003 |
| miR-31 | 1431755_a_at | Ccdc49 | -0.601 | 0.003 |
| miR-31 | 1418300_a_at | Mknk2 | -0.599 | 0.003 |
| miR-31 | 1423783_at | Tor2a | -0.597 | 0.003 |
| miR-31 | 1441157_at | C230006B20 | 0.595 | 0.004 |
| miR-31 | 1456364_at | C230057M02Rik | 0.594 | 0.004 |
| miR-31 | 1422514_at | Aebp1 | -0.592 | 0.004 |
| miR-31 | 1418629_a_at | Khdrbs1 | -0.590 | 0.004 |
| miR-31 | 1417431_a_at | Sphk2 | 0.590 | 0.004 |
| miR-31 | 1426835_at | Metap1 | -0.588 | 0.004 |
| miR-31 | 1416145_at | Dhx15 | -0.588 | 0.004 |
| miR-31 | 1437216_at | Ccdc88a | -0.586 | 0.004 |
| miR-31 | 1419512_at | Prpf40b | -0.585 | 0.004 |
| miR-31 | 1416845_at | Tmem132a | -0.583 | 0.004 |
| miR-31 | 1417959_at | Pdlim7 | -0.583 | 0.004 |
| miR-31 | 1416074_a_at | LOC100039731 /// LOC100042670 /// LOC100047349 /// LOC100048222 /// Rpl28 | -0.578 | 0.005 |
| miR-31 | 1443826_x_at | Men1 | -0.577 | 0.005 |
| miR-31 | 1441855_x_at | Cxcl1 | 0.575 | 0.005 |
| miR-31 | 1417840_at | 1500031L02Rik | 0.573 | 0.005 |
| miR-31 | 1419753_at | Nfx1 | 0.573 | 0.005 |
| miR-31 | 1425900_at | Hkdc1 | 0.571 | 0.005 |
| miR-31 | 1428110_x_at | Vps11 | -0.571 | 0.006 |
| miR-31 | 1449442_at | Pex11a | -0.570 | 0.006 |
| miR-31 | 1446903_at | NA | 0.569 | 0.006 |
| miR-31 | 1441323_at | NA | -0.567 | 0.006 |
| miR-31 | 1454119_at | Mknk1 | -0.566 | 0.006 |
| miR-31 | 1415919_at | Npdc1 | -0.565 | 0.006 |
| miR-31 | 1451736_a_at | Map2k7 | -0.563 | 0.006 |
| miR-31 | 1458839_at | Exoc8 | -0.561 | 0.007 |
| miR-31 | 1453117_at | Surf2 | -0.561 | 0.007 |
| miR-31 | 1429694_at | 4930402H24Rik | -0.561 | 0.007 |
| miR-31 | 1438714_at | NA | -0.560 | 0.007 |
| miR-31 | 1448725_at | Parg | 0.560 | 0.007 |
| miR-31 | 1431131_s_at | A630007B06Rik | 0.559 | 0.007 |
| miR-31 | 1435684_at | Abcc5 | -0.559 | 0.007 |
| miR-31 | 1449942_a_at | Ilk | -0.558 | 0.007 |
| miR-31 | 1432842_s_at | LOC100039786 /// Ywhaq | -0.557 | 0.007 |
| miR-31 | 1456037_x_at | Preb | -0.556 | 0.007 |
| miR-31 | 1429153_at | 6530406A20Rik | -0.555 | 0.007 |
| miR-31 | 1451291_at | Obfc2b | 0.555 | 0.007 |
| miR-31 | 1422931_at | Fosl2 | 0.554 | 0.007 |
| miR-31 | 1434845_at | NA | 0.554 | 0.007 |
| miR-31 | 1426718_at | Skiv2l2 | -0.553 | 0.008 |
| miR-31 | 1433651_at | Wtip | -0.552 | 0.008 |
| miR-31 | 1422554_at | Ndnl2 | 0.552 | 0.008 |
| miR-31 | 1450676_at | Tceb3 | -0.552 | 0.008 |
| miR-31 | 1438675_at | Sfrs8 | -0.551 | 0.008 |
| miR-31 | 1430736_at | 9030411M15Rik | -0.550 | 0.008 |
| miR-31 | 1428773_s_at | Bcor | 0.549 | 0.008 |
| miR-31 | 1419462_s_at | Gtl3 | -0.547 | 0.008 |
| miR-31 | 1439124_at | Wdr91 | 0.546 | 0.009 |
| miR-31 | 1436822_x_at | Hk1 /// LOC100040745 /// LOC100040929 /// LOC100042067 /// LOC100042583 /// LOC100043273 /// LOC100045527 /// LOC100045668 /// LOC100048040 /// LOC676276 /// Rpl17 | -0.545 | 0.009 |
| miR-31 | 1439058_at | Sfpq | 0.545 | 0.009 |
| miR-31 | 1430544_at | 5830404H04Rik | 0.544 | 0.009 |
| miR-31 | 1450759_at | Bmp6 | -0.544 | 0.009 |
| miR-31 | 1442146_at | NA | 0.543 | 0.009 |
| miR-31 | 1426192_at | Smarcd2 | -0.543 | 0.009 |
| miR-31 | 1429296_at | Rab10 | 0.543 | 0.009 |
| miR-31 | 1455422_x_at | Sept4 | -0.542 | 0.009 |
| miR-31 | 1441017_at | Zcchc14 | 0.542 | 0.009 |
| miR-31 | 1416348_at | Men1 | -0.541 | 0.009 |
| miR-31 | 1423912_at | Aspscr1 | -0.541 | 0.009 |
| miR-31 | 1447769_x_at | Amigo2 | 0.540 | 0.009 |
| miR-31 | 1426981_at | Pcsk6 | -0.540 | 0.010 |
| miR-31 | 1436394_at | Trim37 | -0.540 | 0.010 |
| miR-31 | 1422499_at | Lima1 | -0.539 | 0.010 |
| miR-31 | 1456083_x_at | Eif3c | -0.538 | 0.010 |
| miR-31 | 1426697_a_at | Lrpap1 | -0.538 | 0.010 |
| miR-31 | 1424480_s_at | Akt2 /// LOC100048123 | 0.538 | 0.010 |
| miR-31 | 1423985_at | Gng5 /// LOC100043507 /// LOC100047170 /// LOC100048410 | -0.538 | 0.010 |
| miR-31 | 1430301_at | Stxbp5 | 0.537 | 0.010 |
| miR-31 | 1451333_a_at | Acrbp | 0.536 | 0.010 |
| miR-31 | 1451550_at | Ephb3 | 0.536 | 0.010 |
| miR-31 | 1454789_x_at | Prpf6 | -0.534 | 0.010 |
| miR-31 | 1419982_s_at | NA | 0.534 | 0.010 |
| miR-31 | 1422569_at | Yy1 | -0.534 | 0.011 |
| miR-31 | 1419054_a_at | Ptpn21 | -0.534 | 0.011 |
| miR-31 | 1448446_at | Deaf1 | 0.533 | 0.011 |
| miR-31 | 1424593_at | Ecd | 0.533 | 0.011 |
| miR-31 | 1438462_x_at | Khdrbs1 | -0.532 | 0.011 |
| miR-31 | 1418873_at | Sfxn4 | 0.532 | 0.011 |
| miR-31 | 1449187_at | Pdgfa | -0.531 | 0.011 |
| miR-31 | 1451113_a_at | Ik | -0.531 | 0.011 |
| miR-31 | 1427111_s_at | Raver1 | -0.530 | 0.011 |
| miR-31 | 1456872_a_at | D230010M03Rik | 0.530 | 0.011 |
| miR-31 | 1454014_a_at | Mkks | 0.530 | 0.011 |
| miR-31 | 1450637_a_at | Aebp1 | -0.527 | 0.012 |
| miR-31 | 1447105_at | NA | 0.527 | 0.012 |
| miR-31 | 1423045_at | Ncbp2 | -0.527 | 0.012 |
| miR-31 | 1426247_at | Stk24 | -0.526 | 0.012 |
| miR-31 | 1451317_at | Ythdf2 | -0.526 | 0.012 |
| miR-31 | 1440858_at | Crkrs | 0.525 | 0.012 |
| miR-31 | 1428906_at | Gtf2h5 | 0.525 | 0.012 |
| miR-31 | 1440324_at | Mrpl19 | 0.524 | 0.012 |
| miR-31 | 1438198_at | Bri3bp | 0.523 | 0.012 |
| miR-31 | 1430650_at | Zfp191 | 0.523 | 0.013 |
| miR-31 | 1435234_at | Ncoa2 | -0.523 | 0.013 |
| miR-31 | 1417500_a_at | Tgm2 | -0.522 | 0.013 |
| miR-31 | 1437690_x_at | Csnk1d | 0.521 | 0.013 |
| miR-31 | 1432441_at | 4933413J09Rik | 0.519 | 0.013 |
| miR-31 | 1423958_a_at | Ttc33 | 0.519 | 0.013 |
| miR-31 | 1433890_a_at | Bat3 | -0.519 | 0.013 |
| miR-31 | 1435699_at | Ppm1l | 0.518 | 0.014 |
| miR-31 | 1448246_at | Hdac1 /// Hdac1-ps /// LOC100046039 | -0.518 | 0.014 |
| miR-31 | 1447805_s_at | Slu7 | -0.517 | 0.014 |
| miR-31 | 1444606_at | Efna2 | 0.517 | 0.014 |
| miR-31 | 1454406_at | 4930453J04Rik | 0.517 | 0.014 |
| miR-31 | 1442799_x_at | Nudcd3 | 0.517 | 0.014 |
| miR-31 | 1433881_at | Dnajc11 | 0.516 | 0.014 |
| miR-31 | 1448672_a_at | Arfgap2 | 0.516 | 0.014 |
| miR-31 | 1458393_at | Srr | 0.516 | 0.014 |
| miR-31 | 1424216_a_at | Papola | -0.516 | 0.014 |
| miR-31 | 1421216_a_at | Ids | 0.515 | 0.014 |
| miR-31 | 1439535_at | NA | 0.515 | 0.014 |
| miR-31 | 1420494_x_at | Gm1821 /// LOC100048105 /// Rps27a /// Ubb /// Ubc | -0.514 | 0.014 |
| miR-31 | 1456398_at | Tug1 | -0.514 | 0.014 |
| miR-31 | 1451197_s_at | Gatad2a | -0.514 | 0.014 |
| miR-31 | 1440520_a_at | 1700051A21Rik | 0.514 | 0.014 |
| miR-31 | 1437350_at | RP23-143A14.5 | 0.514 | 0.014 |
| miR-31 | 1459573_at | Bxdc5 | 0.513 | 0.015 |
| miR-31 | 1429686_at | Polr3f | 0.513 | 0.015 |
| miR-31 | 1454042_a_at | Srpk1 | 0.513 | 0.015 |
| miR-31 | 1416477_at | LOC100041725 /// Ube2d2 | -0.513 | 0.015 |
| miR-31 | 1422453_at | Prpf8 | -0.512 | 0.015 |
| miR-31 | 1446776_at | C77581 | 0.512 | 0.015 |
| miR-31 | 1423639_at | Hrh2 | -0.512 | 0.015 |
| miR-31 | 1456731_x_at | Polr3k | 0.511 | 0.015 |
| miR-31 | 1424436_at | Gart | -0.511 | 0.015 |
| miR-31 | 1460498_a_at | Dnajc5 | -0.511 | 0.015 |
| miR-31 | 1417683_at | Diablo | 0.511 | 0.015 |
| miR-31 | 1433267_at | Ing1 | 0.511 | 0.015 |
| miR-31 | 1427441_a_at | Suclg2 | -0.511 | 0.015 |
| miR-31 | 1434229_a_at | Polb | -0.510 | 0.015 |
| miR-31 | 1448464_at | Ykt6 | 0.509 | 0.015 |
| miR-31 | 1460443_at | Brms1l | -0.509 | 0.016 |
| miR-31 | 1454418_at | 5330421C15Rik | 0.509 | 0.016 |
| miR-31 | 1455341_at | Eif1ad | 0.509 | 0.016 |
| miR-31 | 1448401_at | Smarcd2 | 0.509 | 0.016 |
| miR-31 | 1431242_at | 6330575P09Rik | 0.509 | 0.016 |
| miR-31 | 1423567_a_at | Psma7 | 0.508 | 0.016 |
| miR-31 | 1418295_s_at | Dgat1 | 0.508 | 0.016 |
| miR-31 | 1452649_at | Rtn4 | -0.508 | 0.016 |
| miR-31 | 1428299_at | Dyrk1a | -0.508 | 0.016 |
| miR-31 | 1445299_at | NA | 0.507 | 0.016 |
| miR-31 | 1448865_at | Hsd17b7 | 0.507 | 0.016 |
| miR-31 | 1460213_at | Golga4 | 0.507 | 0.016 |
| miR-31 | 1450560_a_at | Ppp2r5d | 0.506 | 0.016 |
| miR-31 | 1452694_at | Ihpk1 | 0.506 | 0.016 |
| miR-31 | 1416534_at | Dpf2 | -0.506 | 0.016 |
| miR-31 | 1436205_at | NA | -0.505 | 0.016 |
| miR-31 | 1437687_x_at | Fkbp9 | -0.505 | 0.016 |
| miR-31 | 1435696_s_at | Sfrs12ip1 | 0.505 | 0.016 |
| miR-31 | 1451232_at | Cd151 | -0.505 | 0.017 |
| miR-31 | 1443764_x_at | Rab27b | -0.505 | 0.017 |
| miR-31 | 1420596_at | Cacng2 | -0.505 | 0.017 |
| miR-31 | 1454983_at | B230380D07Rik | -0.504 | 0.017 |
| miR-31 | 1425931_a_at | Arntl2 | 0.504 | 0.017 |
| miR-31 | 1418598_at | Ubox5 | -0.503 | 0.017 |
| miR-31 | 1436804_s_at | Scyl1 | -0.502 | 0.017 |
| miR-31 | 1422465_a_at | Nxn | 0.502 | 0.017 |
| miR-31 | 1455138_x_at | Cfl1 | -0.502 | 0.017 |
| miR-31 | 1436884_x_at | Ewsr1 | -0.502 | 0.017 |
| miR-31 | 1435685_x_at | Abcc5 | -0.502 | 0.017 |
| miR-31 | 1432289_a_at | Jsrp1 | -0.501 | 0.018 |
| miR-31 | 1434116_at | Cbx2 | 0.500 | 0.018 |
| miR-31 | 1460554_s_at | Glg1 | 0.500 | 0.018 |
| miR-31 | 1423032_at | Rpl39 | 0.499 | 0.018 |
| miR-31 | 1453243_at | 0610030E20Rik | 0.499 | 0.018 |
| miR-31 | 1429560_at | LOC100043468 /// Zfp422-rs1 | 0.499 | 0.018 |
| miR-31 | 1418186_at | Gstt1 | -0.498 | 0.018 |
| miR-31 | 1417567_at | Ctnnbip1 | 0.498 | 0.018 |
| miR-31 | 1439165_at | NA | 0.497 | 0.019 |
| miR-31 | 1439455_x_at | Capza1 | -0.497 | 0.019 |
| miR-31 | 1424043_at | Ppil4 | -0.496 | 0.019 |
| miR-31 | 1450478_a_at | Ptpn12 | -0.496 | 0.019 |
| miR-31 | 1417386_at | Npepps | 0.496 | 0.019 |
| miR-31 | 1454662_at | A230106M15Rik | 0.496 | 0.019 |
| miR-31 | 1429162_at | 1500015A07Rik | 0.496 | 0.019 |
| miR-31 | 1435712_a_at | LOC100039740 /// LOC100042791 /// LOC100047250 /// Rps18 | -0.495 | 0.019 |
| miR-31 | 1440920_at | NA | 0.495 | 0.019 |
| miR-31 | 1415787_at | Ganab | 0.495 | 0.019 |
| miR-31 | 1419244_a_at | Rab14 | -0.494 | 0.019 |
| miR-31 | 1449039_a_at | Hnrpdl | -0.494 | 0.019 |
| miR-31 | 1435890_at | 5730596K20Rik | -0.494 | 0.020 |
| miR-31 | 1424284_at | Pomt1 | -0.493 | 0.020 |
| miR-31 | 1455744_at | NA | 0.493 | 0.020 |
| miR-31 | 1438191_a_at | Rnf40 | 0.493 | 0.020 |
| miR-31 | 1428841_at | Best1 | -0.491 | 0.020 |
| miR-31 | 1453169_a_at | Gtf2h1 | 0.491 | 0.020 |
| miR-31 | 1443314_at | 2410042D21Rik | -0.491 | 0.020 |
| miR-31 | 1454784_at | Hs3st2 | -0.491 | 0.020 |
| miR-31 | 1443966_at | NA | -0.489 | 0.021 |
| miR-31 | 1434516_at | Pstk | -0.489 | 0.021 |
| miR-31 | 1434144_s_at | 2410187C16Rik | 0.489 | 0.021 |
| miR-31 | 1455954_x_at | Gpaa1 | -0.489 | 0.021 |
| miR-31 | 1455994_x_at | Elovl1 | 0.489 | 0.021 |
| miR-31 | 1423583_at | Fem1a | -0.489 | 0.021 |
| miR-31 | 1451429_at | Gfod2 | -0.489 | 0.021 |
| miR-31 | 1445899_at | AA409289 | 0.488 | 0.021 |
| miR-31 | 1422525_at | Atp5k | 0.488 | 0.021 |
| miR-31 | 1453810_at | Aftph | -0.487 | 0.022 |
| miR-31 | 1419802_at | Ccdc12 /// LOC667210 | 0.487 | 0.022 |
| miR-31 | 1437483_at | Zfp513 | -0.486 | 0.022 |
| miR-31 | 1452686_s_at | D4Ertd196e /// LOC100046421 | -0.486 | 0.022 |
| miR-31 | 1451391_at | 2700050L05Rik | 0.486 | 0.022 |
| miR-31 | 1457425_at | C130038G02Rik | 0.485 | 0.022 |
| miR-31 | 1448615_at | Ccs | 0.485 | 0.022 |
| miR-31 | 1448527_at | Pdcd10 | -0.485 | 0.022 |
| miR-31 | 1435653_at | NA | -0.485 | 0.022 |
| miR-31 | 1429217_at | Zfp655 | -0.485 | 0.022 |
| miR-31 | 1421534_at | LOC14210 | -0.484 | 0.022 |
| miR-31 | 1416603_at | LOC632026 /// mCG_130059 /// Rpl22 | -0.484 | 0.023 |
| miR-31 | 1458975_at | NA | -0.483 | 0.023 |
| miR-31 | 1418067_at | Cfl2 | -0.483 | 0.023 |
| miR-31 | 1457529_x_at | NA | -0.483 | 0.023 |
| miR-31 | 1448549_a_at | Dpagt1 | 0.483 | 0.023 |
| miR-31 | 1435230_at | Ankrd12 /// Ankrd12-like | -0.483 | 0.023 |
| miR-31 | 1444539_at | D530039A21Rik | -0.483 | 0.023 |
| miR-31 | 1444552_at | NA | 0.483 | 0.023 |
| miR-31 | 1435744_at | 6720401G13Rik | 0.483 | 0.023 |
| miR-31 | 1433677_at | Sfrs8 | -0.483 | 0.023 |
| miR-31 | 1448399_at | Tax1bp1 | -0.482 | 0.023 |
| miR-31 | 1424416_at | Nkiras2 | -0.482 | 0.023 |
| miR-31 | 1442297_at | BB212172 | 0.482 | 0.023 |
| miR-31 | 1430937_at | A930016O22Rik | -0.481 | 0.023 |
| miR-31 | 1458843_at | NA | -0.481 | 0.023 |
| miR-31 | 1417833_at | Zc3h10 | -0.480 | 0.024 |
| miR-31 | 1434078_at | Ubfd1 | 0.480 | 0.024 |
| miR-31 | 1443968_at | Adarb1 | 0.479 | 0.024 |
| miR-31 | 1436991_x_at | Gsn | -0.479 | 0.024 |
| miR-31 | 1427956_at | Pcgf1 | -0.479 | 0.024 |
| miR-31 | 1456021_at | Atf6 | 0.478 | 0.024 |
| miR-31 | 1458204_at | Sparc | 0.478 | 0.024 |
| miR-31 | 1452189_at | Wdr82 | -0.478 | 0.025 |
| miR-31 | 1433936_at | 0610010E21Rik | -0.478 | 0.025 |
| miR-31 | 1449047_at | Hacl1 | -0.477 | 0.025 |
| miR-31 | 1436214_at | 1110028C15Rik | -0.477 | 0.025 |
| miR-31 | 1435690_at | 2310008H09Rik | 0.477 | 0.025 |
| miR-31 | 1448817_at | LOC100046081 /// Otub1 | 0.477 | 0.025 |
| miR-31 | 1431874_at | 4931429L15Rik | 0.476 | 0.025 |
| miR-31 | 1449970_at | Capn12 | 0.476 | 0.025 |
| miR-31 | 1428335_a_at | Scfd1 | -0.476 | 0.025 |
| miR-31 | 1428576_at | Hif1an | 0.476 | 0.025 |
| miR-31 | 1454929_s_at | Safb | 0.476 | 0.025 |
| miR-31 | 1418127_a_at | Aifm1 | -0.476 | 0.025 |
| miR-31 | 1450739_at | Tbl1xr1 | 0.476 | 0.025 |
| miR-31 | 1437165_a_at | Pcolce | -0.475 | 0.025 |
| miR-31 | 1424519_at | Mtg1 | 0.475 | 0.025 |
| miR-31 | 1424914_at | 2310044G17Rik | 0.475 | 0.025 |
| miR-31 | 1416215_at | Gosr1 | 0.475 | 0.025 |
| miR-31 | 1441296_at | NA | -0.475 | 0.026 |
| miR-31 | 1426402_at | Syncrip | -0.474 | 0.026 |
| miR-31 | 1456275_at | Mrpl21 | -0.474 | 0.026 |
| miR-31 | 1435398_at | Stxbp5 | 0.474 | 0.026 |
| miR-31 | 1421878_at | Mapk9 | -0.474 | 0.026 |
| miR-31 | 1417233_at | Chchd4 | 0.474 | 0.026 |
| miR-31 | 1452069_a_at | Thap7 | -0.474 | 0.026 |
| miR-31 | 1419359_at | Hexim1 | -0.474 | 0.026 |
| miR-31 | 1416466_at | Vapa | -0.473 | 0.026 |
| miR-31 | 1437670_x_at | Cd151 | -0.473 | 0.026 |
| miR-31 | 1435908_at | Nrxn2 | -0.473 | 0.026 |
| miR-31 | 1428505_at | Ccdc90b | -0.473 | 0.026 |
| miR-31 | 1443726_at | Smyd1 | 0.473 | 0.026 |
| miR-31 | 1437557_at | D3Ertd254e | 0.472 | 0.026 |
| miR-31 | 1450186_s_at | Gnas | -0.472 | 0.027 |
| miR-31 | 1451459_at | Ahctf1 | 0.472 | 0.027 |
| miR-31 | 1456424_s_at | Pltp | -0.472 | 0.027 |
| miR-31 | 1454820_at | BC037034 | 0.471 | 0.027 |
| miR-31 | 1434995_s_at | Dedd | -0.471 | 0.027 |
| miR-31 | 1434266_at | AI847670 | 0.471 | 0.027 |
| miR-31 | 1421047_at | LOC100046891 /// Smad5 | 0.471 | 0.027 |
| miR-31 | 1416476_a_at | Ube2d2 | -0.470 | 0.027 |
| miR-31 | 1446613_at | NA | 0.470 | 0.027 |
| miR-31 | 1421160_a_at | Rfng | -0.470 | 0.027 |
| miR-31 | 1440228_at | Ranbp6 | 0.470 | 0.027 |
| miR-31 | 1451218_at | Edem1 | 0.470 | 0.027 |
| miR-31 | 1447233_at | D330027H18Rik | -0.470 | 0.027 |
| miR-31 | 1456010_x_at | Hes5 | 0.470 | 0.027 |
| miR-31 | 1444448_at | 1300007F04Rik | 0.469 | 0.028 |
| miR-31 | 1428128_at | 4921506J03Rik | 0.469 | 0.028 |
| miR-31 | 1423163_at | Bat4 | 0.469 | 0.028 |
| miR-31 | 1456085_x_at | Cd151 | -0.469 | 0.028 |
| miR-31 | 1452271_at | Xpr1 | 0.469 | 0.028 |
| miR-31 | 1434960_at | Taf9b | -0.469 | 0.028 |
| miR-31 | 1431390_a_at | Grinl1a | 0.469 | 0.028 |
| miR-31 | 1436946_s_at | Gng5 /// LOC100041120 /// LOC100043507 /// LOC100044719 /// LOC100045733 /// LOC100045948 /// LOC100047170 /// LOC100048410 | -0.468 | 0.028 |
| miR-31 | 1423208_at | Tmem167 | 0.468 | 0.028 |
| miR-31 | 1420809_a_at | 1500003O03Rik /// LOC100048622 | -0.468 | 0.028 |
| miR-31 | 1416475_at | LOC100041725 /// Ube2d2 | -0.468 | 0.028 |
| miR-31 | 1448106_at | Necap1 | 0.468 | 0.028 |
| miR-31 | 1419503_at | Stc2 | -0.467 | 0.028 |
| miR-31 | 1438236_at | Nfia | 0.467 | 0.028 |
| miR-31 | 1417035_at | Sac3d1 | -0.467 | 0.029 |
| miR-31 | 1428425_at | Tgfbrap1 | 0.466 | 0.029 |
| miR-31 | 1423845_at | Csdc2 | -0.466 | 0.029 |
| miR-31 | 1416513_at | Lamb2 | -0.466 | 0.029 |
| miR-31 | 1434680_at | Plekhg3 | 0.466 | 0.029 |
| miR-31 | 1416703_at | Mapk14 | -0.466 | 0.029 |
| miR-31 | 1429026_at | Hexim2 /// LOC100044959 | -0.466 | 0.029 |
| miR-31 | 1418886_s_at | Idh3b | -0.466 | 0.029 |
| miR-31 | 1437666_x_at | Gm1821 /// LOC100048105 /// Rps27a /// Ubb /// Ubc | -0.465 | 0.029 |
| miR-31 | 1459323_at | NA | 0.465 | 0.029 |
| miR-31 | 1454863_at | Ankrd11 | -0.465 | 0.029 |
| miR-31 | 1450965_at | Tex261 | 0.465 | 0.029 |
| miR-31 | 1422119_at | Rab5b | -0.465 | 0.029 |
| miR-31 | 1435140_at | Ide | 0.464 | 0.029 |
| miR-31 | 1428865_at | Bcl2l12 | 0.464 | 0.030 |
| miR-31 | 1454807_a_at | Snx12 | 0.464 | 0.030 |
| miR-31 | 1435441_at | Ablim2 | -0.464 | 0.030 |
| miR-31 | 1428241_at | 2310035K24Rik | 0.464 | 0.030 |
| miR-31 | 1454871_at | Rbm15b | 0.464 | 0.030 |
| miR-31 | 1455026_at | Sbno1 | 0.464 | 0.030 |
| miR-31 | 1443569_at | 4930430E16Rik | -0.463 | 0.030 |
| miR-31 | 1442319_at | Usp4 | 0.463 | 0.030 |
| miR-31 | 1418306_at | Crybb1 | -0.463 | 0.030 |
| miR-31 | 1423624_at | Fancl /// LOC100044333 | -0.463 | 0.030 |
| miR-31 | 1452018_at | Nkx2-6 | -0.463 | 0.030 |
| miR-31 | 1432275_at | Ikbkb | 0.462 | 0.030 |
| miR-31 | 1416653_at | LOC100047484 /// Stxbp3a | -0.462 | 0.030 |
| miR-31 | 1437942_x_at | Tube1 | 0.462 | 0.030 |
| miR-31 | 1417239_at | Cetn3 | -0.462 | 0.030 |
| miR-31 | 1449255_a_at | Rpl15 | -0.462 | 0.030 |
| miR-31 | 1415899_at | Junb | 0.462 | 0.031 |
| miR-31 | 1426071_at | Tiaf2 | 0.461 | 0.031 |
| miR-31 | 1446614_at | Dgkz | 0.461 | 0.031 |
| miR-31 | 1424999_at | 1700022C21Rik | -0.461 | 0.031 |
| miR-31 | 1438181_x_at | Tm2d2 | -0.461 | 0.031 |
| miR-31 | 1427248_at | Whsc2 | -0.461 | 0.031 |
| miR-31 | 1415823_at | Scd2 | 0.460 | 0.031 |
| miR-31 | 1437910_at | Tmem39b | -0.460 | 0.031 |
| miR-31 | 1450351_a_at | Clip1 | -0.460 | 0.031 |
| miR-31 | 1438601_at | Pkmyt1 | 0.460 | 0.031 |
| miR-31 | 1431877_a_at | Grhl2 | -0.460 | 0.031 |
| miR-31 | 1450900_at | Smek2 | -0.460 | 0.031 |
| miR-31 | 1437144_x_at | Psma6 | -0.459 | 0.032 |
| miR-31 | 1420093_s_at | Hnrpdl | -0.459 | 0.032 |
| miR-31 | 1448903_at | sept15 | -0.459 | 0.032 |
| miR-31 | 1452584_at | 1500032L24Rik | 0.459 | 0.032 |
| miR-31 | 1450368_a_at | Ppp3r1 | -0.458 | 0.032 |
| miR-31 | 1426680_at | Sepn1 | -0.458 | 0.032 |
| miR-31 | 1456393_at | 2310002J21Rik | -0.458 | 0.032 |
| miR-31 | 1438315_x_at | Akr7a5 | 0.457 | 0.032 |
| miR-31 | 1427002_s_at | Arsg | 0.457 | 0.032 |
| miR-31 | 1457379_at | C78549 | -0.457 | 0.032 |
| miR-31 | 1450662_at | Tesk1 | -0.457 | 0.032 |
| miR-31 | 1460594_a_at | Gmppa | -0.457 | 0.033 |
| miR-31 | 1435619_at | Phf21a | 0.457 | 0.033 |
| miR-31 | 1426859_at | Inhbb /// LOC100046802 | 0.456 | 0.033 |
| miR-31 | 1437172_x_at | Hadhb | -0.456 | 0.033 |
| miR-31 | 1449420_at | Pde1b | 0.456 | 0.033 |
| miR-31 | 1455897_x_at | Hmgn1 /// LOC100044391 | -0.455 | 0.033 |
| miR-31 | 1436427_at | Prpf4b | 0.455 | 0.033 |
| miR-31 | 1433100_at | Lmf2 | -0.455 | 0.033 |
| miR-31 | 1436537_at | Zfp629 | 0.455 | 0.033 |
| miR-31 | 1438174_x_at | Ppp2r1a | 0.455 | 0.034 |
| miR-31 | 1417646_a_at | Snx5 | -0.454 | 0.034 |
| miR-31 | 1452596_at | Polr2k | -0.454 | 0.034 |
| miR-31 | 1457181_at | D15Wsu75e | 0.454 | 0.034 |
| miR-31 | 1438683_at | Wasf2 | 0.454 | 0.034 |
| miR-31 | 1427881_at | Dnttip2 | -0.454 | 0.034 |
| miR-31 | 1424104_at | Syf2 | 0.454 | 0.034 |
| miR-31 | 1452060_a_at | Limk2 | 0.454 | 0.034 |
| miR-31 | 1448383_at | Mmp14 | 0.454 | 0.034 |
| miR-31 | 1419663_at | Ogn | -0.454 | 0.034 |
| miR-31 | 1435645_at | LOC676546 /// Mmd | 0.453 | 0.034 |
| miR-31 | 1444131_at | NA | 0.453 | 0.034 |
| miR-31 | 1437938_x_at | Dnm2 | -0.453 | 0.034 |
| miR-31 | 1418885_a_at | Idh3b | -0.453 | 0.034 |
| miR-31 | 1424267_at | 1810043G02Rik | -0.453 | 0.034 |
| miR-31 | 1422522_at | Fxr2 | 0.453 | 0.034 |
| miR-31 | 1451612_at | Mt1 | -0.453 | 0.034 |
| miR-31 | 1450425_a_at | 2700062C07Rik | -0.453 | 0.034 |
| miR-31 | 1435380_at | Cox10 | 0.453 | 0.034 |
| miR-31 | 1418052_at | Mvk | -0.453 | 0.034 |
| miR-31 | 1438940_x_at | Hmgn1 /// LOC100040836 /// LOC100044391 /// LOC665957 /// LOC666721 | -0.452 | 0.034 |
| miR-31 | 1424112_at | Igf2r | 0.452 | 0.035 |
| miR-31 | 1427972_at | Cdc73 | 0.452 | 0.035 |
| miR-31 | 1427159_at | Pcf11 | -0.452 | 0.035 |
| miR-31 | 1457706_at | BC030336 | 0.452 | 0.035 |
| miR-31 | 1430005_a_at | Batf2 | -0.452 | 0.035 |
| miR-31 | 1450394_at | Golph3 | -0.452 | 0.035 |
| miR-31 | 1444001_at | NA | -0.452 | 0.035 |
| miR-31 | 1447226_at | NA | 0.451 | 0.035 |
| miR-31 | 1417794_at | Zmym3 | 0.451 | 0.035 |
| miR-31 | 1426873_s_at | Jup | -0.451 | 0.035 |
| miR-31 | 1428258_at | 2010107E04Rik | 0.451 | 0.035 |
| miR-31 | 1451727_at | Slu7 | -0.450 | 0.035 |
| miR-31 | 1436834_x_at | Mdh1 | -0.450 | 0.036 |
| miR-31 | 1416731_at | Top2b | -0.450 | 0.036 |
| miR-31 | 1432735_at | 1700017H01Rik | 0.450 | 0.036 |
| miR-31 | 1456800_a_at | Rltpr | 0.450 | 0.036 |
| miR-31 | 1456244_x_at | Glrx3 | -0.450 | 0.036 |
| miR-31 | 1417864_at | Pgk1 | 0.449 | 0.036 |
| miR-31 | 1429370_a_at | Psmd11 | -0.449 | 0.036 |
| miR-31 | 1458028_at | 1810012P15Rik | 0.449 | 0.036 |
| miR-31 | 1459788_at | Gpr107 | -0.449 | 0.036 |
| miR-31 | 1453206_at | Acad9 | 0.449 | 0.036 |
| miR-31 | 1440928_at | D630037F22Rik | 0.449 | 0.036 |
| miR-31 | 1419136_at | Akr1c18 | 0.449 | 0.036 |
| miR-31 | 1444180_at | NA | 0.448 | 0.036 |
| miR-31 | 1457265_at | Sfrs17b | 0.448 | 0.036 |
| miR-31 | 1429914_at | Epc1 | 0.447 | 0.037 |
| miR-31 | 1429238_a_at | Ogfod2 | 0.447 | 0.037 |
| miR-31 | 1447949_at | LOC100041228 /// Zfp787 | -0.447 | 0.037 |
| miR-31 | 1436796_at | Matr3 | -0.447 | 0.037 |
| miR-31 | 1430327_at | mCG_13386 | -0.447 | 0.037 |
| miR-31 | 1446070_at | Tyw1 | 0.446 | 0.037 |
| miR-31 | 1427907_at | 1110037F02Rik | -0.446 | 0.037 |
| miR-31 | 1423336_at | Orc4l | -0.446 | 0.037 |
| miR-31 | 1440384_at | Tmcc1 | 0.446 | 0.037 |
| miR-31 | 1423930_at | Anapc4 | -0.446 | 0.037 |
| miR-31 | 1415683_at | Nmt1 | -0.446 | 0.037 |
| miR-31 | 1448384_at | Pofut2 | -0.446 | 0.038 |
| miR-31 | 1436383_at | Cplx2 | -0.446 | 0.038 |
| miR-31 | 1418168_at | Zcchc14 | -0.446 | 0.038 |
| miR-31 | 1424619_at | Sf3b4 | -0.446 | 0.038 |
| miR-31 | 1456698_s_at | Hnrpdl | -0.445 | 0.038 |
| miR-31 | 1447349_s_at | Ep400 | 0.445 | 0.038 |
| miR-31 | 1430418_at | Tmem57 | -0.445 | 0.038 |
| miR-31 | 1438195_at | Gpd1l | 0.445 | 0.038 |
| miR-31 | 1454922_at | Wdr92 | 0.445 | 0.038 |
| miR-31 | 1459476_s_at | Csrp2bp /// LOC100048645 | -0.444 | 0.038 |
| miR-31 | 1438163_x_at | Rhbdd2 | 0.444 | 0.038 |
| miR-31 | 1420171_s_at | Myh9 | -0.444 | 0.038 |
| miR-31 | 1432830_at | Ccdc138 | 0.444 | 0.038 |
| miR-31 | 1438620_x_at | Sfrp1 | 0.444 | 0.038 |
| miR-31 | 1428405_at | Hcfc1r1 | -0.444 | 0.038 |
| miR-31 | 1441200_at | Klf3 | 0.444 | 0.039 |
| miR-31 | 1438416_at | Med16 | -0.443 | 0.039 |
| miR-31 | 1423712_a_at | Qars | -0.443 | 0.039 |
| miR-31 | 1454272_at | 9630015K15Rik | -0.443 | 0.039 |
| miR-31 | 1433874_at | Ssh1 | 0.443 | 0.039 |
| miR-31 | 1448154_at | Ndrg2 | 0.443 | 0.039 |
| miR-31 | 1423570_at | Abcg1 | 0.442 | 0.039 |
| miR-31 | 1423797_at | Aacs | 0.442 | 0.039 |
| miR-31 | 1450149_a_at | Ppp1cc | -0.442 | 0.039 |
| miR-31 | 1438019_at | Ippk | 0.442 | 0.039 |
| miR-31 | 1449566_at | Nkx2-5 | -0.442 | 0.039 |
| miR-31 | 1453321_at | Fndc1 | -0.442 | 0.039 |
| miR-31 | 1417271_a_at | Eng | -0.442 | 0.040 |
| miR-31 | 1421918_at | Anp32a | 0.442 | 0.040 |
| miR-31 | 1434503_s_at | Lamp2 | -0.442 | 0.040 |
| miR-31 | 1427476_a_at | Trim32 | -0.442 | 0.040 |
| miR-31 | 1419224_at | Cecr6 | 0.442 | 0.040 |
| miR-31 | 1451471_at | Ears2 | 0.442 | 0.040 |
| miR-31 | 1426312_at | Bre | -0.441 | 0.040 |
| miR-31 | 1418760_at | Rdh11 | -0.441 | 0.040 |
| miR-31 | 1451084_at | Etfdh | -0.441 | 0.040 |
| miR-31 | 1426136_x_at | Klra21 | 0.441 | 0.040 |
| miR-31 | 1445560_at | NA | 0.441 | 0.040 |
| miR-31 | 1439444_x_at | Tmed10 | -0.441 | 0.040 |
| miR-31 | 1415814_at | Atp6v1b2 | 0.441 | 0.040 |
| miR-31 | 1434963_at | Supt3h | -0.441 | 0.040 |
| miR-31 | 1436151_x_at | BC031781 | 0.441 | 0.040 |
| miR-31 | 1415774_at | Elp2 | -0.440 | 0.040 |
| miR-31 | 1432543_a_at | Klf13 | -0.440 | 0.040 |
| miR-31 | 1439374_x_at | Rps10 | -0.440 | 0.040 |
| miR-31 | 1439433_a_at | Slc35a2 | -0.440 | 0.040 |
| miR-31 | 1452181_at | Ckap4 | 0.440 | 0.041 |
| miR-31 | 1438028_at | 4930535B03Rik | 0.440 | 0.041 |
| miR-31 | 1419384_at | Pick1 | -0.440 | 0.041 |
| miR-31 | 1439650_at | Rtn4 | 0.440 | 0.041 |
| miR-31 | 1434665_at | Aga | -0.440 | 0.041 |
| miR-31 | 1417152_at | Btbd14a | 0.439 | 0.041 |
| miR-31 | 1415739_at | Rbm42 | -0.439 | 0.041 |
| miR-31 | 1415922_s_at | Marcksl1 | 0.439 | 0.041 |
| miR-31 | 1439497_at | NA | -0.439 | 0.041 |
| miR-31 | 1460564_at | Zfp280b | 0.439 | 0.041 |
| miR-31 | 1444585_at | Adc | 0.439 | 0.041 |
| miR-31 | 1441922_x_at | Vps11 | 0.438 | 0.041 |
| miR-31 | 1426995_a_at | Gfer | -0.438 | 0.041 |
| miR-31 | 1426421_s_at | Rbm26 | 0.438 | 0.041 |
| miR-31 | 1416321_s_at | Prelp | -0.438 | 0.041 |
| miR-31 | 1447467_at | Ptpra | 0.438 | 0.042 |
| miR-31 | 1436798_at | NA | 0.438 | 0.042 |
| miR-31 | 1428917_at | Stx17 | -0.437 | 0.042 |
| miR-31 | 1435609_at | Trp53bp1 | 0.437 | 0.042 |
| miR-31 | 1455288_at | 1110036O03Rik | -0.437 | 0.042 |
| miR-31 | 1455105_at | Ptpn12 | -0.437 | 0.042 |
| miR-31 | 1453761_at | Phf6 | 0.437 | 0.042 |
| miR-31 | 1423472_at | sept2 | 0.437 | 0.042 |
| miR-31 | 1421477_at | Cplx2 | 0.437 | 0.042 |
| miR-31 | 1450092_at | Ighmbp2 | 0.437 | 0.042 |
| miR-31 | 1435948_at | LOC100040525 /// Tmem181 | 0.436 | 0.042 |
| miR-31 | 1460462_at | Med18 | 0.436 | 0.042 |
| miR-31 | 1457644_s_at | Cxcl1 | 0.436 | 0.043 |
| miR-31 | 1460536_at | 2310079F09Rik | 0.436 | 0.043 |
| miR-31 | 1423535_at | LOC100047794 /// Strn3 | -0.436 | 0.043 |
| miR-31 | 1434938_at | Rbm9 | 0.436 | 0.043 |
| miR-31 | 1433432_x_at | LOC672959 /// Rps12 | -0.436 | 0.043 |
| miR-31 | 1443558_s_at | BC030307 /// Nt5dc3 | 0.435 | 0.043 |
| miR-31 | 1455510_at | LOC100039483 | 0.435 | 0.043 |
| miR-31 | 1437196_x_at | LOC100039355 /// LOC100047501 /// LOC100048153 /// Rps16 | -0.435 | 0.043 |
| miR-31 | 1452196_a_at | Nckap1 | -0.435 | 0.043 |
| miR-31 | 1442663_at | Rbm12b | 0.434 | 0.043 |
| miR-31 | 1438196_at | Gpd1l | 0.434 | 0.043 |
| miR-31 | 1430413_at | Tmem29 | 0.434 | 0.044 |
| miR-31 | 1425742_a_at | Tsc22d1 | -0.434 | 0.044 |
| miR-31 | 1418449_at | Lad1 | 0.434 | 0.044 |
| miR-31 | 1435949_at | Zc3h3 | -0.434 | 0.044 |
| miR-31 | 1428858_at | Wdr70 | -0.433 | 0.044 |
| miR-31 | 1442768_at | E130112L23Rik | 0.433 | 0.044 |
| miR-31 | 1456117_at | Rrp1b | 0.433 | 0.044 |
| miR-31 | 1428598_at | Tbc1d7 | -0.433 | 0.044 |
| miR-31 | 1429421_at | Accs | 0.433 | 0.044 |
| miR-31 | 1424262_at | 2810003C17Rik | 0.433 | 0.044 |
| miR-31 | 1426577_a_at | Lin37 | -0.433 | 0.044 |
| miR-31 | 1437807_x_at | Ctnna1 | -0.432 | 0.044 |
| miR-31 | 1448433_a_at | Pcolce | -0.432 | 0.044 |
| miR-31 | 1417237_at | Pld2 | -0.432 | 0.045 |
| miR-31 | 1434475_at | Ppig | 0.432 | 0.045 |
| miR-31 | 1426757_at | Ampd2 | 0.431 | 0.045 |
| miR-31 | 1422801_at | G3bp1 | 0.431 | 0.045 |
| miR-31 | 1435969_at | Btbd12 | -0.431 | 0.045 |
| miR-31 | 1430830_at | A430105J06Rik | 0.431 | 0.045 |
| miR-31 | 1434872_x_at | LOC100040532 /// LOC100047378 /// Rpl37 | -0.431 | 0.045 |
| miR-31 | 1422323_a_at | Lbx1 | 0.431 | 0.045 |
| miR-31 | 1438644_x_at | Commd9 | -0.431 | 0.045 |
| miR-31 | 1437203_at | Cbll1 | 0.431 | 0.045 |
| miR-31 | 1439023_at | Ddx55 | 0.431 | 0.045 |
| miR-31 | 1416914_s_at | Mtvr2 | -0.430 | 0.046 |
| miR-31 | 1448189_a_at | Flii | -0.430 | 0.046 |
| miR-31 | 1446650_at | NA | -0.430 | 0.046 |
| miR-31 | 1455809_x_at | Ric8 | -0.429 | 0.046 |
| miR-31 | 1427935_at | Lyrm2 | 0.429 | 0.046 |
| miR-31 | 1448829_at | Smc6 | -0.429 | 0.046 |
| miR-31 | 1434406_at | Srgap2 | 0.429 | 0.046 |
| miR-31 | 1416221_at | Fstl1 | -0.429 | 0.046 |
| miR-31 | 1437649_x_at | Ppib | -0.429 | 0.046 |
| miR-31 | 1443838_x_at | Fads2 | -0.429 | 0.046 |
| miR-31 | 1455868_a_at | Tubgcp2 | -0.429 | 0.046 |
| miR-31 | 1417593_at | Tusc2 | 0.429 | 0.046 |
| miR-31 | 1440836_at | Setd1b | 0.429 | 0.046 |
| miR-31 | 1452268_at | 2810485I05Rik | -0.429 | 0.047 |
| miR-31 | 1426659_a_at | LOC100041163 /// LOC100041478 /// LOC100043755 /// LOC100046821 /// Rpl23a | -0.429 | 0.047 |
| miR-31 | 1419806_at | Hdlbp | 0.429 | 0.047 |
| miR-31 | 1428307_at | Zdhhc13 | -0.428 | 0.047 |
| miR-31 | 1450913_at | B4galt6 /// LOC675709 | 0.428 | 0.047 |
| miR-31 | 1460428_at | Ankrd13a | -0.427 | 0.047 |
| miR-31 | 1451778_at | Crtc3 /// LOC100047733 | 0.427 | 0.047 |
| miR-31 | 1437379_x_at | Trap1 | -0.427 | 0.048 |
| miR-31 | 1439623_at | NA | -0.427 | 0.048 |
| miR-31 | 1441404_at | NA | 0.426 | 0.048 |
| miR-31 | 1456726_x_at | Qars | -0.426 | 0.048 |
| miR-31 | 1438640_x_at | Pgk1 | 0.426 | 0.048 |
| miR-31 | 1441871_at | 1810044D09Rik | 0.425 | 0.048 |
| miR-31 | 1419984_s_at | Zfp644 | -0.425 | 0.049 |
| miR-31 | 1456407_a_at | LOC100046241 /// Tlk1 | -0.425 | 0.049 |
| miR-31 | 1424844_at | Angel1 | 0.425 | 0.049 |
| miR-31 | 1447902_at | 1810013A23Rik | 0.424 | 0.049 |
| miR-31 | 1429066_at | 4930565B19Rik | -0.424 | 0.049 |
| miR-31 | 1417857_at | Mmaa | 0.424 | 0.049 |
| miR-31 | 1416353_at | Nr1h2 | -0.424 | 0.049 |
| miR-31 | 1439154_at | Nup98 | -0.423 | 0.050 |
| miR-31 | 1442064_at | AW556556 | -0.423 | 0.050 |
| miR-31 | 1455562_at | Sox12 | -0.423 | 0.050 |
| miR-31 | 1420482_at | Cnnm3 | 0.423 | 0.050 |
| miR-31 | 1416984_at | Mrps18a | -0.423 | 0.050 |
| miR-34c | 1447836_x_at | 4921525O09Rik | -0.685 | 0.000 |
| miR-34c | 1429610_a_at | Zfp511 | -0.663 | 0.001 |
| miR-34c | 1418704_at | S100a13 | 0.644 | 0.001 |
| miR-34c | 1424283_at | Jtb | 0.640 | 0.001 |
| miR-34c | 1448208_at | Smad1 | 0.634 | 0.002 |
| miR-34c | 1427980_at | 4933407C03Rik | -0.631 | 0.002 |
| miR-34c | 1451405_at | Pcca | 0.628 | 0.002 |
| miR-34c | 1424699_at | Ccdc136 | -0.627 | 0.002 |
| miR-34c | 1419315_at | Slamf9 | 0.626 | 0.002 |
| miR-34c | 1417999_at | Itm2b | 0.617 | 0.002 |
| miR-34c | 1428728_at | Ddx51 | -0.616 | 0.002 |
| miR-34c | 1457707_at | Mctp2 | 0.615 | 0.002 |
| miR-34c | 1420596_at | Cacng2 | -0.613 | 0.002 |
| miR-34c | 1460024_at | Tnrc6b | -0.612 | 0.002 |
| miR-34c | 1439493_at | Zfp827 | 0.609 | 0.003 |
| miR-34c | 1417805_at | Xpnpep2 | -0.607 | 0.003 |
| miR-34c | 1422433_s_at | Idh1 | 0.607 | 0.003 |
| miR-34c | 1426483_at | Prkrir | 0.606 | 0.003 |
| miR-34c | 1456135_s_at | Pxn | 0.604 | 0.003 |
| miR-34c | 1430937_at | A930016O22Rik | -0.600 | 0.003 |
| miR-34c | 1437802_x_at | Morf4l1 | 0.598 | 0.003 |
| miR-34c | 1455249_at | NA | 0.597 | 0.003 |
| miR-34c | 1426700_a_at | Usp52 | -0.595 | 0.004 |
| miR-34c | 1417987_at | Btd | 0.594 | 0.004 |
| miR-34c | 1451226_at | Pex6 | -0.594 | 0.004 |
| miR-34c | 1460116_s_at | Spred1 | 0.590 | 0.004 |
| miR-34c | 1448875_at | Zhx1 | 0.589 | 0.004 |
| miR-34c | 1436945_x_at | LOC100045432 /// Stim1 | -0.588 | 0.004 |
| miR-34c | 1433749_at | Gna13 | 0.586 | 0.004 |
| miR-34c | 1424536_at | Oas1e | -0.585 | 0.004 |
| miR-34c | 1455143_at | Nlgn2 | -0.584 | 0.004 |
| miR-34c | 1427065_at | 4933439F18Rik | -0.583 | 0.004 |
| miR-34c | 1440613_at | NA | -0.576 | 0.005 |
| miR-34c | 1426524_at | Gnpda2 | 0.575 | 0.005 |
| miR-34c | 1430327_at | mCG_13386 | -0.575 | 0.005 |
| miR-34c | 1447711_x_at | 4933412E12Rik | 0.574 | 0.005 |
| miR-34c | 1438595_at | Gm546 | -0.574 | 0.005 |
| miR-34c | 1453365_at | Rabgap1l | 0.570 | 0.006 |
| miR-34c | 1453999_at | 4921511H13Rik | -0.566 | 0.006 |
| miR-34c | 1456567_x_at | Grn | 0.565 | 0.006 |
| miR-34c | 1420000_s_at | Igbp1 | 0.564 | 0.006 |
| miR-34c | 1418029_at | Faim | 0.562 | 0.007 |
| miR-34c | 1426316_at | 6330416G13Rik | 0.561 | 0.007 |
| miR-34c | 1451310_a_at | Ctsl | 0.560 | 0.007 |
| miR-34c | 1460432_a_at | Eif3e | 0.559 | 0.007 |
| miR-34c | 1451274_at | Ogdh | -0.559 | 0.007 |
| miR-34c | 1417358_s_at | Sorbs1 | -0.558 | 0.007 |
| miR-34c | 1421043_s_at | Arhgef2 | 0.557 | 0.007 |
| miR-34c | 1416629_at | Slc1a5 | -0.555 | 0.007 |
| miR-34c | 1433837_at | 8430408G22Rik | -0.554 | 0.007 |
| miR-34c | 1435260_at | Akt3 | 0.551 | 0.008 |
| miR-34c | 1416452_at | Oat | 0.549 | 0.008 |
| miR-34c | 1437494_at | Mapkapk3 | 0.546 | 0.009 |
| miR-34c | 1418319_at | Ufsp2 | 0.543 | 0.009 |
| miR-34c | 1435822_at | D830012I24Rik | 0.543 | 0.009 |
| miR-34c | 1424817_at | Spef1 | -0.543 | 0.009 |
| miR-34c | 1448388_a_at | 1110002B05Rik | 0.543 | 0.009 |
| miR-34c | 1439380_x_at | Meg3 | -0.541 | 0.009 |
| miR-34c | 1420397_a_at | Spen | -0.539 | 0.010 |
| miR-34c | 1423596_at | Nek6 | 0.536 | 0.010 |
| miR-34c | 1457952_at | NA | 0.536 | 0.010 |
| miR-34c | 1420819_at | Sla | 0.536 | 0.010 |
| miR-34c | 1428222_at | Dclk2 | -0.535 | 0.010 |
| miR-34c | 1424656_s_at | Usp19 | -0.535 | 0.010 |
| miR-34c | 1439548_at | Rap2b | 0.534 | 0.010 |
| miR-34c | 1448308_at | Ap3m1 | 0.531 | 0.011 |
| miR-34c | 1436806_at | Trim62 | -0.531 | 0.011 |
| miR-34c | 1444654_at | NA | -0.531 | 0.011 |
| miR-34c | 1433460_at | Ttc7b | 0.528 | 0.012 |
| miR-34c | 1448206_at | Psma2 | 0.527 | 0.012 |
| miR-34c | 1424038_a_at | 2310044H10Rik | -0.526 | 0.012 |
| miR-34c | 1432111_at | 4930542D17Rik | -0.525 | 0.012 |
| miR-34c | 1425492_at | Bmpr1a | 0.524 | 0.012 |
| miR-34c | 1434754_at | Garnl4 | 0.524 | 0.012 |
| miR-34c | 1424210_at | Erlin1 | 0.523 | 0.013 |
| miR-34c | 1439529_at | A430110N23Rik | -0.523 | 0.013 |
| miR-34c | 1423699_at | Ncaph2 | -0.523 | 0.013 |
| miR-34c | 1438034_at | 2410005O16Rik | 0.523 | 0.013 |
| miR-34c | 1444842_at | Jarid1a | 0.522 | 0.013 |
| miR-34c | 1418521_a_at | Mtx1 | 0.521 | 0.013 |
| miR-34c | 1454896_at | Rbpj | 0.521 | 0.013 |
| miR-34c | 1434568_at | NA | 0.519 | 0.013 |
| miR-34c | 1432910_at | Btbd7 | 0.518 | 0.014 |
| miR-34c | 1424567_at | Tspan2 | 0.517 | 0.014 |
| miR-34c | 1447302_at | NA | -0.517 | 0.014 |
| miR-34c | 1419096_at | Apom | -0.517 | 0.014 |
| miR-34c | 1456839_at | NA | 0.516 | 0.014 |
| miR-34c | 1454112_a_at | Cep27 | 0.516 | 0.014 |
| miR-34c | 1423073_at | Cmpk1 | 0.516 | 0.014 |
| miR-34c | 1455450_at | Ptpn3 | 0.515 | 0.014 |
| miR-34c | 1428470_at | Exoc2 | 0.515 | 0.014 |
| miR-34c | 1437345_a_at | Bscl2 | 0.514 | 0.014 |
| miR-34c | 1460695_a_at | 2010111I01Rik | -0.514 | 0.014 |
| miR-34c | 1435685_x_at | Abcc5 | -0.514 | 0.014 |
| miR-34c | 1437101_at | Lats2 | 0.513 | 0.015 |
| miR-34c | 1419114_at | Alg14 | -0.513 | 0.015 |
| miR-34c | 1457848_at | NA | 0.511 | 0.015 |
| miR-34c | 1426951_at | Crim1 | 0.511 | 0.015 |
| miR-34c | 1449773_s_at | Gadd45b | 0.510 | 0.015 |
| miR-34c | 1450086_at | Gmeb1 | -0.510 | 0.015 |
| miR-34c | 1455589_at | 1700100M05Rik | 0.509 | 0.016 |
| miR-34c | 1436062_at | Arcn1 | 0.509 | 0.016 |
| miR-34c | 1440268_at | Trim41 | 0.509 | 0.016 |
| miR-34c | 1418988_at | Pex7 | 0.509 | 0.016 |
| miR-34c | 1426999_at | Zc3h14 | 0.508 | 0.016 |
| miR-34c | 1418355_at | Nucb2 | 0.507 | 0.016 |
| miR-34c | 1416635_at | Smpdl3a | 0.507 | 0.016 |
| miR-34c | 1434066_at | Gtf3c1 | -0.506 | 0.016 |
| miR-34c | 1423208_at | Tmem167 | 0.504 | 0.017 |
| miR-34c | 1441948_x_at | Zfand3 | 0.503 | 0.017 |
| miR-34c | 1415856_at | Emb | 0.503 | 0.017 |
| miR-34c | 1454923_at | Iws1 | 0.503 | 0.017 |
| miR-34c | 1426383_at | Cry2 /// LOC100048334 | -0.502 | 0.017 |
| miR-34c | 1419876_at | 2810449G22Rik | -0.501 | 0.017 |
| miR-34c | 1428334_at | Ostm1 | 0.501 | 0.018 |
| miR-34c | 1450691_at | Caskin2 | -0.501 | 0.018 |
| miR-34c | 1449211_at | Bpnt1 | 0.499 | 0.018 |
| miR-34c | 1435645_at | LOC676546 /// Mmd | 0.499 | 0.018 |
| miR-34c | 1444323_at | Ccnd3 | -0.499 | 0.018 |
| miR-34c | 1433713_at | Gcn1l1 | -0.499 | 0.018 |
| miR-34c | 1437741_at | Rab21 | 0.498 | 0.018 |
| miR-34c | 1416279_at | Ap1b1 | -0.498 | 0.018 |
| miR-34c | 1433686_at | Cabin1 | -0.498 | 0.018 |
| miR-34c | 1428371_at | Ttbk2 | -0.497 | 0.019 |
| miR-34c | 1419005_at | Crybb3 | -0.497 | 0.019 |
| miR-34c | 1451904_a_at | Adam33 | -0.495 | 0.019 |
| miR-34c | 1455514_at | Kcnd1 | -0.495 | 0.019 |
| miR-34c | 1456010_x_at | Hes5 | 0.494 | 0.019 |
| miR-34c | 1435875_at | Prkab2 | 0.494 | 0.019 |
| miR-34c | 1434764_at | Akap11 | 0.493 | 0.020 |
| miR-34c | 1435350_at | Traf6 | 0.493 | 0.020 |
| miR-34c | 1416370_at | Zscan21 | 0.492 | 0.020 |
| miR-34c | 1439314_at | Clock | 0.492 | 0.020 |
| miR-34c | 1455274_at | NA | -0.491 | 0.020 |
| miR-34c | 1428700_at | P2ry13 | 0.491 | 0.020 |
| miR-34c | 1431766_x_at | Rps2 | 0.490 | 0.020 |
| miR-34c | 1419169_at | Mapk6 | 0.490 | 0.021 |
| miR-34c | 1427153_at | Bckdhb | 0.490 | 0.021 |
| miR-34c | 1416381_a_at | Prdx5 | 0.490 | 0.021 |
| miR-34c | 1437951_at | Dis3l2 | 0.490 | 0.021 |
| miR-34c | 1431055_a_at | Snx10 | 0.490 | 0.021 |
| miR-34c | 1444089_at | Spnb2 | 0.489 | 0.021 |
| miR-34c | 1434010_at | Als2cr13 | 0.489 | 0.021 |
| miR-34c | 1433593_at | Ypel5 | 0.489 | 0.021 |
| miR-34c | 1449263_at | Ufm1 | 0.489 | 0.021 |
| miR-34c | 1435450_at | Cpne3 | 0.488 | 0.021 |
| miR-34c | 1419687_at | Macrod1 | 0.487 | 0.022 |
| miR-34c | 1416536_at | Mum1 | -0.487 | 0.022 |
| miR-34c | 1416116_at | Orc3l | 0.487 | 0.022 |
| miR-34c | 1448765_at | Fyn | 0.487 | 0.022 |
| miR-34c | 1416261_at | Tmem19 | 0.486 | 0.022 |
| miR-34c | 1423758_at | G3bp2 | 0.486 | 0.022 |
| miR-34c | 1436333_a_at | Synj1 | -0.485 | 0.022 |
| miR-34c | 1456022_at | Hipk2 | -0.485 | 0.022 |
| miR-34c | 1454843_at | Prps2 | 0.485 | 0.022 |
| miR-34c | 1452464_a_at | Metapl1 | -0.485 | 0.022 |
| miR-34c | 1418259_a_at | Entpd2 | 0.485 | 0.022 |
| miR-34c | 1429389_at | Setmar | 0.485 | 0.022 |
| miR-34c | 1416080_at | Adam15 | -0.484 | 0.022 |
| miR-34c | 1417284_at | Mapkap1 | 0.484 | 0.022 |
| miR-34c | 1452960_at | Scyl3 | 0.484 | 0.023 |
| miR-34c | 1435914_at | Ncor1 | 0.484 | 0.023 |
| miR-34c | 1420824_at | Sema4d | 0.483 | 0.023 |
| miR-34c | 1448631_a_at | Hipk2 | -0.483 | 0.023 |
| miR-34c | 1419495_at | Immp2l | 0.483 | 0.023 |
| miR-34c | 1426315_a_at | 6330416G13Rik | 0.482 | 0.023 |
| miR-34c | 1418579_at | Cetn2 | 0.482 | 0.023 |
| miR-34c | 1435421_at | Fsd1 | -0.482 | 0.023 |
| miR-34c | 1455727_at | Zrsr2 | 0.481 | 0.023 |
| miR-34c | 1421450_a_at | Map3k4 | -0.481 | 0.023 |
| miR-34c | 1460582_x_at | NA | -0.481 | 0.023 |
| miR-34c | 1452271_at | Xpr1 | 0.481 | 0.024 |
| miR-34c | 1456357_at | A930041I02Rik | 0.481 | 0.024 |
| miR-34c | 1445892_at | NA | -0.481 | 0.024 |
| miR-34c | 1419170_at | Tmem157 | 0.480 | 0.024 |
| miR-34c | 1430295_at | Gna13 | 0.480 | 0.024 |
| miR-34c | 1440343_at | Rps6ka5 | 0.480 | 0.024 |
| miR-34c | 1456027_at | Rbm41 | 0.479 | 0.024 |
| miR-34c | 1416882_at | Rgs10 | 0.479 | 0.024 |
| miR-34c | 1443749_x_at | Slc1a3 | 0.479 | 0.024 |
| miR-34c | 1443160_at | Sbf2 | 0.479 | 0.024 |
| miR-34c | 1456897_at | NA | 0.478 | 0.024 |
| miR-34c | 1420932_at | Mapk8 | 0.478 | 0.024 |
| miR-34c | 1429192_at | Ski | 0.478 | 0.024 |
| miR-34c | 1418622_at | Rab2a | 0.477 | 0.025 |
| miR-34c | 1426435_at | Tmem135 | 0.477 | 0.025 |
| miR-34c | 1460241_a_at | St3gal5 | -0.477 | 0.025 |
| miR-34c | 1426219_at | Scp2 | 0.477 | 0.025 |
| miR-34c | 1426968_a_at | Rdh10 | 0.476 | 0.025 |
| miR-34c | 1427232_at | Tshz1 | -0.475 | 0.025 |
| miR-34c | 1427349_x_at | 2810021G02Rik | 0.475 | 0.025 |
| miR-34c | 1435556_at | Zfp597 | 0.475 | 0.026 |
| miR-34c | 1415791_at | Rnf34 | 0.474 | 0.026 |
| miR-34c | 1452008_at | 1810054D07Rik | 0.474 | 0.026 |
| miR-34c | 1434680_at | Plekhg3 | 0.474 | 0.026 |
| miR-34c | 1442003_at | Diap2 | 0.474 | 0.026 |
| miR-34c | 1456068_at | Nfasc | -0.474 | 0.026 |
| miR-34c | 1440427_at | Uvrag | 0.473 | 0.026 |
| miR-34c | 1427461_at | NA | -0.473 | 0.026 |
| miR-34c | 1452269_at | Spnb3 | -0.473 | 0.026 |
| miR-34c | 1429065_at | 1200009F10Rik | 0.473 | 0.026 |
| miR-34c | 1453146_at | Gapvd1 | -0.473 | 0.026 |
| miR-34c | 1439478_at | Acot2 | 0.473 | 0.026 |
| miR-34c | 1427460_at | LOC100046932 /// Taf4a | -0.472 | 0.026 |
| miR-34c | 1423383_a_at | Osbpl9 | 0.472 | 0.026 |
| miR-34c | 1460330_at | Anxa3 | 0.472 | 0.027 |
| miR-34c | 1436132_at | 9830001H06Rik | -0.472 | 0.027 |
| miR-34c | 1426244_at | Mapre2 | 0.472 | 0.027 |
| miR-34c | 1451125_at | Paip2b | 0.471 | 0.027 |
| miR-34c | 1418124_at | Tmem85 | 0.471 | 0.027 |
| miR-34c | 1424374_at | Gimap4 | 0.471 | 0.027 |
| miR-34c | 1447883_x_at | Map1lc3a | -0.471 | 0.027 |
| miR-34c | 1438024_at | NA | 0.471 | 0.027 |
| miR-34c | 1449198_a_at | St3gal5 | -0.471 | 0.027 |
| miR-34c | 1456463_at | A230106M20Rik | -0.471 | 0.027 |
| miR-34c | 1430417_s_at | 0610025P10Rik | -0.471 | 0.027 |
| miR-34c | 1458656_at | LOC100040174 /// LOC100047186 | -0.470 | 0.027 |
| miR-34c | 1418560_at | Pdha1 | 0.470 | 0.027 |
| miR-34c | 1456772_at | Ncf1 | 0.470 | 0.027 |
| miR-34c | 1433822_x_at | Anapc5 | -0.470 | 0.027 |
| miR-34c | 1452188_at | Maml1 | 0.470 | 0.027 |
| miR-34c | 1456026_at | Nhlrc3 | 0.469 | 0.028 |
| miR-34c | 1436535_at | Trove2 | 0.469 | 0.028 |
| miR-34c | 1436014_a_at | Rusc1 | 0.469 | 0.028 |
| miR-34c | 1427906_at | 1110037F02Rik | 0.469 | 0.028 |
| miR-34c | 1453848_s_at | Zbed3 | 0.468 | 0.028 |
| miR-34c | 1416281_at | Wdr45l | 0.468 | 0.028 |
| miR-34c | 1439490_at | Nr2c1 | 0.468 | 0.028 |
| miR-34c | 1425114_at | Rbbp6 | 0.467 | 0.028 |
| miR-34c | 1445299_at | NA | 0.467 | 0.028 |
| miR-34c | 1424391_at | Nrd1 | -0.467 | 0.028 |
| miR-34c | 1416912_at | 6330407G11Rik | 0.467 | 0.029 |
| miR-34c | 1429116_at | Slc17a5 | 0.466 | 0.029 |
| miR-34c | 1436077_a_at | Fcho1 | -0.466 | 0.029 |
| miR-34c | 1433673_at | E130309D14Rik | -0.466 | 0.029 |
| miR-34c | 1435554_at | Tmcc3 | 0.466 | 0.029 |
| miR-34c | 1450937_at | Lin7c | 0.466 | 0.029 |
| miR-34c | 1448255_a_at | Surf4 | 0.465 | 0.029 |
| miR-34c | 1460445_at | Sfrs2ip | 0.465 | 0.029 |
| miR-34c | 1449820_at | Cort | -0.465 | 0.029 |
| miR-34c | 1451396_at | Pomt2 | -0.465 | 0.029 |
| miR-34c | 1434567_at | 4732496O08Rik | 0.465 | 0.029 |
| miR-34c | 1453369_a_at | Fundc1 | 0.465 | 0.029 |
| miR-34c | 1438454_at | B430203M17Rik | 0.465 | 0.029 |
| miR-34c | 1417446_at | Slc12a4 | -0.465 | 0.029 |
| miR-34c | 1440179_x_at | Rnf217 | 0.464 | 0.029 |
| miR-34c | 1459871_x_at | Mar2 | -0.464 | 0.030 |
| miR-34c | 1427983_at | Zfp280c | 0.463 | 0.030 |
| miR-34c | 1417008_at | Crat | -0.463 | 0.030 |
| miR-34c | 1455349_at | LOC100048397 | 0.463 | 0.030 |
| miR-34c | 1429648_at | Slc35a3 | 0.463 | 0.030 |
| miR-34c | 1416472_at | Syap1 | 0.463 | 0.030 |
| miR-34c | 1421242_at | Rnf144a | -0.463 | 0.030 |
| miR-34c | 1448808_a_at | Nme2 | 0.462 | 0.030 |
| miR-34c | 1436078_at | Fcho1 | -0.462 | 0.030 |
| miR-34c | 1455388_at | Pcmtd1 | 0.462 | 0.030 |
| miR-34c | 1455263_at | 9030625A04Rik | 0.462 | 0.030 |
| miR-34c | 1443226_at | 5730470L24Rik | 0.462 | 0.031 |
| miR-34c | 1425192_at | Klhl25 | -0.462 | 0.031 |
| miR-34c | 1451074_at | Rnf13 | 0.462 | 0.031 |
| miR-34c | 1420473_at | Mtpn | 0.461 | 0.031 |
| miR-34c | 1441034_at | Itsn1 | -0.461 | 0.031 |
| miR-34c | 1436352_at | Cep78 | -0.461 | 0.031 |
| miR-34c | 1452009_at | 1810054D07Rik | 0.461 | 0.031 |
| miR-34c | 1419455_at | Il10rb | 0.461 | 0.031 |
| miR-34c | 1457268_at | Dot1l | -0.460 | 0.031 |
| miR-34c | 1436334_at | Synj1 | -0.460 | 0.031 |
| miR-34c | 1458276_x_at | Cit | -0.460 | 0.031 |
| miR-34c | 1415826_at | Atp6v1h | 0.460 | 0.031 |
| miR-34c | 1419829_a_at | NA | 0.459 | 0.031 |
| miR-34c | 1425980_at | Wdr54 | -0.459 | 0.032 |
| miR-34c | 1451990_at | Mapre2 | 0.459 | 0.032 |
| miR-34c | 1455836_at | Papola | -0.459 | 0.032 |
| miR-34c | 1450904_at | Tmem167 | 0.459 | 0.032 |
| miR-34c | 1422471_at | Pex13 | 0.459 | 0.032 |
| miR-34c | 1455227_at | Aadacl1 | 0.459 | 0.032 |
| miR-34c | 1426377_at | Zfp281 | 0.458 | 0.032 |
| miR-34c | 1429214_at | Adamtsl2 | -0.458 | 0.032 |
| miR-34c | 1426701_at | 4632419K20Rik | -0.458 | 0.032 |
| miR-34c | 1417077_at | Bcap29 | 0.457 | 0.032 |
| miR-34c | 1452871_at | Neil1 | 0.457 | 0.032 |
| miR-34c | 1424344_s_at | Eif1a | 0.457 | 0.032 |
| miR-34c | 1448570_at | Gmfb | 0.457 | 0.032 |
| miR-34c | 1451040_at | Dtd1 /// LOC100048650 | 0.457 | 0.032 |
| miR-34c | 1458497_at | NA | 0.457 | 0.033 |
| miR-34c | 1419613_at | Col7a1 | -0.457 | 0.033 |
| miR-34c | 1417164_at | Dusp10 | 0.457 | 0.033 |
| miR-34c | 1417071_s_at | Cyp4v3 | 0.457 | 0.033 |
| miR-34c | 1448110_at | Sema4a | 0.457 | 0.033 |
| miR-34c | 1428131_a_at | Cdc42se1 | 0.456 | 0.033 |
| miR-34c | 1417982_at | Insig2 | 0.456 | 0.033 |
| miR-34c | 1436979_x_at | Rbm14 | -0.456 | 0.033 |
| miR-34c | 1427166_a_at | Spg7 | -0.456 | 0.033 |
| miR-34c | 1445210_at | NA | 0.456 | 0.033 |
| miR-34c | 1444493_at | NA | -0.456 | 0.033 |
| miR-34c | 1427425_at | 9130208E07Rik | -0.455 | 0.033 |
| miR-34c | 1419512_at | Prpf40b | -0.455 | 0.033 |
| miR-34c | 1439024_at | Bag4 | 0.455 | 0.034 |
| miR-34c | 1458985_at | Fry | 0.455 | 0.034 |
| miR-34c | 1459353_at | Kctd3 | 0.454 | 0.034 |
| miR-34c | 1420892_at | Wnt7b | -0.454 | 0.034 |
| miR-34c | 1442601_at | Gmeb1 | 0.454 | 0.034 |
| miR-34c | 1416555_at | Ei24 | 0.454 | 0.034 |
| miR-34c | 1420877_at | Sept6 | 0.454 | 0.034 |
| miR-34c | 1459133_at | Edem3 | 0.453 | 0.034 |
| miR-34c | 1450464_at | E4f1 | -0.453 | 0.034 |
| miR-34c | 1454272_at | 9630015K15Rik | -0.453 | 0.034 |
| miR-34c | 1438198_at | Bri3bp | 0.453 | 0.034 |
| miR-34c | 1437680_x_at | Glrx2 | 0.453 | 0.034 |
| miR-34c | 1434062_at | Rabgap1l | 0.453 | 0.034 |
| miR-34c | 1455033_at | B430201A12Rik | 0.453 | 0.034 |
| miR-34c | 1455389_s_at | 2310051F07Rik | -0.453 | 0.034 |
| miR-34c | 1436244_a_at | Tle2 | -0.453 | 0.034 |
| miR-34c | 1430754_at | 4930511H11Rik | -0.452 | 0.034 |
| miR-34c | 1429418_at | Cdc14b | 0.452 | 0.035 |
| miR-34c | 1457247_at | NA | -0.452 | 0.035 |
| miR-34c | 1429253_at | Zmym4 | 0.452 | 0.035 |
| miR-34c | 1428293_at | 2310022M17Rik | 0.452 | 0.035 |
| miR-34c | 1457276_at | Snf1lk2 | -0.452 | 0.035 |
| miR-34c | 1428499_at | 2810454L23Rik | 0.452 | 0.035 |
| miR-34c | 1423488_at | Mmd | 0.452 | 0.035 |
| miR-34c | 1435444_at | Atf6 | 0.451 | 0.035 |
| miR-34c | 1436737_a_at | Sorbs1 | -0.451 | 0.035 |
| miR-34c | 1420836_at | Slc25a30 | 0.451 | 0.035 |
| miR-34c | 1451520_at | Spg20 | 0.451 | 0.035 |
| miR-34c | 1434080_at | Aebp2 | 0.451 | 0.035 |
| miR-34c | 1460222_at | Sh3bp1 | 0.451 | 0.035 |
| miR-34c | 1422693_a_at | Sub1 | 0.451 | 0.035 |
| miR-34c | 1448778_at | Sfrs4 | -0.451 | 0.035 |
| miR-34c | 1425030_at | Zfp622 | 0.450 | 0.036 |
| miR-34c | 1417361_at | Asb3 | 0.450 | 0.036 |
| miR-34c | 1438115_a_at | Slc9a3r1 | 0.450 | 0.036 |
| miR-34c | 1417307_at | Dmd | 0.450 | 0.036 |
| miR-34c | 1434256_s_at | Cds2 | -0.450 | 0.036 |
| miR-34c | 1440876_at | NA | 0.450 | 0.036 |
| miR-34c | 1423928_at | Ubac2 | 0.449 | 0.036 |
| miR-34c | 1422442_at | Smu1 | 0.449 | 0.036 |
| miR-34c | 1430294_at | Ssbp1 | 0.449 | 0.036 |
| miR-34c | 1435727_s_at | Lima1 | 0.449 | 0.036 |
| miR-34c | 1423560_at | Nell2 | -0.449 | 0.036 |
| miR-34c | 1457248_x_at | Hsd17b7 | 0.448 | 0.037 |
| miR-34c | 1424433_at | Msrb2 | 0.448 | 0.037 |
| miR-34c | 1427306_at | Ryr1 | 0.448 | 0.037 |
| miR-34c | 1428422_at | Ubxd8 | 0.448 | 0.037 |
| miR-34c | 1431372_at | Srpk2 | 0.448 | 0.037 |
| miR-34c | 1422634_a_at | Vsig2 | -0.448 | 0.037 |
| miR-34c | 1444236_at | AW552393 | 0.447 | 0.037 |
| miR-34c | 1435220_s_at | Cdc42se2 | 0.447 | 0.037 |
| miR-34c | 1421160_a_at | Rfng | -0.447 | 0.037 |
| miR-34c | 1454671_at | Insig1 | 0.447 | 0.037 |
| miR-34c | 1418089_at | Stx8 | 0.447 | 0.037 |
| miR-34c | 1455335_at | Xrcc2 | -0.447 | 0.037 |
| miR-34c | 1452148_at | Lrpap1 | 0.447 | 0.037 |
| miR-34c | 1443830_x_at | Rnf103 | 0.447 | 0.037 |
| miR-34c | 1422032_a_at | Zfand6 | -0.446 | 0.037 |
| miR-34c | 1453221_at | Gopc | 0.446 | 0.037 |
| miR-34c | 1448681_at | Il15ra | 0.446 | 0.037 |
| miR-34c | 1423829_at | 0910001A06Rik | 0.446 | 0.037 |
| miR-34c | 1448957_at | Rbpj | 0.446 | 0.038 |
| miR-34c | 1439847_s_at | Klf12 | 0.446 | 0.038 |
| miR-34c | 1437380_x_at | Pgd | 0.446 | 0.038 |
| miR-34c | 1436537_at | Zfp629 | 0.446 | 0.038 |
| miR-34c | 1434515_at | Ncoa1 | 0.446 | 0.038 |
| miR-34c | 1443678_at | NA | -0.445 | 0.038 |
| miR-34c | 1423131_at | 5730427N09Rik /// EG433230 /// EG547215 /// LOC100042660 /// LOC239338 /// LOC623804 /// LOC627895 /// LOC629436 /// LOC630127 /// LOC636306 /// LOC665356 /// LOC666180 /// LOC675063 /// LOC677785 | 0.445 | 0.038 |
| miR-34c | 1440579_at | NA | 0.445 | 0.038 |
| miR-34c | 1430038_at | NA | 0.445 | 0.038 |
| miR-34c | 1455352_at | 2610101N10Rik | 0.445 | 0.038 |
| miR-34c | 1431939_a_at | Mina | 0.445 | 0.038 |
| miR-34c | 1439407_x_at | Tagln2 | 0.445 | 0.038 |
| miR-34c | 1419696_at | Cd4 | 0.444 | 0.038 |
| miR-34c | 1435302_at | Taf4b | 0.443 | 0.039 |
| miR-34c | 1416094_at | Adam9 | 0.443 | 0.039 |
| miR-34c | 1427114_at | Ttc19 | 0.443 | 0.039 |
| miR-34c | 1416440_at | Cd164 | 0.443 | 0.039 |
| miR-34c | 1415697_at | G3bp2 | 0.443 | 0.039 |
| miR-34c | 1424215_at | Fundc1 | 0.443 | 0.039 |
| miR-34c | 1453216_at | Gpt | -0.442 | 0.039 |
| miR-34c | 1426998_at | Zfand3 | 0.442 | 0.039 |
| miR-34c | 1439328_at | Nfat5 | 0.442 | 0.039 |
| miR-34c | 1416771_at | Trappc3 | 0.442 | 0.040 |
| miR-34c | 1434035_at | Dnajb6 /// LOC100048324 | -0.442 | 0.040 |
| miR-34c | 1422505_at | Chrac1 | 0.442 | 0.040 |
| miR-34c | 1417676_a_at | Ptpro | 0.442 | 0.040 |
| miR-34c | 1419493_a_at | Tpd52 | 0.441 | 0.040 |
| miR-34c | 1420928_at | St6gal1 | 0.441 | 0.040 |
| miR-34c | 1452782_a_at | Txn2 | -0.441 | 0.040 |
| miR-34c | 1437994_x_at | Mier2 | -0.441 | 0.040 |
| miR-34c | 1426981_at | Pcsk6 | -0.440 | 0.040 |
| miR-34c | 1456177_x_at | Zfp706 | 0.440 | 0.040 |
| miR-34c | 1435099_at | Utp14a | 0.440 | 0.041 |
| miR-34c | 1434418_at | Lass6 | 0.440 | 0.041 |
| miR-34c | 1457610_at | NA | -0.440 | 0.041 |
| miR-34c | 1448442_a_at | LOC100038973 /// Psma3 | 0.439 | 0.041 |
| miR-34c | 1438012_at | Ppm1l | -0.439 | 0.041 |
| miR-34c | 1433684_at | Chmp6 | 0.439 | 0.041 |
| miR-34c | 1435755_at | 1110001A16Rik | 0.439 | 0.041 |
| miR-34c | 1451075_s_at | Ctdsp2 | 0.439 | 0.041 |
| miR-34c | 1434404_at | C030011O14Rik | 0.439 | 0.041 |
| miR-34c | 1418086_at | Ppp1r14a | 0.439 | 0.041 |
| miR-34c | 1454806_at | D12Ertd553e | 0.439 | 0.041 |
| miR-34c | 1416974_at | Stam2 | 0.439 | 0.041 |
| miR-34c | 1432543_a_at | Klf13 | -0.439 | 0.041 |
| miR-34c | 1428221_at | Klhdc8b | -0.438 | 0.041 |
| miR-34c | 1418356_at | Mpst | -0.438 | 0.041 |
| miR-34c | 1428600_at | Nin | 0.438 | 0.042 |
| miR-34c | 1417955_at | Ccdc71 | 0.438 | 0.042 |
| miR-34c | 1424026_s_at | BC013529 | 0.438 | 0.042 |
| miR-34c | 1417235_at | Ehd3 | -0.437 | 0.042 |
| miR-34c | 1451163_at | Tinf2 | -0.437 | 0.042 |
| miR-34c | 1432158_a_at | Trappc2 | 0.437 | 0.042 |
| miR-34c | 1455291_s_at | Znrf2 | 0.437 | 0.042 |
| miR-34c | 1449173_at | Mpp2 | -0.437 | 0.042 |
| miR-34c | 1427777_x_at | Fgfr4 | -0.437 | 0.042 |
| miR-34c | 1428218_a_at | 1600012H06Rik | 0.437 | 0.042 |
| miR-34c | 1450018_s_at | Slc25a30 | 0.436 | 0.042 |
| miR-34c | 1452347_at | Mef2a | 0.436 | 0.043 |
| miR-34c | 1436401_at | 9330128J19Rik | 0.436 | 0.043 |
| miR-34c | 1423538_at | Ntan1 | 0.436 | 0.043 |
| miR-34c | 1451570_a_at | 6720467C03Rik | 0.436 | 0.043 |
| miR-34c | 1450881_s_at | Gpr137b | 0.436 | 0.043 |
| miR-34c | 1417059_at | Krtcap2 | 0.435 | 0.043 |
| miR-34c | 1416216_at | Reps1 | 0.435 | 0.043 |
| miR-34c | 1427120_at | Zfp26 | 0.435 | 0.043 |
| miR-34c | 1420888_at | Bcl2l1 | -0.434 | 0.043 |
| miR-34c | 1443733_x_at | Pold3 | -0.434 | 0.043 |
| miR-34c | 1421578_at | Ccl4 | 0.434 | 0.043 |
| miR-34c | 1423619_at | Rasd1 | -0.434 | 0.044 |
| miR-34c | 1443722_at | NA | -0.434 | 0.044 |
| miR-34c | 1458684_at | NA | 0.434 | 0.044 |
| miR-34c | 1428661_at | Nfkbil2 | -0.434 | 0.044 |
| miR-34c | 1426758_s_at | Meg3 | -0.434 | 0.044 |
| miR-34c | 1430961_at | Zfp292 | 0.434 | 0.044 |
| miR-34c | 1434478_at | Heca | 0.434 | 0.044 |
| miR-34c | 1448431_at | Asb6 | -0.434 | 0.044 |
| miR-34c | 1416356_at | Gmpr2 | 0.434 | 0.044 |
| miR-34c | 1452673_at | Ranbp3 | -0.434 | 0.044 |
| miR-34c | 1428676_at | Tmprss6 | -0.434 | 0.044 |
| miR-34c | 1416002_x_at | Cotl1 | 0.433 | 0.044 |
| miR-34c | 1416359_at | Snx18 | 0.433 | 0.044 |
| miR-34c | 1447090_s_at | Arl1 | 0.433 | 0.044 |
| miR-34c | 1452797_at | Fastkd3 | 0.433 | 0.044 |
| miR-34c | 1450442_at | Add2 | -0.433 | 0.044 |
| miR-34c | 1457430_at | NA | -0.433 | 0.044 |
| miR-34c | 1427361_at | Hoxc6 | -0.432 | 0.044 |
| miR-34c | 1454736_at | Ankrd57 | 0.432 | 0.044 |
| miR-34c | 1448896_at | Pigf | 0.432 | 0.045 |
| miR-34c | 1460436_at | Ndst1 | 0.432 | 0.045 |
| miR-34c | 1420999_at | Cnot4 | 0.432 | 0.045 |
| miR-34c | 1438580_at | Zcchc7 | 0.432 | 0.045 |
| miR-34c | 1445801_at | NA | -0.432 | 0.045 |
| miR-34c | 1434187_at | Alg11 | 0.432 | 0.045 |
| miR-34c | 1419165_at | Zfp260 | 0.431 | 0.045 |
| miR-34c | 1417779_at | 2310079N02Rik | 0.431 | 0.045 |
| miR-34c | 1428657_at | Rreb1 | 0.431 | 0.045 |
| miR-34c | 1453321_at | Fndc1 | -0.431 | 0.045 |
| miR-34c | AFFX-PyruCarbMur/L09192_MA_at | Pcx | -0.431 | 0.045 |
| miR-34c | 1449517_at | Qpctl | 0.431 | 0.045 |
| miR-34c | 1426963_at | Pacs2 | -0.430 | 0.046 |
| miR-34c | 1419745_at | 4933428G20Rik | -0.430 | 0.046 |
| miR-34c | 1426903_at | Fndc3a | 0.430 | 0.046 |
| miR-34c | 1417426_at | Srgn | 0.430 | 0.046 |
| miR-34c | 1421323_a_at | G3bp2 | 0.430 | 0.046 |
| miR-34c | 1439033_at | Zcchc7 | 0.430 | 0.046 |
| miR-34c | 1442135_at | Ado | 0.430 | 0.046 |
| miR-34c | 1451794_at | Tmcc3 | 0.429 | 0.046 |
| miR-34c | 1450031_at | Aff4 | 0.429 | 0.046 |
| miR-34c | 1426461_at | Ugp2 | 0.429 | 0.046 |
| miR-34c | 1450647_at | Hps3 | 0.429 | 0.046 |
| miR-34c | 1431811_a_at | Fbxo34 | -0.429 | 0.046 |
| miR-34c | 1417977_at | Eif4e3 | 0.429 | 0.046 |
| miR-34c | 1424657_at | Taok1 | 0.429 | 0.046 |
| miR-34c | 1426411_a_at | C230082I21Rik /// Strbp | 0.429 | 0.046 |
| miR-34c | 1459859_x_at | Chrac1 | 0.429 | 0.046 |
| miR-34c | 1418436_at | Stx7 | 0.429 | 0.047 |
| miR-34c | 1434005_at | Rbms1 | 0.429 | 0.047 |
| miR-34c | 1457706_at | BC030336 | 0.428 | 0.047 |
| miR-34c | 1419091_a_at | Anxa2 | 0.428 | 0.047 |
| miR-34c | 1442050_at | Zfp608 | 0.428 | 0.047 |
| miR-34c | 1421029_a_at | Hbs1l | -0.428 | 0.047 |
| miR-34c | 1457447_at | NA | -0.428 | 0.047 |
| miR-34c | 1416880_at | Mcl1 | 0.428 | 0.047 |
| miR-34c | 1428559_at | Kdsr | 0.428 | 0.047 |
| miR-34c | 1452402_at | NA | 0.428 | 0.047 |
| miR-34c | 1424424_at | LOC635418 /// Slc39a1 | 0.428 | 0.047 |
| miR-34c | 1434708_at | Vhlh | 0.427 | 0.047 |
| miR-34c | 1453681_at | Atpif1 | -0.427 | 0.047 |
| miR-34c | 1423449_a_at | Actn4 | -0.427 | 0.047 |
| miR-34c | 1420307_a_at | Pitpnb | 0.427 | 0.047 |
| miR-34c | 1435793_at | Aph1b | 0.427 | 0.047 |
| miR-34c | 1438117_x_at | Tmem41b | -0.427 | 0.047 |
| miR-34c | 1431818_at | 1700012B15Rik | 0.427 | 0.047 |
| miR-34c | 1437119_at | Ern1 | 0.427 | 0.047 |
| miR-34c | 1423552_at | Leprotl1 | 0.427 | 0.048 |
| miR-34c | 1451124_at | Sod1 | -0.427 | 0.048 |
| miR-34c | 1416772_at | Cpt2 | -0.427 | 0.048 |
| miR-34c | 1416618_at | Ppox | -0.427 | 0.048 |
| miR-34c | 1424707_at | Tmed10 | 0.427 | 0.048 |
| miR-34c | 1423849_a_at | Clk3 | -0.427 | 0.048 |
| miR-34c | 1417655_a_at | Ars2 | -0.426 | 0.048 |
| miR-34c | 1434832_at | Foxo3a | -0.426 | 0.048 |
| miR-34c | 1447412_at | Gm996 | -0.426 | 0.048 |
| miR-34c | 1416264_at | Abcb9 | -0.426 | 0.048 |
| miR-34c | 1434936_at | Hirip3 | -0.426 | 0.048 |
| miR-34c | 1423133_at | Cwc15 | 0.426 | 0.048 |
| miR-34c | 1429201_at | Cyld | 0.426 | 0.048 |
| miR-34c | 1422719_s_at | Nup50 | -0.425 | 0.048 |
| miR-34c | 1438116_x_at | Slc9a3r1 | 0.425 | 0.049 |
| miR-34c | 1423252_at | Hdgfrp3 | 0.425 | 0.049 |
| miR-34c | 1427299_at | Rps6ka3 | 0.425 | 0.049 |
| miR-34c | 1417539_at | LOC100046775 /// Slc35a1 | 0.425 | 0.049 |
| miR-34c | 1439405_x_at | Cntd1 | -0.425 | 0.049 |
| miR-34c | 1417648_s_at | Snx5 | 0.425 | 0.049 |
| miR-34c | 1434546_at | Smg5 | -0.424 | 0.049 |
| miR-34c | 1448356_at | Ube2d2 | 0.424 | 0.049 |
| miR-34c | 1452387_a_at | Amotl2 | 0.424 | 0.049 |
| miR-34c | 1421397_a_at | Lrdd | -0.424 | 0.049 |
| miR-34c | 1434413_at | Igf1 | 0.424 | 0.049 |
| miR-34c | 1448109_a_at | Rpl26 | 0.424 | 0.049 |
| miR-34c | 1429399_at | Rnf125 | 0.424 | 0.049 |
| miR-34c | 1435869_s_at | Ap2a2 | -0.424 | 0.049 |
| miR-34c | 1428645_at | Gnai3 | 0.424 | 0.049 |
| miR-34c | 1447696_x_at | Adcy5 /// LOC100047385 | -0.424 | 0.049 |
| miR-34c | 1423694_at | Kctd10 | 0.424 | 0.049 |
| miR-34c | 1423097_s_at | Capn7 | 0.424 | 0.049 |
| miR-34c | 1428471_at | Sorbs1 | -0.423 | 0.050 |
| miR-34c | 1459488_at | NA | 0.423 | 0.050 |
| miR-34c | 1423707_at | Tmem50b | 0.423 | 0.050 |
| miR-34c | 1433722_at | Akap13 | 0.423 | 0.050 |
| miR-34c | 1449959_x_at | Lce1h | -0.423 | 0.050 |
| miR-34c | 1451000_at | Tmem126a | 0.423 | 0.050 |
| miR-34c | 1434743_x_at | Rusc1 | 0.423 | 0.050 |
| miR-212 | 1423647_a_at | Zdhhc3 | -0.756 | 0.000 |
| miR-212 | 1439253_x_at | LOC100046650 /// Prelid1 | 0.659 | 0.001 |
| miR-212 | 1419470_at | Gnb4 | 0.651 | 0.001 |
| miR-212 | 1456795_at | D330027G24Rik | 0.647 | 0.001 |
| miR-212 | 1457707_at | Mctp2 | 0.644 | 0.001 |
| miR-212 | 1428327_at | Trak1 | 0.644 | 0.001 |
| miR-212 | 1439526_at | NA | 0.629 | 0.002 |
| miR-212 | 1419469_at | Gnb4 | 0.626 | 0.002 |
| miR-212 | 1447234_s_at | Snx6 | 0.625 | 0.002 |
| miR-212 | 1446835_at | NA | 0.621 | 0.002 |
| miR-212 | 1448202_x_at | LOC100046650 /// LOC675389 /// Prelid1 | 0.619 | 0.002 |
| miR-212 | 1416860_s_at | Ing1 | 0.617 | 0.002 |
| miR-212 | 1440613_at | NA | -0.613 | 0.002 |
| miR-212 | 1421972_s_at | Hcfc1 | -0.612 | 0.002 |
| miR-212 | 1438580_at | Zcchc7 | 0.611 | 0.002 |
| miR-212 | 1426895_at | Zfp191 | 0.610 | 0.003 |
| miR-212 | 1420887_a_at | Bcl2l1 | -0.610 | 0.003 |
| miR-212 | 1456323_at | Pofut1 | -0.609 | 0.003 |
| miR-212 | 1460438_at | LOC100044319 /// Lysmd1 | -0.606 | 0.003 |
| miR-212 | 1454664_a_at | Eif5 /// LOC100047658 | -0.606 | 0.003 |
| miR-212 | 1417317_s_at | Inoc1 /// LOC100040169 /// LOC100042363 /// LOC100042824 /// LOC100043341 /// LOC100043423 /// LOC100044516 /// LOC100045041 /// LOC100045334 /// LOC100045640 /// LOC100046034 /// LOC100048491 /// LOC100048620 /// Rpl35a | 0.604 | 0.003 |
| miR-212 | 1435822_at | D830012I24Rik | 0.603 | 0.003 |
| miR-212 | 1430245_at | NA | 0.602 | 0.003 |
| miR-212 | 1424433_at | Msrb2 | 0.602 | 0.003 |
| miR-212 | 1448661_at | Plcb3 | 0.597 | 0.003 |
| miR-212 | 1437317_at | Ube1l | -0.593 | 0.004 |
| miR-212 | 1450937_at | Lin7c | 0.593 | 0.004 |
| miR-212 | 1456716_s_at | 3110002H16Rik | -0.592 | 0.004 |
| miR-212 | 1417576_a_at | Otub2 | -0.590 | 0.004 |
| miR-212 | 1452737_at | 2810008M24Rik /// LOC100046418 | 0.589 | 0.004 |
| miR-212 | 1415956_a_at | Pctk1 | -0.588 | 0.004 |
| miR-212 | 1439493_at | Zfp827 | 0.587 | 0.004 |
| miR-212 | 1422485_at | LOC100048076 /// Smad4 | 0.586 | 0.004 |
| miR-212 | 1449816_at | Sult5a1 | -0.579 | 0.005 |
| miR-212 | 1434036_at | Mtss1 | 0.577 | 0.005 |
| miR-212 | 1428791_at | Ube2h | -0.577 | 0.005 |
| miR-212 | 1424656_s_at | Usp19 | -0.576 | 0.005 |
| miR-212 | 1420477_at | Nap1l1 | 0.576 | 0.005 |
| miR-212 | 1450540_x_at | Krtap5-1 | -0.575 | 0.005 |
| miR-212 | 1420473_at | Mtpn | 0.575 | 0.005 |
| miR-212 | 1425492_at | Bmpr1a | 0.574 | 0.005 |
| miR-212 | 1434278_at | Mtm1 | -0.574 | 0.005 |
| miR-212 | 1426526_s_at | Ovgp1 | 0.571 | 0.005 |
| miR-212 | 1458802_at | Hivep3 | 0.570 | 0.006 |
| miR-212 | 1451474_a_at | Parp8 | 0.570 | 0.006 |
| miR-212 | 1456440_s_at | St8sia6 | 0.570 | 0.006 |
| miR-212 | 1422486_a_at | LOC100048076 /// Smad4 | 0.569 | 0.006 |
| miR-212 | 1437341_x_at | Cnp | 0.568 | 0.006 |
| miR-212 | 1437874_s_at | Hexb | 0.566 | 0.006 |
| miR-212 | 1450066_at | Ubr1 | 0.566 | 0.006 |
| miR-212 | 1425192_at | Klhl25 | -0.565 | 0.006 |
| miR-212 | 1427447_a_at | Triobp | -0.564 | 0.006 |
| miR-212 | 1436050_x_at | Hes6 | 0.562 | 0.007 |
| miR-212 | 1436928_s_at | Adcy3 | -0.561 | 0.007 |
| miR-212 | 1428657_at | Rreb1 | 0.561 | 0.007 |
| miR-212 | 1454757_s_at | D12Ertd647e | -0.559 | 0.007 |
| miR-212 | 1434572_at | Hdac9 | 0.558 | 0.007 |
| miR-212 | 1418632_at | Ube2h | -0.558 | 0.007 |
| miR-212 | 1418029_at | Faim | 0.558 | 0.007 |
| miR-212 | 1417010_at | Zfp238 | 0.557 | 0.007 |
| miR-212 | 1447923_at | 1810026B05Rik | 0.556 | 0.007 |
| miR-212 | 1423383_a_at | Osbpl9 | 0.556 | 0.007 |
| miR-212 | 1455206_at | NA | 0.555 | 0.007 |
| miR-212 | 1456303_at | NA | 0.554 | 0.007 |
| miR-212 | 1425520_a_at | Dhdds | -0.553 | 0.008 |
| miR-212 | 1454900_s_at | Mycbp2 | 0.553 | 0.008 |
| miR-212 | 1448388_a_at | 1110002B05Rik | 0.553 | 0.008 |
| miR-212 | 1450439_at | Hcfc1 | -0.552 | 0.008 |
| miR-212 | 1415716_a_at | Rps27 | 0.552 | 0.008 |
| miR-212 | 1440721_at | 5930433N17Rik | 0.551 | 0.008 |
| miR-212 | 1457636_x_at | NA | 0.551 | 0.008 |
| miR-212 | 1429281_at | 2610008E11Rik | 0.551 | 0.008 |
| miR-212 | 1435260_at | Akt3 | 0.550 | 0.008 |
| miR-212 | 1433542_at | Inpp5f | 0.549 | 0.008 |
| miR-212 | 1452214_at | Skil | 0.549 | 0.008 |
| miR-212 | 1434631_at | D3Ertd751e | -0.548 | 0.008 |
| miR-212 | 1452616_s_at | Ssbp1 | 0.548 | 0.008 |
| miR-212 | 1425899_a_at | Itsn1 | -0.547 | 0.008 |
| miR-212 | 1451190_a_at | Sbk1 | 0.547 | 0.008 |
| miR-212 | 1439652_at | NA | 0.547 | 0.008 |
| miR-212 | 1425680_a_at | Btrc | -0.547 | 0.008 |
| miR-212 | 1460544_at | Mak10 | 0.545 | 0.009 |
| miR-212 | 1436016_x_at | Gdi2 | 0.545 | 0.009 |
| miR-212 | 1432211_a_at | Fbxo9 | -0.545 | 0.009 |
| miR-212 | 1416904_at | Mbnl1 | 0.544 | 0.009 |
| miR-212 | 1434010_at | Als2cr13 | 0.544 | 0.009 |
| miR-212 | 1435203_at | Man2a2 | 0.544 | 0.009 |
| miR-212 | 1448188_at | Ucp2 | -0.543 | 0.009 |
| miR-212 | 1454843_at | Prps2 | 0.543 | 0.009 |
| miR-212 | 1455183_at | Stk38l | 0.543 | 0.009 |
| miR-212 | 1434897_a_at | Slc25a4 | 0.542 | 0.009 |
| miR-212 | 1421890_at | St3gal2 | -0.542 | 0.009 |
| miR-212 | 1455138_x_at | Cfl1 | 0.541 | 0.009 |
| miR-212 | 1453111_a_at | Slc25a39 | -0.540 | 0.009 |
| miR-212 | 1436153_a_at | Zmynd11 | 0.540 | 0.010 |
| miR-212 | 1433754_at | Mbnl2 | 0.540 | 0.010 |
| miR-212 | 1458218_s_at | Pde7a | 0.539 | 0.010 |
| miR-212 | 1416989_at | Vps53 | 0.539 | 0.010 |
| miR-212 | 1444415_at | NA | -0.539 | 0.010 |
| miR-212 | 1432426_a_at | Ube2f | -0.539 | 0.010 |
| miR-212 | 1433773_at | Rrm2b | 0.539 | 0.010 |
| miR-212 | 1436753_at | Adck5 | -0.538 | 0.010 |
| miR-212 | 1424283_at | Jtb | 0.538 | 0.010 |
| miR-212 | 1438442_at | 5730470L24Rik | 0.538 | 0.010 |
| miR-212 | 1460290_at | Lpin2 | -0.537 | 0.010 |
| miR-212 | 1426530_a_at | Klhl5 | 0.537 | 0.010 |
| miR-212 | 1434956_at | Rnf170 | 0.536 | 0.010 |
| miR-212 | 1427461_at | NA | -0.536 | 0.010 |
| miR-212 | 1454923_at | Iws1 | 0.535 | 0.010 |
| miR-212 | 1436317_at | Pgap1 | 0.535 | 0.010 |
| miR-212 | 1439722_at | NA | 0.535 | 0.010 |
| miR-212 | 1455217_at | Lrig2 | 0.534 | 0.010 |
| miR-212 | 1424873_at | Rnf2 | 0.534 | 0.011 |
| miR-212 | 1421077_at | Sertad3 | -0.533 | 0.011 |
| miR-212 | 1434690_at | Lycat | 0.533 | 0.011 |
| miR-212 | 1437110_at | 2810474O19Rik | -0.533 | 0.011 |
| miR-212 | 1442050_at | Zfp608 | 0.533 | 0.011 |
| miR-212 | 1459741_x_at | Ucp2 | -0.532 | 0.011 |
| miR-212 | 1426009_a_at | Pip5k1a | -0.532 | 0.011 |
| miR-212 | 1434184_s_at | Map4k4 | 0.531 | 0.011 |
| miR-212 | 1452671_s_at | Lman1 | -0.531 | 0.011 |
| miR-212 | 1455594_at | Exoc3 | 0.530 | 0.011 |
| miR-212 | 1419994_s_at | D10Ertd641e | 0.530 | 0.011 |
| miR-212 | 1433746_at | Wdr3 | 0.529 | 0.011 |
| miR-212 | 1434937_at | Mycbp2 | 0.528 | 0.011 |
| miR-212 | 1460241_a_at | St3gal5 | -0.528 | 0.012 |
| miR-212 | 1448356_at | Ube2d2 | 0.528 | 0.012 |
| miR-212 | 1455485_x_at | Rpl13a | 0.528 | 0.012 |
| miR-212 | 1423829_at | 0910001A06Rik | 0.527 | 0.012 |
| miR-212 | 1448570_at | Gmfb | 0.527 | 0.012 |
| miR-212 | 1428131_a_at | Cdc42se1 | 0.526 | 0.012 |
| miR-212 | 1441172_at | NA | 0.526 | 0.012 |
| miR-212 | 1435875_at | Prkab2 | 0.526 | 0.012 |
| miR-212 | 1452600_at | Taf6l | -0.525 | 0.012 |
| miR-212 | 1452956_a_at | D12Ertd647e | -0.525 | 0.012 |
| miR-212 | 1455767_x_at | LOC100039601 /// LOC100041000 /// LOC100042650 /// LOC100043348 /// LOC100043495 /// LOC100044606 /// LOC100045613 /// LOC100045641 /// LOC100046290 /// LOC100046478 /// LOC100047314 /// LOC100048241 /// LOC675980 /// LOC676253 /// mCG_121688 /// Rpl21 | 0.525 | 0.012 |
| miR-212 | 1427117_at | Mtmr3 | -0.525 | 0.012 |
| miR-212 | 1427965_at | Ssbp1 | 0.525 | 0.012 |
| miR-212 | 1424642_at | Thoc1 | 0.524 | 0.012 |
| miR-212 | 1435930_at | Scaper | 0.524 | 0.012 |
| miR-212 | 1416983_s_at | Foxo1 | 0.524 | 0.012 |
| miR-212 | 1428946_at | Uba6 | 0.524 | 0.012 |
| miR-212 | 1418884_x_at | Tuba1a | 0.523 | 0.013 |
| miR-212 | 1434080_at | Aebp2 | 0.522 | 0.013 |
| miR-212 | 1442034_at | Fastkd1 | -0.522 | 0.013 |
| miR-212 | 1424337_at | Snx15 | -0.522 | 0.013 |
| miR-212 | 1427493_at | Sclt1 | -0.522 | 0.013 |
| miR-212 | 1458469_at | Cblb | 0.522 | 0.013 |
| miR-212 | 1420000_s_at | Igbp1 | 0.522 | 0.013 |
| miR-212 | 1422693_a_at | Sub1 | 0.521 | 0.013 |
| miR-212 | 1448707_at | Taf13 | 0.521 | 0.013 |
| miR-212 | 1439459_x_at | Acly | 0.520 | 0.013 |
| miR-212 | 1415920_at | Cstf2t | 0.519 | 0.013 |
| miR-212 | 1444092_at | 9430025M13Rik | 0.519 | 0.013 |
| miR-212 | 1456628_x_at | Rps24 | 0.518 | 0.013 |
| miR-212 | 1448999_at | Trappc5 | 0.518 | 0.013 |
| miR-212 | 1434316_at | Chsy1 /// LOC100047167 | 0.518 | 0.013 |
| miR-212 | 1426963_at | Pacs2 | -0.518 | 0.014 |
| miR-212 | 1429125_at | Zbtb9 | 0.518 | 0.014 |
| miR-212 | 1457898_at | NA | 0.517 | 0.014 |
| miR-212 | 1430381_at | Ftsj1 | -0.516 | 0.014 |
| miR-212 | 1428829_at | 6820401H01Rik | 0.516 | 0.014 |
| miR-212 | 1456022_at | Hipk2 | -0.515 | 0.014 |
| miR-212 | 1430043_at | Ttc19 | 0.514 | 0.014 |
| miR-212 | 1455286_at | Btbd1 | 0.514 | 0.014 |
| miR-212 | 1416691_at | Gtpbp2 | -0.513 | 0.015 |
| miR-212 | 1426527_at | Toe1 | 0.513 | 0.015 |
| miR-212 | 1436941_at | Gm1752 | 0.513 | 0.015 |
| miR-212 | 1439255_s_at | Gpr137b /// Gpr137b-ps /// LOC100044979 | 0.513 | 0.015 |
| miR-212 | 1420388_at | Prss12 | 0.512 | 0.015 |
| miR-212 | 1416388_at | Pip4k2c | -0.512 | 0.015 |
| miR-212 | 1450275_x_at | Ube2f | -0.512 | 0.015 |
| miR-212 | 1448689_at | Rras2 | 0.512 | 0.015 |
| miR-212 | 1429610_a_at | Zfp511 | -0.511 | 0.015 |
| miR-212 | 1440167_s_at | Lpp | 0.511 | 0.015 |
| miR-212 | 1423083_at | Rab33b | 0.511 | 0.015 |
| miR-212 | 1443747_at | D3Ertd300e | 0.510 | 0.015 |
| miR-212 | 1439278_at | Zbtb20 | 0.509 | 0.015 |
| miR-212 | 1450086_at | Gmeb1 | -0.509 | 0.016 |
| miR-212 | 1424540_at | Hipk1 | -0.509 | 0.016 |
| miR-212 | 1419033_at | 2610018G03Rik | 0.509 | 0.016 |
| miR-212 | 1419165_at | Zfp260 | 0.509 | 0.016 |
| miR-212 | 1437741_at | Rab21 | 0.508 | 0.016 |
| miR-212 | 1460707_at | Ptp4a2 | 0.508 | 0.016 |
| miR-212 | 1454899_at | Lpp | 0.508 | 0.016 |
| miR-212 | 1451114_at | Cmtm6 | 0.507 | 0.016 |
| miR-212 | 1417062_at | Armc10 | 0.507 | 0.016 |
| miR-212 | 1438041_at | Pde7a | 0.507 | 0.016 |
| miR-212 | 1434016_at | Znrf2 | 0.507 | 0.016 |
| miR-212 | 1423350_at | Socs5 | 0.507 | 0.016 |
| miR-212 | 1455314_at | Lpp | 0.507 | 0.016 |
| miR-212 | 1426753_at | Phf17 | 0.507 | 0.016 |
| miR-212 | 1460121_at | 9630010G10Rik | 0.506 | 0.016 |
| miR-212 | 1452665_at | Ttc27 | 0.506 | 0.016 |
| miR-212 | 1425148_a_at | LOC100047864 /// Snx6 | 0.505 | 0.017 |
| miR-212 | 1422018_at | Hivep2 | 0.505 | 0.017 |
| miR-212 | 1433771_at | 5730446C15Rik | 0.504 | 0.017 |
| miR-212 | 1430369_at | Epb4.1 | -0.504 | 0.017 |
| miR-212 | 1424026_s_at | BC013529 | 0.503 | 0.017 |
| miR-212 | 1439336_at | NA | 0.503 | 0.017 |
| miR-212 | 1455827_at | Mbnl2 | 0.502 | 0.017 |
| miR-212 | 1433748_at | Zdhhc18 | 0.502 | 0.017 |
| miR-212 | 1426879_at | 1190005F20Rik | 0.502 | 0.017 |
| miR-212 | 1416504_at | Ulk1 | -0.502 | 0.017 |
| miR-212 | 1416841_at | 1110059E24Rik | 0.501 | 0.017 |
| miR-212 | 1434953_at | Ccdc93 | 0.501 | 0.018 |
| miR-212 | 1429430_at | Pcmtd1 | 0.501 | 0.018 |
| miR-212 | 1448663_s_at | Mvd | 0.500 | 0.018 |
| miR-212 | 1419246_s_at | Rab14 | 0.500 | 0.018 |
| miR-212 | 1437728_at | Alkbh5 | -0.500 | 0.018 |
| miR-212 | 1448960_at | Cxxc5 | 0.500 | 0.018 |
| miR-212 | 1447456_x_at | Dcpp3 | -0.500 | 0.018 |
| miR-212 | 1457041_at | NA | 0.500 | 0.018 |
| miR-212 | 1460198_a_at | Psmb3 | 0.499 | 0.018 |
| miR-212 | 1435222_at | Foxp1 | 0.499 | 0.018 |
| miR-212 | 1427428_at | Clec4g | -0.499 | 0.018 |
| miR-212 | 1435898_x_at | Gdi2 | 0.498 | 0.018 |
| miR-212 | 1452044_at | Arpc5l | 0.498 | 0.018 |
| miR-212 | 1460377_a_at | Tmem8 | -0.498 | 0.018 |
| miR-212 | 1416726_s_at | LOC100048381 /// Ube2s | -0.498 | 0.018 |
| miR-212 | 1453146_at | Gapvd1 | -0.498 | 0.018 |
| miR-212 | 1435873_a_at | Rpl13a /// Zfp526 | 0.498 | 0.018 |
| miR-212 | 1452946_a_at | Rftn2 | 0.498 | 0.018 |
| miR-212 | 1434157_at | Txlna | -0.497 | 0.019 |
| miR-212 | 1452113_a_at | Rab23 | 0.497 | 0.019 |
| miR-212 | 1435220_s_at | Cdc42se2 | 0.497 | 0.019 |
| miR-212 | 1423185_a_at | Ubap1 | -0.497 | 0.019 |
| miR-212 | 1452402_at | NA | 0.497 | 0.019 |
| miR-212 | 1450964_a_at | Osbpl9 | 0.496 | 0.019 |
| miR-212 | 1440392_at | Akap13 | -0.496 | 0.019 |
| miR-212 | 1437237_x_at | Zfp110 | 0.496 | 0.019 |
| miR-212 | 1434274_at | Mycbp2 | 0.496 | 0.019 |
| miR-212 | 1434394_at | N4bp2 | 0.496 | 0.019 |
| miR-212 | 1429383_at | Csnk1g3 /// LOC100047516 | 0.495 | 0.019 |
| miR-212 | 1418843_at | Slc30a4 | 0.495 | 0.019 |
| miR-212 | 1415794_a_at | LOC100046080 /// Spin1 | 0.495 | 0.019 |
| miR-212 | 1434245_a_at | Cybasc3 | 0.495 | 0.019 |
| miR-212 | 1448623_at | Tmem123 | 0.494 | 0.019 |
| miR-212 | 1428919_at | Fgfr1op | 0.494 | 0.019 |
| miR-212 | 1450026_a_at | B3gnt2 | 0.493 | 0.020 |
| miR-212 | 1428655_at | Ccdc128 | 0.493 | 0.020 |
| miR-212 | 1460419_a_at | Prkcb1 | 0.493 | 0.020 |
| miR-212 | 1424780_a_at | Reep3 | 0.493 | 0.020 |
| miR-212 | 1419451_at | Fzr1 | -0.493 | 0.020 |
| miR-212 | 1438655_a_at | ENSMUSG00000068396 /// LOC100040611 /// LOC100043876 /// Rpl34 | 0.493 | 0.020 |
| miR-212 | 1423107_at | Ube2b | -0.492 | 0.020 |
| miR-212 | 1417969_at | Fbxo31 | -0.492 | 0.020 |
| miR-212 | 1426900_at | Jmjd1c | 0.492 | 0.020 |
| miR-212 | 1454897_at | 6330509M05Rik | 0.492 | 0.020 |
| miR-212 | 1460555_at | 6330500D04Rik | 0.492 | 0.020 |
| miR-212 | 1455597_at | Map3k2 | 0.491 | 0.020 |
| miR-212 | 1436515_at | Bach2 | 0.491 | 0.020 |
| miR-212 | 1428301_at | ENSMUSG00000063277 /// ENSMUSG00000068790 /// ENSMUSG00000072735 /// LOC100036568 /// LOC100041195 /// LOC100041874 /// LOC544988 /// LOC666442 /// LOC666637 /// LOC671957 | 0.491 | 0.020 |
| miR-212 | 1416087_at | Ap1s1 | 0.491 | 0.020 |
| miR-212 | 1417007_a_at | Vps4b | 0.491 | 0.020 |
| miR-212 | 1435106_at | Limch1 | 0.490 | 0.021 |
| miR-212 | 1438788_at | D5Wsu152e | 0.490 | 0.021 |
| miR-212 | 1460655_a_at | Ubp1 | -0.490 | 0.021 |
| miR-212 | 1438032_at | Lrch1 | 0.490 | 0.021 |
| miR-212 | 1451974_at | Osbpl2 | 0.490 | 0.021 |
| miR-212 | 1454853_s_at | AI987944 /// AW146154 | 0.490 | 0.021 |
| miR-212 | 1448243_at | Napa | -0.489 | 0.021 |
| miR-212 | 1418016_at | Pum2 | 0.489 | 0.021 |
| miR-212 | 1438887_a_at | Gmcl1 | 0.489 | 0.021 |
| miR-212 | 1434331_at | Eif2c1 | 0.489 | 0.021 |
| miR-212 | 1433351_at | 4933412L11Rik | -0.489 | 0.021 |
| miR-212 | 1427032_at | Herc4 | 0.489 | 0.021 |
| miR-212 | 1420815_at | Gdi2 | 0.488 | 0.021 |
| miR-212 | 1426894_s_at | C230093N12Rik | 0.488 | 0.021 |
| miR-212 | 1455710_x_at | Mtcp1 | -0.488 | 0.021 |
| miR-212 | 1450919_at | Mpp1 | -0.488 | 0.021 |
| miR-212 | 1437667_a_at | Bach2 | 0.488 | 0.021 |
| miR-212 | 1415682_at | Xpo7 | -0.488 | 0.021 |
| miR-212 | 1434035_at | Dnajb6 /// LOC100048324 | -0.487 | 0.021 |
| miR-212 | 1423314_s_at | Pde7a | 0.487 | 0.022 |
| miR-212 | 1431095_a_at | Herc5 | -0.487 | 0.022 |
| miR-212 | 1418839_at | Glmn | 0.487 | 0.022 |
| miR-212 | 1428521_at | Thap3 | 0.486 | 0.022 |
| miR-212 | 1440497_at | NA | -0.485 | 0.022 |
| miR-212 | 1437226_x_at | Marcksl1 | 0.485 | 0.022 |
| miR-212 | 1440579_at | NA | 0.485 | 0.022 |
| miR-212 | 1450660_at | Pts | 0.485 | 0.022 |
| miR-212 | 1418989_at | Ctse | -0.485 | 0.022 |
| miR-212 | 1425358_at | Riok1 | 0.485 | 0.022 |
| miR-212 | 1455897_x_at | Hmgn1 /// LOC100044391 | 0.485 | 0.022 |
| miR-212 | 1451159_at | Arhgef12 | -0.485 | 0.022 |
| miR-212 | 1427888_a_at | Spna2 | 0.484 | 0.022 |
| miR-212 | 1433747_at | Lnpep | 0.484 | 0.022 |
| miR-212 | 1439485_at | Zfp608 | 0.484 | 0.022 |
| miR-212 | 1437133_x_at | Akr1b3 | 0.484 | 0.022 |
| miR-212 | 1446953_at | NA | 0.484 | 0.023 |
| miR-212 | 1415856_at | Emb | 0.484 | 0.023 |
| miR-212 | 1417307_at | Dmd | 0.484 | 0.023 |
| miR-212 | 1415826_at | Atp6v1h | 0.484 | 0.023 |
| miR-212 | 1450628_at | Slc2a8 | -0.484 | 0.023 |
| miR-212 | 1419096_at | Apom | -0.483 | 0.023 |
| miR-212 | 1434770_at | Iqcb1 | 0.483 | 0.023 |
| miR-212 | 1446537_at | NA | 0.483 | 0.023 |
| miR-212 | 1433741_at | Cd38 | 0.483 | 0.023 |
| miR-212 | 1423167_at | Mobkl3 | 0.483 | 0.023 |
| miR-212 | 1421721_a_at | Arnt | -0.483 | 0.023 |
| miR-212 | 1453865_a_at | Otud5 | -0.483 | 0.023 |
| miR-212 | 1451403_at | BC024868 | -0.482 | 0.023 |
| miR-212 | 1456780_at | NA | -0.482 | 0.023 |
| miR-212 | 1426628_at | Tmem34 | 0.482 | 0.023 |
| miR-212 | 1458085_at | Ubxd5 | 0.482 | 0.023 |
| miR-212 | 1454745_at | Arhgap29 | 0.482 | 0.023 |
| miR-212 | 1456381_x_at | Mcl1 | 0.481 | 0.023 |
| miR-212 | 1443148_at | NA | 0.481 | 0.023 |
| miR-212 | 1423918_at | Rhbdd1 | 0.481 | 0.023 |
| miR-212 | 1433938_at | Trp53bp2 | 0.481 | 0.023 |
| miR-212 | 1452587_at | Actr2 | 0.481 | 0.023 |
| miR-212 | 1426840_at | Ythdf3 | 0.481 | 0.023 |
| miR-212 | 1428108_x_at | Tmcc2 | -0.481 | 0.023 |
| miR-212 | 1436838_x_at | Cotl1 | 0.481 | 0.023 |
| miR-212 | 1445837_at | NA | 0.481 | 0.024 |
| miR-212 | 1451357_at | Mpnd | 0.480 | 0.024 |
| miR-212 | 1427427_at | Ryr3 | 0.480 | 0.024 |
| miR-212 | 1425732_a_at | Mxi1 | -0.480 | 0.024 |
| miR-212 | 1433110_at | 5830474E16Rik | 0.480 | 0.024 |
| miR-212 | 1447757_x_at | Inpp5f | 0.480 | 0.024 |
| miR-212 | 1459133_at | Edem3 | 0.480 | 0.024 |
| miR-212 | 1416668_at | Ttc35 | 0.480 | 0.024 |
| miR-212 | 1435540_at | Irgq | 0.480 | 0.024 |
| miR-212 | 1442182_at | Dnajc19 | 0.480 | 0.024 |
| miR-212 | 1452338_s_at | Itsn1 | -0.480 | 0.024 |
| miR-212 | 1433561_at | Centb2 | 0.480 | 0.024 |
| miR-212 | 1416690_at | Gtpbp2 | -0.480 | 0.024 |
| miR-212 | 1439484_at | 4930431H11Rik | 0.479 | 0.024 |
| miR-212 | 1425893_a_at | Fhit | 0.479 | 0.024 |
| miR-212 | 1433575_at | Sox4 | 0.479 | 0.024 |
| miR-212 | 1455134_at | D730040F13Rik | 0.479 | 0.024 |
| miR-212 | 1439805_at | NA | 0.479 | 0.024 |
| miR-212 | 1434487_at | Mef2d | 0.479 | 0.024 |
| miR-212 | 1456642_x_at | S100a10 | 0.479 | 0.024 |
| miR-212 | 1447830_s_at | Rgs2 | 0.479 | 0.024 |
| miR-212 | 1417490_at | Ctsb | -0.479 | 0.024 |
| miR-212 | 1441843_s_at | 5230400M03Rik | -0.478 | 0.024 |
| miR-212 | 1426907_s_at | Dhx57 | 0.478 | 0.024 |
| miR-212 | 1433794_at | Setx | 0.478 | 0.024 |
| miR-212 | 1435432_at | Centg2 | -0.478 | 0.024 |
| miR-212 | 1433765_at | Ube2o | -0.478 | 0.024 |
| miR-212 | 1452666_a_at | Tmcc2 | -0.478 | 0.024 |
| miR-212 | 1420129_s_at | D10Wsu52e | 0.478 | 0.024 |
| miR-212 | 1431811_a_at | Fbxo34 | -0.478 | 0.025 |
| miR-212 | 1429384_at | Csnk1g3 /// LOC100047516 | 0.478 | 0.025 |
| miR-212 | 1417358_s_at | Sorbs1 | -0.478 | 0.025 |
| miR-212 | 1429690_at | 1300003B13Rik | 0.477 | 0.025 |
| miR-212 | 1423792_a_at | Cmtm6 | 0.477 | 0.025 |
| miR-212 | 1448503_at | Mcl1 | 0.477 | 0.025 |
| miR-212 | 1456581_x_at | Gdi2 | 0.477 | 0.025 |
| miR-212 | 1427334_s_at | 2810474O19Rik | -0.477 | 0.025 |
| miR-212 | 1430887_s_at | 1700112E06Rik | 0.477 | 0.025 |
| miR-212 | 1448641_at | Mbtd1 | 0.477 | 0.025 |
| miR-212 | 1429065_at | 1200009F10Rik | 0.476 | 0.025 |
| miR-212 | 1422659_at | Camk2d | 0.476 | 0.025 |
| miR-212 | 1421604_a_at | Klf3 /// LOC100046855 | -0.476 | 0.025 |
| miR-212 | 1418493_a_at | Snca | -0.475 | 0.025 |
| miR-212 | 1429252_at | 0610010K14Rik | 0.475 | 0.025 |
| miR-212 | 1423252_at | Hdgfrp3 | 0.475 | 0.025 |
| miR-212 | 1439830_at | Map3k5 | 0.475 | 0.026 |
| miR-212 | 1444144_at | NA | 0.475 | 0.026 |
| miR-212 | 1417974_at | Kpna4 | 0.474 | 0.026 |
| miR-212 | 1441228_at | Apold1 | -0.474 | 0.026 |
| miR-212 | 1439467_at | NA | 0.474 | 0.026 |
| miR-212 | 1435769_at | Akap9 | 0.474 | 0.026 |
| miR-212 | 1434330_at | Tbcel | -0.474 | 0.026 |
| miR-212 | 1454764_s_at | Slc38a1 | 0.474 | 0.026 |
| miR-212 | 1420953_at | Add1 | -0.473 | 0.026 |
| miR-212 | 1435295_at | Dopey1 | 0.473 | 0.026 |
| miR-212 | 1443678_at | NA | -0.473 | 0.026 |
| miR-212 | 1427043_s_at | Enox2 | 0.473 | 0.026 |
| miR-212 | 1424069_at | Napg | 0.473 | 0.026 |
| miR-212 | 1417465_at | Fnta /// LOC100046996 | 0.473 | 0.026 |
| miR-212 | 1428512_at | Bhlhb9 | 0.472 | 0.026 |
| miR-212 | 1444521_at | NA | 0.472 | 0.026 |
| miR-212 | 1452372_at | Bsdc1 | -0.472 | 0.027 |
| miR-212 | 1429028_at | Dock11 | 0.471 | 0.027 |
| miR-212 | 1460180_at | Hexb | 0.471 | 0.027 |
| miR-212 | 1441987_at | Mbd5 | 0.471 | 0.027 |
| miR-212 | 1427418_a_at | Hif1a | 0.471 | 0.027 |
| miR-212 | 1426662_at | Cmas | -0.471 | 0.027 |
| miR-212 | 1450409_a_at | 4930570C03Rik | -0.471 | 0.027 |
| miR-212 | 1458414_at | D2Ertd93e | 0.470 | 0.027 |
| miR-212 | 1440300_at | NA | 0.470 | 0.027 |
| miR-212 | 1433631_at | Eif5 | -0.470 | 0.027 |
| miR-212 | 1453162_at | Utp11l | 0.470 | 0.027 |
| miR-212 | 1415779_s_at | Actg1 | 0.470 | 0.027 |
| miR-212 | 1424000_a_at | Rps11 | 0.470 | 0.027 |
| miR-212 | 1436846_x_at | EG546165 /// LOC100039786 /// LOC674211 /// Ywhaq | 0.470 | 0.027 |
| miR-212 | 1449855_s_at | Uchl3 /// Uchl4 | 0.470 | 0.027 |
| miR-212 | 1460337_at | Sh3kbp1 | 0.470 | 0.027 |
| miR-212 | 1420956_at | Apc | 0.470 | 0.027 |
| miR-212 | 1454724_x_at | 5730446C15Rik | 0.470 | 0.027 |
| miR-212 | 1418210_at | Pfn2 | 0.469 | 0.027 |
| miR-212 | 1434568_at | NA | 0.469 | 0.028 |
| miR-212 | 1454920_at | Uhrf2 | -0.469 | 0.028 |
| miR-212 | 1448302_at | Kctd20 | -0.469 | 0.028 |
| miR-212 | 1438024_at | NA | 0.469 | 0.028 |
| miR-212 | 1424637_s_at | Ccdc47 | 0.469 | 0.028 |
| miR-212 | 1454910_at | Rad54l2 | -0.469 | 0.028 |
| miR-212 | 1416629_at | Slc1a5 | -0.468 | 0.028 |
| miR-212 | 1436009_at | Usp30 | -0.468 | 0.028 |
| miR-212 | 1436422_at | BC026590 | 0.468 | 0.028 |
| miR-212 | 1458985_at | Fry | 0.468 | 0.028 |
| miR-212 | 1416943_at | Ube2e1 | 0.468 | 0.028 |
| miR-212 | 1422487_at | LOC100048076 /// Smad4 | 0.468 | 0.028 |
| miR-212 | 1460361_at | 5033414D02Rik | 0.468 | 0.028 |
| miR-212 | 1420842_at | Ptprf | 0.468 | 0.028 |
| miR-212 | 1421891_at | St3gal2 | -0.468 | 0.028 |
| miR-212 | 1436807_x_at | Trim62 | -0.467 | 0.028 |
| miR-212 | 1428530_x_at | EG629116 /// LOC668041 /// LOC677113 /// Rps24 | 0.467 | 0.028 |
| miR-212 | 1448442_a_at | LOC100038973 /// Psma3 | 0.467 | 0.028 |
| miR-212 | 1417200_at | Tmem183a | -0.467 | 0.028 |
| miR-212 | 1426118_a_at | Tomm40 | -0.467 | 0.028 |
| miR-212 | 1437065_at | Zbtb20 | 0.467 | 0.028 |
| miR-212 | 1417321_at | Zcchc7 | 0.467 | 0.029 |
| miR-212 | 1451277_at | Zadh2 | 0.467 | 0.029 |
| miR-212 | 1454859_a_at | Rpl23 | 0.467 | 0.029 |
| miR-212 | 1429568_x_at | Ube2f | -0.467 | 0.029 |
| miR-212 | 1423478_at | Prkcb1 | 0.467 | 0.029 |
| miR-212 | 1456292_a_at | Vim | 0.466 | 0.029 |
| miR-212 | 1415857_at | Emb | 0.466 | 0.029 |
| miR-212 | 1435768_at | Arid4b | 0.466 | 0.029 |
| miR-212 | 1422719_s_at | Nup50 | -0.466 | 0.029 |
| miR-212 | 1423632_at | Gpr146 | -0.466 | 0.029 |
| miR-212 | 1424422_s_at | Flad1 /// Lenep | 0.466 | 0.029 |
| miR-212 | 1446484_at | NA | 0.465 | 0.029 |
| miR-212 | 1452067_at | Naaa | -0.465 | 0.029 |
| miR-212 | 1437864_at | Adipor2 | -0.465 | 0.029 |
| miR-212 | 1426483_at | Prkrir | 0.465 | 0.029 |
| miR-212 | 1440118_at | Slc25a39 | -0.465 | 0.029 |
| miR-212 | 1436058_at | Rsad2 | -0.465 | 0.029 |
| miR-212 | 1434881_s_at | Kctd12 | 0.465 | 0.029 |
| miR-212 | 1416403_at | Abcb10 | -0.465 | 0.029 |
| miR-212 | 1418631_at | LOC100047093 /// Ube2h | -0.465 | 0.029 |
| miR-212 | 1425114_at | Rbbp6 | 0.465 | 0.029 |
| miR-212 | 1438504_x_at | Tm7sf3 | -0.464 | 0.030 |
| miR-212 | 1438116_x_at | Slc9a3r1 | 0.464 | 0.030 |
| miR-212 | 1458994_at | NA | 0.464 | 0.030 |
| miR-212 | 1416050_a_at | Scarb1 | -0.464 | 0.030 |
| miR-212 | 1442686_at | NA | -0.463 | 0.030 |
| miR-212 | 1429418_at | Cdc14b | 0.463 | 0.030 |
| miR-212 | 1444273_at | AW555355 | 0.463 | 0.030 |
| miR-212 | 1433939_at | Aff3 | 0.463 | 0.030 |
| miR-212 | 1453174_at | 2310076G13Rik | 0.463 | 0.030 |
| miR-212 | 1418659_at | Clock | 0.463 | 0.030 |
| miR-212 | 1448437_a_at | Gtpbp2 | -0.463 | 0.030 |
| miR-212 | 1433488_x_at | Gns | 0.462 | 0.030 |
| miR-212 | 1437527_x_at | Mcl1 | 0.462 | 0.030 |
| miR-212 | 1451980_at | Casd1 | 0.462 | 0.030 |
| miR-212 | 1457265_at | Sfrs17b | 0.462 | 0.030 |
| miR-212 | 1443226_at | 5730470L24Rik | 0.462 | 0.030 |
| miR-212 | 1425120_x_at | 1810023F06Rik | -0.462 | 0.030 |
| miR-212 | 1435174_at | LOC100045795 /// Rsbn1 | 0.462 | 0.030 |
| miR-212 | 1435159_at | 9430038I01Rik | 0.462 | 0.030 |
| miR-212 | 1422497_at | Slc30a5 | 0.462 | 0.031 |
| miR-212 | 1417423_at | Grina | -0.461 | 0.031 |
| miR-212 | 1429392_at | Wdr40a | -0.461 | 0.031 |
| miR-212 | 1418356_at | Mpst | -0.461 | 0.031 |
| miR-212 | 1449936_at | 8430419L09Rik | -0.461 | 0.031 |
| miR-212 | 1417414_at | Sept3 | -0.461 | 0.031 |
| miR-212 | 1424092_at | Epb4.1 | -0.461 | 0.031 |
| miR-212 | 1457528_at | Slc4a7 | 0.461 | 0.031 |
| miR-212 | 1436421_s_at | Arpc5l | 0.461 | 0.031 |
| miR-212 | 1428378_at | Zc3hav1 | -0.461 | 0.031 |
| miR-212 | 1452797_at | Fastkd3 | 0.461 | 0.031 |
| miR-212 | 1416259_at | Pex12 | 0.461 | 0.031 |
| miR-212 | 1439454_x_at | Tm2d2 | 0.460 | 0.031 |
| miR-212 | 1433658_x_at | Pcbp4 | 0.460 | 0.031 |
| miR-212 | 1457367_at | NA | 0.460 | 0.031 |
| miR-212 | 1451075_s_at | Ctdsp2 | 0.460 | 0.031 |
| miR-212 | 1450927_at | Lztr1 | -0.460 | 0.031 |
| miR-212 | 1455159_at | Appl1 | 0.460 | 0.031 |
| miR-212 | 1420811_a_at | Ctnnb1 | 0.460 | 0.031 |
| miR-212 | 1424229_at | Dyrk3 | -0.460 | 0.031 |
| miR-212 | 1440440_at | NA | 0.460 | 0.031 |
| miR-212 | 1426540_at | Endod1 | -0.460 | 0.031 |
| miR-212 | 1427207_s_at | Afg3l2 /// LOC100048880 | -0.460 | 0.031 |
| miR-212 | 1425118_at | Spire2 | -0.459 | 0.031 |
| miR-212 | 1431214_at | LOC433762 | -0.459 | 0.032 |
| miR-212 | 1450755_at | Pafah1b2 | -0.459 | 0.032 |
| miR-212 | 1455319_x_at | LOC100039036 /// LOC100040298 /// Rps8 | 0.459 | 0.032 |
| miR-212 | 1423873_at | Lsm1 | 0.459 | 0.032 |
| miR-212 | 1426961_at | Phf20 | 0.458 | 0.032 |
| miR-212 | 1434789_at | Depdc1b | -0.458 | 0.032 |
| miR-212 | 1420901_a_at | Hk1 | -0.458 | 0.032 |
| miR-212 | 1428644_at | Mgat5 | 0.458 | 0.032 |
| miR-212 | 1450084_s_at | Ivns1abp | 0.458 | 0.032 |
| miR-212 | 1454937_at | B630005N14Rik | 0.458 | 0.032 |
| miR-212 | 1428347_at | Cyfip2 | 0.458 | 0.032 |
| miR-212 | 1434260_at | Fchsd2 | 0.457 | 0.032 |
| miR-212 | 1438291_x_at | LOC100040532 /// LOC100047378 /// Rpl37 | 0.457 | 0.032 |
| miR-212 | 1416279_at | Ap1b1 | -0.457 | 0.032 |
| miR-212 | 1433984_a_at | Mdh2 | 0.457 | 0.032 |
| miR-212 | 1420612_s_at | Ptp4a2 | 0.457 | 0.033 |
| miR-212 | 1439017_x_at | Adipor1 | -0.457 | 0.033 |
| miR-212 | 1439226_at | Dock8 | 0.457 | 0.033 |
| miR-212 | 1429883_at | Actl6a | 0.457 | 0.033 |
| miR-212 | 1417164_at | Dusp10 | 0.457 | 0.033 |
| miR-212 | 1450943_at | Magohb | 0.456 | 0.033 |
| miR-212 | 1417893_at | Sfxn3 | 0.456 | 0.033 |
| miR-212 | 1423824_at | Gpr177 | 0.456 | 0.033 |
| miR-212 | 1451975_at | 2810453I06Rik | -0.456 | 0.033 |
| miR-212 | 1425405_a_at | Adar | -0.456 | 0.033 |
| miR-212 | 1439651_at | NA | 0.456 | 0.033 |
| miR-212 | 1451050_at | Nt5c3 | -0.456 | 0.033 |
| miR-212 | 1428372_at | St5 | -0.456 | 0.033 |
| miR-212 | 1433661_at | Nlrx1 | -0.456 | 0.033 |
| miR-212 | 1430327_at | mCG_13386 | -0.456 | 0.033 |
| miR-212 | 1450434_s_at | Pcyt1a | -0.455 | 0.033 |
| miR-212 | 1429907_at | 1700094D03Rik | 0.455 | 0.033 |
| miR-212 | 1417981_at | Insig2 | 0.455 | 0.033 |
| miR-212 | 1417102_a_at | LOC100046199 /// Ndufb5 | 0.455 | 0.033 |
| miR-212 | 1422495_a_at | Hmgn1 /// LOC100044391 | 0.455 | 0.033 |
| miR-212 | 1416070_a_at | Ddx18 | 0.455 | 0.033 |
| miR-212 | 1447778_x_at | Brcc3 | -0.455 | 0.033 |
| miR-212 | 1459838_s_at | Btbd11 | 0.455 | 0.034 |
| miR-212 | 1428309_s_at | Pdrg1 | 0.454 | 0.034 |
| miR-212 | 1449056_at | E330009J07Rik | 0.454 | 0.034 |
| miR-212 | 1451274_at | Ogdh | -0.454 | 0.034 |
| miR-212 | 1426898_at | Map3k7ip1 | -0.454 | 0.034 |
| miR-212 | 1420843_at | Ptprf | 0.454 | 0.034 |
| miR-212 | 1418025_at | Bhlhb2 | 0.454 | 0.034 |
| miR-212 | 1417592_at | Frap1 | 0.454 | 0.034 |
| miR-212 | 1417930_at | Nab2 | 0.454 | 0.034 |
| miR-212 | 1418627_at | Gclm | -0.453 | 0.034 |
| miR-212 | 1420124_s_at | Tcta | 0.453 | 0.034 |
| miR-212 | 1435695_a_at | A030007L17Rik | 0.453 | 0.034 |
| miR-212 | 1434654_at | Cog3 | 0.453 | 0.034 |
| miR-212 | 1451675_a_at | Alas2 | -0.453 | 0.034 |
| miR-212 | 1449173_at | Mpp2 | -0.453 | 0.034 |
| miR-212 | 1457639_at | Atp6v1h | 0.453 | 0.034 |
| miR-212 | 1453852_at | Ddx50 | 0.453 | 0.034 |
| miR-212 | 1426985_s_at | 2810485I05Rik | 0.453 | 0.034 |
| miR-212 | 1417805_at | Xpnpep2 | -0.453 | 0.034 |
| miR-212 | 1447090_s_at | Arl1 | 0.453 | 0.034 |
| miR-212 | 1448358_s_at | EG666609 /// Snrpg | 0.453 | 0.034 |
| miR-212 | 1429503_at | 2900024C23Rik | 0.453 | 0.034 |
| miR-212 | 1451420_at | Ccdc47 | 0.453 | 0.034 |
| miR-212 | 1455742_x_at | Morf4l1 | 0.453 | 0.034 |
| miR-212 | 1437185_s_at | LOC100043712 /// LOC100047613 /// LOC100048142 /// Tmsb10 | 0.452 | 0.035 |
| miR-212 | 1449530_at | LOC100044341 /// Trps1 | 0.452 | 0.035 |
| miR-212 | 1434625_at | 4930432O21Rik | 0.452 | 0.035 |
| miR-212 | 1434018_at | BC043098 | 0.452 | 0.035 |
| miR-212 | 1434404_at | C030011O14Rik | 0.452 | 0.035 |
| miR-212 | 1442858_at | Mycbp2 | 0.452 | 0.035 |
| miR-212 | 1420628_at | LOC100045958 /// Pura | 0.452 | 0.035 |
| miR-212 | 1429085_at | Vezf1 | 0.452 | 0.035 |
| miR-212 | 1418436_at | Stx7 | 0.452 | 0.035 |
| miR-212 | 1460365_a_at | Dnm1 | -0.452 | 0.035 |
| miR-212 | 1421923_at | Sh3bp5 | 0.451 | 0.035 |
| miR-212 | 1423834_s_at | Gga1 | -0.451 | 0.035 |
| miR-212 | 1417842_at | Caml | 0.451 | 0.035 |
| miR-212 | 1443737_at | NA | -0.451 | 0.035 |
| miR-212 | 1428162_at | 4933421E11Rik | 0.451 | 0.035 |
| miR-212 | 1450539_at | Krtap5-1 | -0.451 | 0.035 |
| miR-212 | 1430075_at | Sf3b3 | 0.451 | 0.035 |
| miR-212 | 1455291_s_at | Znrf2 | 0.451 | 0.035 |
| miR-212 | 1429351_at | Klhl24 | 0.451 | 0.035 |
| miR-212 | 1434108_at | Fbxo11 | 0.450 | 0.035 |
| miR-212 | 1426411_a_at | C230082I21Rik /// Strbp | 0.450 | 0.035 |
| miR-212 | 1435343_at | Dock10 | 0.450 | 0.035 |
| miR-212 | 1423073_at | Cmpk1 | 0.450 | 0.036 |
| miR-212 | 1451092_a_at | Rangap1 | -0.450 | 0.036 |
| miR-212 | 1424059_at | Suv420h2 | -0.450 | 0.036 |
| miR-212 | 1422031_a_at | Zfand6 | -0.450 | 0.036 |
| miR-212 | 1415752_at | BC031181 | -0.450 | 0.036 |
| miR-212 | 1428077_at | LOC100047091 /// Tmem163 | 0.450 | 0.036 |
| miR-212 | 1437748_at | Fut11 | -0.450 | 0.036 |
| miR-212 | 1417584_at | Slc11a2 | -0.450 | 0.036 |
| miR-212 | 1422095_a_at | Cmpk2 | -0.449 | 0.036 |
| miR-212 | 1420888_at | Bcl2l1 | -0.449 | 0.036 |
| miR-212 | 1419551_s_at | Stk39 | -0.449 | 0.036 |
| miR-212 | 1423962_at | Wdr26 | -0.449 | 0.036 |
| miR-212 | 1421242_at | Rnf144a | -0.449 | 0.036 |
| miR-212 | 1430367_at | Stambpl1 | 0.449 | 0.036 |
| miR-212 | 1434743_x_at | Rusc1 | 0.449 | 0.036 |
| miR-212 | 1426532_at | Zmynd11 | 0.449 | 0.036 |
| miR-212 | 1416315_at | Abhd4 | -0.449 | 0.036 |
| miR-212 | 1460276_a_at | Gpr175 | -0.449 | 0.036 |
| miR-212 | 1457248_x_at | Hsd17b7 | 0.449 | 0.036 |
| miR-212 | 1424828_a_at | Fh1 | 0.449 | 0.036 |
| miR-212 | 1424584_a_at | Ranbp10 | -0.448 | 0.036 |
| miR-212 | 1444575_at | NA | 0.448 | 0.036 |
| miR-212 | 1424002_at | Pdcl3 | 0.448 | 0.036 |
| miR-212 | 1438454_at | B430203M17Rik | 0.448 | 0.036 |
| miR-212 | 1427120_at | Zfp26 | 0.448 | 0.036 |
| miR-212 | 1435843_x_at | Mrps9 | 0.448 | 0.036 |
| miR-212 | 1429059_s_at | Tmem107 | -0.448 | 0.037 |
| miR-212 | 1424769_s_at | Cald1 | 0.448 | 0.037 |
| miR-212 | 1435662_at | Nkap | 0.448 | 0.037 |
| miR-212 | 1454641_at | Cggbp1 | 0.448 | 0.037 |
| miR-212 | 1453864_at | Rdh14 | 0.448 | 0.037 |
| miR-212 | 1416464_at | Slc4a1 | -0.447 | 0.037 |
| miR-212 | 1422490_at | Bnip2 | 0.447 | 0.037 |
| miR-212 | 1450023_at | Gtpbp1 | -0.447 | 0.037 |
| miR-212 | 1453362_x_at | EG629116 /// LOC677113 /// Rps24 | 0.447 | 0.037 |
| miR-212 | 1427425_at | 9130208E07Rik | -0.447 | 0.037 |
| miR-212 | 1444811_at | Sec62 | -0.447 | 0.037 |
| miR-212 | 1429497_s_at | Snx6 | 0.447 | 0.037 |
| miR-212 | 1415780_a_at | Armcx2 | 0.447 | 0.037 |
| miR-212 | 1457498_at | Man2a2 | 0.447 | 0.037 |
| miR-212 | 1422473_at | Pde4b | 0.447 | 0.037 |
| miR-212 | 1437852_x_at | Cpsf3 | 0.447 | 0.037 |
| miR-212 | 1424071_s_at | BC018507 | 0.447 | 0.037 |
| miR-212 | 1439599_at | Gal3st2 | 0.446 | 0.037 |
| miR-212 | 1429360_at | Klf3 /// LOC100046855 | -0.446 | 0.037 |
| miR-212 | 1456547_at | NA | 0.446 | 0.037 |
| miR-212 | 1454236_a_at | Ppp4r1l | 0.446 | 0.037 |
| miR-212 | 1435780_at | Psd | -0.446 | 0.038 |
| miR-212 | 1451154_a_at | Cugbp2 | 0.446 | 0.038 |
| miR-212 | 1436015_s_at | Stk4 | 0.446 | 0.038 |
| miR-212 | 1451782_a_at | Slc29a1 | -0.446 | 0.038 |
| miR-212 | 1450889_at | Hltf | 0.446 | 0.038 |
| miR-212 | 1454889_x_at | Tmcc3 | 0.446 | 0.038 |
| miR-212 | 1421397_a_at | Lrdd | -0.445 | 0.038 |
| miR-212 | 1422595_s_at | 5730470L24Rik | 0.445 | 0.038 |
| miR-212 | 1454791_a_at | Rbbp4 | -0.445 | 0.038 |
| miR-212 | 1434054_at | Mafg | -0.445 | 0.038 |
| miR-212 | 1429081_at | Gcc2 | 0.445 | 0.038 |
| miR-212 | 1423129_at | Shoc2 | 0.445 | 0.038 |
| miR-212 | 1417480_at | Fbxo9 | -0.445 | 0.038 |
| miR-212 | 1424294_at | Ppp4r1 | 0.445 | 0.038 |
| miR-212 | 1455642_a_at | Tspan17 | 0.445 | 0.038 |
| miR-212 | 1455141_at | Tnrc6a | 0.445 | 0.038 |
| miR-212 | 1456922_at | Snx29 | 0.444 | 0.038 |
| miR-212 | 1436970_a_at | Pdgfrb | 0.444 | 0.038 |
| miR-212 | 1424902_at | Plxdc1 | -0.444 | 0.038 |
| miR-212 | 1452258_at | Phf20 | 0.444 | 0.039 |
| miR-212 | 1453422_a_at | 1110020G09Rik | -0.444 | 0.039 |
| miR-212 | 1436949_a_at | LOC638798 /// LOC665250 /// LOC677628 /// Tceb2 | 0.444 | 0.039 |
| miR-212 | 1438115_a_at | Slc9a3r1 | 0.443 | 0.039 |
| miR-212 | 1437908_a_at | Ergic1 | 0.443 | 0.039 |
| miR-212 | 1448639_a_at | Spata5 | -0.443 | 0.039 |
| miR-212 | 1435959_at | Arhgap15 | 0.443 | 0.039 |
| miR-212 | 1459914_at | Mcart6 | 0.443 | 0.039 |
| miR-212 | 1422506_a_at | Cstb | 0.443 | 0.039 |
| miR-212 | 1419945_s_at | Rab2a | 0.442 | 0.039 |
| miR-212 | 1417000_at | Abtb1 | -0.442 | 0.039 |
| miR-212 | 1437467_at | Alcam | 0.442 | 0.039 |
| miR-212 | 1442122_at | AI451458 | 0.442 | 0.040 |
| miR-212 | 1426160_a_at | Stk16 | -0.441 | 0.040 |
| miR-212 | 1435808_at | A230051G13Rik | -0.441 | 0.040 |
| miR-212 | 1445801_at | NA | -0.441 | 0.040 |
| miR-212 | 1437391_x_at | Mrpl44 | 0.441 | 0.040 |
| miR-212 | 1431020_a_at | Fgfr1op2 | -0.441 | 0.040 |
| miR-212 | 1448580_at | Glg1 | -0.441 | 0.040 |
| miR-212 | 1417008_at | Crat | -0.441 | 0.040 |
| miR-212 | 1425119_at | Oas1b | -0.441 | 0.040 |
| miR-212 | 1428343_at | Rcor3 | -0.441 | 0.040 |
| miR-212 | 1416359_at | Snx18 | 0.441 | 0.040 |
| miR-212 | 1438062_at | 4832420A03Rik /// Rsf1 | 0.441 | 0.040 |
| miR-212 | 1447150_at | Mycbp2 | 0.441 | 0.040 |
| miR-212 | 1427927_at | Hscb | -0.441 | 0.040 |
| miR-212 | 1424536_at | Oas1e | -0.440 | 0.040 |
| miR-212 | 1416880_at | Mcl1 | 0.440 | 0.040 |
| miR-212 | 1430133_at | Tbc1d8b | 0.440 | 0.040 |
| miR-212 | 1422855_at | Cpsf3 | 0.440 | 0.040 |
| miR-212 | 1436208_at | Asb1 | -0.440 | 0.040 |
| miR-212 | 1444100_at | NA | 0.440 | 0.040 |
| miR-212 | 1435017_at | Mel13 | 0.440 | 0.040 |
| miR-212 | 1451936_a_at | Txnrd2 | -0.440 | 0.040 |
| miR-212 | 1421000_at | Cnot4 | 0.440 | 0.040 |
| miR-212 | 1428330_at | Dopey2 | -0.440 | 0.040 |
| miR-212 | 1427113_s_at | Ttl | 0.440 | 0.041 |
| miR-212 | 1433482_a_at | Fubp1 | 0.440 | 0.041 |
| miR-212 | 1455269_a_at | Coro1a | 0.440 | 0.041 |
| miR-212 | 1439429_x_at | Dtx2 | 0.440 | 0.041 |
| miR-212 | 1429155_at | 4933411K20Rik | 0.440 | 0.041 |
| miR-212 | 1418194_at | Galnt10 | -0.440 | 0.041 |
| miR-212 | 1426726_at | LOC677319 /// Ppp1r10 | -0.440 | 0.041 |
| miR-212 | 1433993_at | 4931406P16Rik | -0.440 | 0.041 |
| miR-212 | 1448129_at | Arpc5 | 0.439 | 0.041 |
| miR-212 | 1455403_at | Manea | 0.439 | 0.041 |
| miR-212 | 1426947_x_at | Col6a2 | -0.439 | 0.041 |
| miR-212 | 1418699_s_at | Fech | -0.439 | 0.041 |
| miR-212 | 1419069_at | Rabgef1 | -0.439 | 0.041 |
| miR-212 | 1447711_x_at | 4933412E12Rik | 0.439 | 0.041 |
| miR-212 | 1435284_at | Rtn4 | 0.439 | 0.041 |
| miR-212 | 1426716_at | Tdrd7 | -0.439 | 0.041 |
| miR-212 | 1457610_at | NA | -0.439 | 0.041 |
| miR-212 | 1423612_at | Clp1 | -0.439 | 0.041 |
| miR-212 | 1447450_at | Med1 | 0.439 | 0.041 |
| miR-212 | 1433770_at | Dpysl2 | 0.439 | 0.041 |
| miR-212 | 1427091_at | Znfx1 | -0.439 | 0.041 |
| miR-212 | 1459511_at | NA | -0.439 | 0.041 |
| miR-212 | 1453032_at | Mobkl3 | 0.438 | 0.041 |
| miR-212 | 1436971_x_at | Ywhaz | 0.438 | 0.041 |
| miR-212 | 1417575_at | Otub2 | -0.438 | 0.041 |
| miR-212 | 1448370_at | Ulk1 | -0.438 | 0.042 |
| miR-212 | 1440535_at | Uros | -0.438 | 0.042 |
| miR-212 | 1455518_at | NA | 0.438 | 0.042 |
| miR-212 | 1452970_at | Zmym2 | 0.438 | 0.042 |
| miR-212 | 1455323_at | Rbak | 0.437 | 0.042 |
| miR-212 | 1437202_at | Mysm1 | 0.437 | 0.042 |
| miR-212 | 1428156_at | Gng2 | 0.437 | 0.042 |
| miR-212 | 1425776_a_at | C87436 | 0.437 | 0.042 |
| miR-212 | 1455483_at | Zfp148 | 0.437 | 0.042 |
| miR-212 | 1448325_at | Myd116 | -0.437 | 0.042 |
| miR-212 | 1448863_a_at | Tnfaip1 | 0.436 | 0.042 |
| miR-212 | 1456279_a_at | Bcap31 | 0.436 | 0.042 |
| miR-212 | 1451601_a_at | Spns2 | 0.436 | 0.043 |
| miR-212 | 1438631_x_at | Ttc13 | 0.436 | 0.043 |
| miR-212 | 1434830_at | Mxd1 | -0.436 | 0.043 |
| miR-212 | 1418710_at | Cd59a | -0.435 | 0.043 |
| miR-212 | 1426721_s_at | Tiparp | 0.435 | 0.043 |
| miR-212 | 1423963_at | Wdr26 | -0.435 | 0.043 |
| miR-212 | 1428871_at | 4121402D02Rik /// LOC100047441 | 0.435 | 0.043 |
| miR-212 | 1427983_at | Zfp280c | 0.435 | 0.043 |
| miR-212 | 1460436_at | Ndst1 | 0.435 | 0.043 |
| miR-212 | 1424781_at | Reep3 | 0.435 | 0.043 |
| miR-212 | 1434841_at | A430093A21Rik | 0.435 | 0.043 |
| miR-212 | 1436339_at | 1810058I24Rik | -0.435 | 0.043 |
| miR-212 | 1418616_at | Mafk | -0.435 | 0.043 |
| miR-212 | 1448916_at | LOC100047868 /// Mafg | -0.434 | 0.043 |
| miR-212 | 1424324_at | Esco1 | 0.434 | 0.043 |
| miR-212 | 1449341_a_at | Stom | -0.434 | 0.043 |
| miR-212 | 1438173_x_at | Pmf1 | -0.434 | 0.043 |
| miR-212 | 1417398_at | Rras2 | 0.434 | 0.044 |
| miR-212 | 1452759_s_at | Ppfibp1 | 0.434 | 0.044 |
| miR-212 | 1436997_x_at | Sh3bgrl | 0.433 | 0.044 |
| miR-212 | 1452822_at | Ints7 | 0.433 | 0.044 |
| miR-212 | 1424585_at | Ranbp10 | -0.433 | 0.044 |
| miR-212 | 1428229_at | Prkd3 | 0.433 | 0.044 |
| miR-212 | 1435556_at | Zfp597 | 0.433 | 0.044 |
| miR-212 | 1435807_at | Cdc42 /// LOC664922 | 0.433 | 0.044 |
| miR-212 | 1455738_at | Ccdc55 | 0.433 | 0.044 |
| miR-212 | 1437747_at | Ube4a | -0.433 | 0.044 |
| miR-212 | 1453261_at | 2610035D17Rik | 0.433 | 0.044 |
| miR-212 | 1448715_x_at | Ccrn4l /// Cog6 /// ENSMUSG00000073624 /// LOC100043821 /// Sgip1 | -0.433 | 0.044 |
| miR-212 | 1417228_at | Capn1 | -0.433 | 0.044 |
| miR-212 | 1451212_at | Ccdc21 | -0.433 | 0.044 |
| miR-212 | 1419164_at | Zfp260 | 0.433 | 0.044 |
| miR-212 | 1439328_at | Nfat5 | 0.433 | 0.044 |
| miR-212 | 1423961_at | Wdr26 | -0.433 | 0.044 |
| miR-212 | 1431066_at | Fut11 | -0.433 | 0.044 |
| miR-212 | 1439246_x_at | Tnrc6a | 0.433 | 0.044 |
| miR-212 | 1455700_at | Mterfd3 | 0.432 | 0.044 |
| miR-212 | 1446670_at | NA | 0.432 | 0.044 |
| miR-212 | 1448896_at | Pigf | 0.432 | 0.044 |
| miR-212 | 1434187_at | Alg11 | 0.432 | 0.044 |
| miR-212 | 1420631_a_at | Blcap | 0.432 | 0.045 |
| miR-212 | 1434402_at | Samd8 | 0.432 | 0.045 |
| miR-212 | 1417786_a_at | Rgs19 | 0.432 | 0.045 |
| miR-212 | 1434773_a_at | Slc2a1 | 0.432 | 0.045 |
| miR-212 | 1442098_at | AU022434 | 0.432 | 0.045 |
| miR-212 | 1422512_a_at | Ogfr | -0.432 | 0.045 |
| miR-212 | 1452164_at | BC038286 | -0.432 | 0.045 |
| miR-212 | 1416982_at | Foxo1 | 0.431 | 0.045 |
| miR-212 | 1440609_at | Map4k4 | 0.431 | 0.045 |
| miR-212 | 1440795_x_at | Rabep2 | 0.431 | 0.045 |
| miR-212 | 1435885_s_at | Itsn1 | -0.431 | 0.045 |
| miR-212 | 1436809_a_at | LOC100046080 /// Spin1 | 0.431 | 0.045 |
| miR-212 | 1436617_at | Cetn4 | -0.431 | 0.045 |
| miR-212 | 1459621_at | Itsn1 | -0.430 | 0.046 |
| miR-212 | 1428171_at | Prpf39 | 0.430 | 0.046 |
| miR-212 | 1454896_at | Rbpj | 0.430 | 0.046 |
| miR-212 | 1452291_at | Centd1 | 0.430 | 0.046 |
| miR-212 | 1424775_at | Oas1a | -0.430 | 0.046 |
| miR-212 | 1456243_x_at | Mcl1 | 0.430 | 0.046 |
| miR-212 | 1423978_at | Sbk1 | 0.430 | 0.046 |
| miR-212 | 1429830_a_at | Cd59a | -0.430 | 0.046 |
| miR-212 | 1434378_a_at | NA | 0.430 | 0.046 |
| miR-212 | 1454755_at | Itpkc | 0.430 | 0.046 |
| miR-212 | 1447904_s_at | Fnta /// LOC100046996 | 0.430 | 0.046 |
| miR-212 | 1438647_x_at | Cetn2 | 0.430 | 0.046 |
| miR-212 | 1434295_at | Rasgrp1 | 0.430 | 0.046 |
| miR-212 | 1420596_at | Cacng2 | -0.429 | 0.046 |
| miR-212 | 1418644_a_at | Stk11 | -0.429 | 0.046 |
| miR-212 | 1439548_at | Rap2b | 0.429 | 0.046 |
| miR-212 | 1438853_x_at | Ddx54 | 0.429 | 0.046 |
| miR-212 | 1438630_x_at | Mat2a | 0.429 | 0.047 |
| miR-212 | 1459871_x_at | Mar2 | -0.429 | 0.047 |
| miR-212 | 1433588_at | D6Wsu116e | 0.429 | 0.047 |
| miR-212 | 1444051_at | 1700019D03Rik | 0.428 | 0.047 |
| miR-212 | 1456430_at | Ttc14 | 0.428 | 0.047 |
| miR-212 | 1456286_at | NA | 0.428 | 0.047 |
| miR-212 | 1433999_at | Slk | -0.428 | 0.047 |
| miR-212 | 1435868_at | Ankrd13c | -0.428 | 0.047 |
| miR-212 | 1435368_a_at | Parp1 | 0.428 | 0.047 |
| miR-212 | 1441823_at | Zmiz1 | 0.428 | 0.047 |
| miR-212 | 1429389_at | Setmar | 0.428 | 0.047 |
| miR-212 | 1452093_at | Tmem185b | 0.428 | 0.047 |
| miR-212 | 1433574_at | Cdc37l1 | 0.428 | 0.047 |
| miR-212 | 1429194_at | Tigd2 | 0.428 | 0.047 |
| miR-212 | 1418183_a_at | Pscd1 | 0.428 | 0.047 |
| miR-212 | 1423652_at | Isca1 | -0.428 | 0.047 |
| miR-212 | 1421140_a_at | Foxp1 | 0.428 | 0.047 |
| miR-212 | 1417980_a_at | Insig2 | 0.428 | 0.047 |
| miR-212 | 1426780_at | D14Ertd436e | -0.427 | 0.047 |
| miR-212 | 1455316_x_at | ENSMUSG00000073624 | -0.427 | 0.047 |
| miR-212 | 1438118_x_at | Vim | 0.427 | 0.047 |
| miR-212 | 1416185_a_at | Adh5 | 0.427 | 0.047 |
| miR-212 | 1416472_at | Syap1 | 0.427 | 0.047 |
| miR-212 | 1456879_at | C130022K22Rik | 0.427 | 0.047 |
| miR-212 | 1429202_at | 2610019N06Rik | 0.427 | 0.047 |
| miR-212 | 1439380_x_at | Meg3 | -0.427 | 0.047 |
| miR-212 | 1416981_at | Foxo1 | 0.427 | 0.048 |
| miR-212 | 1434042_s_at | Mtmr3 | -0.427 | 0.048 |
| miR-212 | 1417288_at | Plekha2 | 0.427 | 0.048 |
| miR-212 | 1436034_at | Cep68 | 0.427 | 0.048 |
| miR-212 | 1434461_at | Zfp715 | 0.427 | 0.048 |
| miR-212 | 1424683_at | 1810015C04Rik | 0.427 | 0.048 |
| miR-212 | 1458185_at | Thap4 | 0.427 | 0.048 |
| miR-212 | 1455723_at | D1Ertd448e | 0.427 | 0.048 |
| miR-212 | 1446720_at | NA | 0.426 | 0.048 |
| miR-212 | 1420810_at | 1500003O03Rik /// LOC100048622 | -0.426 | 0.048 |
| miR-212 | 1418835_at | Phlda1 | 0.426 | 0.048 |
| miR-212 | 1447818_x_at | Rhebl1 | 0.426 | 0.048 |
| miR-212 | 1453164_a_at | Ptdss2 | -0.426 | 0.048 |
| miR-212 | 1441948_x_at | Zfand3 | 0.426 | 0.048 |
| miR-212 | 1452988_at | 2610306M01Rik | 0.426 | 0.048 |
| miR-212 | 1415827_a_at | D3Ucla1 | 0.426 | 0.048 |
| miR-212 | 1460204_at | Tec | 0.426 | 0.048 |
| miR-212 | 1418592_at | Dnaja4 | -0.426 | 0.048 |
| miR-212 | 1417128_at | Plekho1 | 0.426 | 0.048 |
| miR-212 | 1424888_at | LOC100044703 /// March2 | -0.426 | 0.048 |
| miR-212 | 1441117_at | NA | 0.426 | 0.048 |
| miR-212 | 1455317_at | Epc2 | 0.426 | 0.048 |
| miR-212 | 1453266_at | Zbtb4 | 0.426 | 0.048 |
| miR-212 | 1457851_at | D10Ertd276e | -0.426 | 0.048 |
| miR-212 | 1422272_at | Phxr4 | 0.426 | 0.048 |
| miR-212 | 1415979_x_at | Rpl7 | 0.426 | 0.048 |
| miR-212 | 1431334_a_at | 4933433P14Rik | 0.426 | 0.048 |
| miR-212 | 1419184_a_at | Fhl2 | -0.426 | 0.048 |
| miR-212 | 1436990_s_at | Ndg2 | 0.425 | 0.049 |
| miR-212 | 1435745_at | 5031439G07Rik | 0.425 | 0.049 |
| miR-212 | 1444493_at | NA | -0.425 | 0.049 |
| miR-212 | 1433613_at | Pank3 | 0.425 | 0.049 |
| miR-212 | 1439460_a_at | Arfgap2 | 0.425 | 0.049 |
| miR-212 | 1456209_x_at | NA | 0.425 | 0.049 |
| miR-212 | 1425480_at | Cnot6l | 0.425 | 0.049 |
| miR-212 | 1447679_s_at | Bms1 | 0.425 | 0.049 |
| miR-212 | 1431364_a_at | 2810047C21Rik /// EG665577 | -0.425 | 0.049 |
| miR-212 | 1441266_at | Strn3 | 0.425 | 0.049 |
| miR-212 | 1431372_at | Srpk2 | 0.425 | 0.049 |
| miR-212 | 1437974_a_at | Hk1 | -0.425 | 0.049 |
| miR-212 | 1428806_at | Csnk1g1 | 0.424 | 0.049 |
| miR-212 | 1450643_s_at | Acsl1 | -0.424 | 0.049 |
| miR-212 | 1451437_at | Zdhhc20 | 0.424 | 0.049 |
| miR-212 | 1440876_at | NA | 0.424 | 0.049 |
| miR-212 | 1429393_at | Wdr40a | -0.424 | 0.049 |
| miR-212 | 1457559_at | NA | 0.424 | 0.049 |
| miR-212 | 1442155_at | 4632427E13Rik | 0.424 | 0.049 |
| miR-212 | 1437621_x_at | EG668771 /// LOC665516 /// LOC675316 /// Phgdh | 0.424 | 0.049 |
| miR-212 | 1415793_at | Pnpo | -0.424 | 0.049 |
| miR-212 | 1443772_at | Dzip1 | 0.424 | 0.049 |
| miR-212 | 1436714_at | Lpp | 0.424 | 0.049 |
| miR-212 | 1417426_at | Srgn | 0.424 | 0.049 |
| miR-212 | 1416892_s_at | 3110001A13Rik | 0.424 | 0.049 |
| miR-212 | 1425929_a_at | Rnf14 | -0.423 | 0.050 |
| miR-212 | 1424374_at | Gimap4 | 0.423 | 0.050 |
| miR-212 | 1435754_at | Zyg11b | 0.423 | 0.050 |
| miR-212 | 1435755_at | 1110001A16Rik | 0.423 | 0.050 |
| miR-212 | 1455912_x_at | Unc45a | 0.423 | 0.050 |
| miR-212 | 1437993_x_at | Qdpr | 0.423 | 0.050 |
| miR-301a | 1415773_at | Ncl | 0.714 | 0.000 |
| miR-301a | 1460438_at | LOC100044319 /// Lysmd1 | -0.706 | 0.000 |
| miR-301a | 1425780_a_at | Tmem167 | 0.684 | 0.000 |
| miR-301a | 1427896_at | Suds3 | -0.683 | 0.000 |
| miR-301a | 1452102_at | Copb2 | 0.681 | 0.000 |
| miR-301a | 1452787_a_at | Prmt1 | 0.680 | 0.000 |
| miR-301a | 1424018_at | Hint1 | 0.676 | 0.001 |
| miR-301a | 1430503_at | 6330522J23Rik | 0.675 | 0.001 |
| miR-301a | 1455922_at | Rab3gap1 | -0.670 | 0.001 |
| miR-301a | 1451026_at | Ftsj3 | 0.647 | 0.001 |
| miR-301a | 1432416_a_at | Npm1 | 0.647 | 0.001 |
| miR-301a | 1423040_at | Bzw1 | 0.643 | 0.001 |
| miR-301a | 1456383_at | Rsl1d1 | 0.641 | 0.001 |
| miR-301a | 1416368_at | Gsta4 | 0.636 | 0.001 |
| miR-301a | 1415817_s_at | Cct7 | 0.633 | 0.002 |
| miR-301a | 1448869_a_at | Mrps16 | 0.631 | 0.002 |
| miR-301a | 1451120_at | LOC100039220 /// Rpo1-3 | 0.629 | 0.002 |
| miR-301a | 1431932_s_at | Trim44 | 0.628 | 0.002 |
| miR-301a | 1451025_at | Arl1 | 0.628 | 0.002 |
| miR-301a | 1429116_at | Slc17a5 | 0.627 | 0.002 |
| miR-301a | 1434537_at | Slco3a1 | 0.627 | 0.002 |
| miR-301a | 1450874_at | Matr3 | 0.626 | 0.002 |
| miR-301a | 1431777_a_at | Hmgn3 | -0.626 | 0.002 |
| miR-301a | 1417077_at | Bcap29 | 0.624 | 0.002 |
| miR-301a | 1416143_at | Atp5j /// LOC674583 | 0.622 | 0.002 |
| miR-301a | 1437110_at | 2810474O19Rik | -0.620 | 0.002 |
| miR-301a | 1426701_at | 4632419K20Rik | -0.618 | 0.002 |
| miR-301a | 1448282_at | Plrg1 | 0.615 | 0.002 |
| miR-301a | 1460201_a_at | EG668829 /// Rpl24 | 0.615 | 0.002 |
| miR-301a | 1434031_at | Zfp692 | -0.612 | 0.002 |
| miR-301a | 1444493_at | NA | -0.610 | 0.003 |
| miR-301a | 1444811_at | Sec62 | -0.609 | 0.003 |
| miR-301a | 1428659_at | Phf7 | 0.603 | 0.003 |
| miR-301a | 1426257_a_at | Sars | 0.602 | 0.003 |
| miR-301a | 1416577_a_at | Rbx1 | -0.601 | 0.003 |
| miR-301a | 1439918_at | Odf2 | -0.600 | 0.003 |
| miR-301a | 1448846_a_at | EG666642 /// LOC100039782 /// LOC100044494 /// LOC433941 /// LOC622707 /// LOC665032 /// LOC670211 /// Rpl29 | 0.599 | 0.003 |
| miR-301a | 1457455_at | Suhw4 | -0.599 | 0.003 |
| miR-301a | 1424078_s_at | Pex6 | -0.597 | 0.003 |
| miR-301a | 1434875_a_at | Hmgn3 | -0.597 | 0.003 |
| miR-301a | 1450685_at | Arpp19 | 0.596 | 0.003 |
| miR-301a | 1459657_s_at | LOC100039220 /// Rpo1-3 | 0.595 | 0.003 |
| miR-301a | 1442079_at | Sgms1 | -0.594 | 0.004 |
| miR-301a | 1449137_at | Pdha1 | 0.594 | 0.004 |
| miR-301a | 1449355_a_at | Eps15l1 | -0.593 | 0.004 |
| miR-301a | 1460549_a_at | Cdc23 | 0.592 | 0.004 |
| miR-301a | 1448654_at | Mtch2 | 0.591 | 0.004 |
| miR-301a | 1432488_a_at | Sf3a3 | 0.591 | 0.004 |
| miR-301a | 1426553_at | Dnajc14 | 0.590 | 0.004 |
| miR-301a | 1459564_at | LOC100043819 | -0.590 | 0.004 |
| miR-301a | 1439826_at | Hspa14 | 0.589 | 0.004 |
| miR-301a | 1423109_s_at | Slc25a20 | 0.589 | 0.004 |
| miR-301a | 1455675_a_at | Tial1 | -0.588 | 0.004 |
| miR-301a | 1456040_at | Sf3b2 | 0.586 | 0.004 |
| miR-301a | 1428093_at | Trabd | -0.586 | 0.004 |
| miR-301a | 1456341_a_at | 2310051E17Rik /// Klf9 | -0.586 | 0.004 |
| miR-301a | 1440647_at | Sipa1l1 | -0.583 | 0.004 |
| miR-301a | 1423223_a_at | Prdx6 | 0.582 | 0.004 |
| miR-301a | 1436051_at | Myo5a | -0.582 | 0.004 |
| miR-301a | 1427548_a_at | Clns1a /// ENSMUSG00000056003 /// LOC100040211 | 0.581 | 0.005 |
| miR-301a | 1444168_at | Xpr1 | -0.578 | 0.005 |
| miR-301a | 1417041_at | Rpo1-1 | 0.577 | 0.005 |
| miR-301a | 1442064_at | AW556556 | -0.577 | 0.005 |
| miR-301a | 1452191_at | LOC100048391 /// Prcp | 0.576 | 0.005 |
| miR-301a | 1434525_at | Pkn3 | -0.576 | 0.005 |
| miR-301a | 1459008_at | NA | -0.576 | 0.005 |
| miR-301a | 1442305_at | Gtpbp2 | -0.575 | 0.005 |
| miR-301a | 1452626_a_at | 1810014F10Rik | 0.575 | 0.005 |
| miR-301a | 1443436_at | NA | -0.575 | 0.005 |
| miR-301a | 1428013_at | 6030458C11Rik | -0.575 | 0.005 |
| miR-301a | 1458999_at | Mrps10 | -0.574 | 0.005 |
| miR-301a | 1429515_at | Ubr2 | -0.574 | 0.005 |
| miR-301a | 1418019_at | Cpd | 0.574 | 0.005 |
| miR-301a | 1451366_at | Cops6 | 0.574 | 0.005 |
| miR-301a | 1448430_a_at | Naca | 0.574 | 0.005 |
| miR-301a | 1421324_a_at | Akt2 /// LOC100048123 | 0.572 | 0.005 |
| miR-301a | 1429655_at | Nudcd1 | 0.571 | 0.005 |
| miR-301a | 1439028_at | Ufm1 | -0.571 | 0.005 |
| miR-301a | 1448392_at | Sparc | 0.571 | 0.006 |
| miR-301a | 1436274_at | LOC244958 | 0.570 | 0.006 |
| miR-301a | 1440486_at | 2310037I24Rik | -0.569 | 0.006 |
| miR-301a | 1424412_at | Ogfrl1 | -0.569 | 0.006 |
| miR-301a | 1449643_s_at | Btf3 | 0.569 | 0.006 |
| miR-301a | 1441071_at | Kcnq5 | -0.569 | 0.006 |
| miR-301a | 1421894_a_at | Tpp2 | 0.567 | 0.006 |
| miR-301a | 1423715_a_at | Nedd8 | 0.567 | 0.006 |
| miR-301a | 1422660_at | Rbm3 | 0.566 | 0.006 |
| miR-301a | 1446618_at | LOC432971 | -0.565 | 0.006 |
| miR-301a | AFFX-GapdhMur/M32599_3_at | Gapdh | 0.564 | 0.006 |
| miR-301a | 1460703_at | Ascc1 | 0.561 | 0.007 |
| miR-301a | 1445481_at | AI317158 | -0.561 | 0.007 |
| miR-301a | 1417341_a_at | Ppp1r2 | 0.561 | 0.007 |
| miR-301a | 1416243_a_at | LOC100038991 /// LOC100040823 /// LOC100044559 /// LOC100048308 /// Rpl35 | 0.560 | 0.007 |
| miR-301a | 1417260_at | U2af2 | -0.560 | 0.007 |
| miR-301a | 1418375_at | Mbd6 | -0.558 | 0.007 |
| miR-301a | 1424197_s_at | Fance | 0.558 | 0.007 |
| miR-301a | 1416663_at | Ndufa9 | 0.557 | 0.007 |
| miR-301a | 1450632_at | LOC433749 /// Rhoa | 0.557 | 0.007 |
| miR-301a | 1428672_at | Snrpf | 0.557 | 0.007 |
| miR-301a | 1423215_at | Spcs2 | 0.557 | 0.007 |
| miR-301a | 1423160_at | Spred1 | 0.557 | 0.007 |
| miR-301a | 1442086_at | NA | -0.556 | 0.007 |
| miR-301a | 1438546_x_at | EG433923 /// EG623402 /// LOC630624 /// LOC667030 /// Slc25a5 | 0.556 | 0.007 |
| miR-301a | 1423822_a_at | Tmem168 | 0.556 | 0.007 |
| miR-301a | 1451076_s_at | Eif3m | 0.556 | 0.007 |
| miR-301a | 1451020_at | Gsk3b | -0.555 | 0.007 |
| miR-301a | 1440954_at | NA | -0.554 | 0.007 |
| miR-301a | 1418245_a_at | Rbm9 | 0.553 | 0.008 |
| miR-301a | 1417183_at | Dnaja2 | 0.553 | 0.008 |
| miR-301a | 1451234_at | BC021381 | -0.553 | 0.008 |
| miR-301a | 1460572_a_at | Zfp511 | -0.553 | 0.008 |
| miR-301a | 1420509_at | Srfbp1 | 0.553 | 0.008 |
| miR-301a | 1417964_at | Ap3d1 | -0.552 | 0.008 |
| miR-301a | 1455205_a_at | Usp19 | -0.552 | 0.008 |
| miR-301a | 1448335_s_at | Ccni | 0.551 | 0.008 |
| miR-301a | 1448192_s_at | AU021838 /// Mipol1 /// Prps1 | 0.550 | 0.008 |
| miR-301a | 1445718_at | NA | -0.550 | 0.008 |
| miR-301a | 1448487_at | Lrrfip1 | -0.550 | 0.008 |
| miR-301a | 1427882_at | Dnttip2 | 0.548 | 0.008 |
| miR-301a | 1419177_at | Vps37a | -0.548 | 0.008 |
| miR-301a | 1451587_a_at | Tiprl | 0.547 | 0.008 |
| miR-301a | 1417181_a_at | Kifap3 | -0.547 | 0.008 |
| miR-301a | 1423155_at | Sri | 0.547 | 0.008 |
| miR-301a | 1433539_at | Commd3 | 0.547 | 0.008 |
| miR-301a | 1417539_at | LOC100046775 /// Slc35a1 | 0.546 | 0.009 |
| miR-301a | 1451420_at | Ccdc47 | 0.545 | 0.009 |
| miR-301a | 1458491_at | 4930422I07Rik | 0.545 | 0.009 |
| miR-301a | 1416795_at | Cryl1 | 0.545 | 0.009 |
| miR-301a | 1450963_at | Hnrpf | 0.545 | 0.009 |
| miR-301a | 1427906_at | 1110037F02Rik | 0.542 | 0.009 |
| miR-301a | 1425845_a_at | Shoc2 | 0.542 | 0.009 |
| miR-301a | 1416244_a_at | Cnbp | 0.542 | 0.009 |
| miR-301a | 1419644_at | Cstf2 | 0.542 | 0.009 |
| miR-301a | 1421874_a_at | Mrps23 | 0.540 | 0.009 |
| miR-301a | 1428833_at | 4930406D14Rik | -0.540 | 0.010 |
| miR-301a | 1425053_at | Isoc1 | 0.539 | 0.010 |
| miR-301a | 1420368_at | Denr | 0.539 | 0.010 |
| miR-301a | 1425330_a_at | LOC100048520 /// LOC666025 /// Ppm1b | 0.539 | 0.010 |
| miR-301a | 1423080_at | LOC100043869 /// LOC100044630 /// Tomm20 | 0.539 | 0.010 |
| miR-301a | 1426398_at | Ube2w | 0.538 | 0.010 |
| miR-301a | 1436244_a_at | Tle2 | -0.538 | 0.010 |
| miR-301a | 1418633_at | Notch1 | -0.538 | 0.010 |
| miR-301a | 1444241_at | LOC100044766 | -0.537 | 0.010 |
| miR-301a | 1423479_at | Nol11 | 0.537 | 0.010 |
| miR-301a | 1425422_a_at | Parn | 0.536 | 0.010 |
| miR-301a | 1424442_a_at | Pja2 | -0.535 | 0.010 |
| miR-301a | 1454678_s_at | A130022J15Rik | -0.535 | 0.010 |
| miR-301a | 1433711_s_at | LOC100047324 /// Sesn1 | -0.534 | 0.010 |
| miR-301a | 1426467_s_at | 0610037L13Rik | 0.534 | 0.010 |
| miR-301a | 1425859_a_at | Psmd4 | 0.534 | 0.011 |
| miR-301a | 1416605_at | Nola2 | 0.533 | 0.011 |
| miR-301a | 1438913_x_at | Hdgfrp2 | -0.533 | 0.011 |
| miR-301a | 1421985_a_at | Eif4e2 | 0.533 | 0.011 |
| miR-301a | 1426370_at | Mlstd2 | 0.532 | 0.011 |
| miR-301a | 1457312_at | NA | -0.532 | 0.011 |
| miR-301a | 1448244_at | Lypla1 | 0.532 | 0.011 |
| miR-301a | 1439293_at | BC031353 | -0.532 | 0.011 |
| miR-301a | 1425979_a_at | Fbf1 | -0.531 | 0.011 |
| miR-301a | 1431320_a_at | Myo5a | -0.531 | 0.011 |
| miR-301a | 1435329_at | Fbxl11 | -0.531 | 0.011 |
| miR-301a | 1459363_at | Atxn2 | -0.531 | 0.011 |
| miR-301a | 1434801_x_at | Slc25a5 | 0.531 | 0.011 |
| miR-301a | 1425190_a_at | Phospho2 | 0.531 | 0.011 |
| miR-301a | 1415957_a_at | Rrp1 | 0.530 | 0.011 |
| miR-301a | 1425030_at | Zfp622 | 0.530 | 0.011 |
| miR-301a | 1454909_at | Tacc1 | -0.530 | 0.011 |
| miR-301a | 1421754_at | AY036118 | -0.530 | 0.011 |
| miR-301a | 1438070_at | Phf3 | 0.530 | 0.011 |
| miR-301a | 1453849_s_at | Hnrpab | 0.529 | 0.011 |
| miR-301a | 1426533_at | Nol5a | 0.529 | 0.011 |
| miR-301a | 1441649_at | NA | -0.529 | 0.011 |
| miR-301a | 1440222_at | Sod1 | -0.529 | 0.011 |
| miR-301a | 1417052_at | Psmb3 | 0.529 | 0.011 |
| miR-301a | 1455018_at | Lmtk2 | -0.529 | 0.011 |
| miR-301a | 1427314_at | Tmed7 | 0.529 | 0.011 |
| miR-301a | 1448206_at | Psma2 | 0.528 | 0.011 |
| miR-301a | 1434412_x_at | Stub1 | -0.528 | 0.012 |
| miR-301a | 1419754_at | Myo5a | -0.528 | 0.012 |
| miR-301a | 1459879_at | 4921513D23Rik | -0.528 | 0.012 |
| miR-301a | 1455548_at | Dlgap4 | -0.527 | 0.012 |
| miR-301a | 1416819_at | Cdc37 | 0.527 | 0.012 |
| miR-301a | 1460331_at | Tm9sf2 | 0.527 | 0.012 |
| miR-301a | 1454808_at | Efha1 | 0.526 | 0.012 |
| miR-301a | 1459010_at | NA | -0.526 | 0.012 |
| miR-301a | 1426386_at | Rpl7l1 | 0.526 | 0.012 |
| miR-301a | 1448505_at | C1d | 0.526 | 0.012 |
| miR-301a | 1436874_x_at | Slc25a5 | 0.525 | 0.012 |
| miR-301a | 1423728_at | Eif3eip | 0.524 | 0.012 |
| miR-301a | 1448828_at | Smc6 | 0.524 | 0.012 |
| miR-301a | 1430019_a_at | EG434858 /// Hnrnpa1 /// LOC654467 | 0.524 | 0.012 |
| miR-301a | 1444119_at | B930006L02Rik | -0.524 | 0.012 |
| miR-301a | 1450026_a_at | B3gnt2 | 0.524 | 0.012 |
| miR-301a | 1423997_at | Csde1 | 0.523 | 0.012 |
| miR-301a | 1448196_at | Mat2b | 0.523 | 0.012 |
| miR-301a | 1423385_at | Actr8 | 0.523 | 0.012 |
| miR-301a | 1421135_a_at | Cnot8 | 0.523 | 0.012 |
| miR-301a | 1417819_at | Tor1b | 0.523 | 0.013 |
| miR-301a | 1436342_a_at | D19Ertd721e | 0.523 | 0.013 |
| miR-301a | 1433684_at | Chmp6 | 0.522 | 0.013 |
| miR-301a | 1436033_at | BC031353 | -0.522 | 0.013 |
| miR-301a | 1456587_x_at | 2010005J08Rik | -0.522 | 0.013 |
| miR-301a | 1452175_at | 1810026J23Rik | 0.522 | 0.013 |
| miR-301a | 1428145_at | Acaa2 | 0.520 | 0.013 |
| miR-301a | 1458322_x_at | NA | -0.520 | 0.013 |
| miR-301a | 1426360_at | Zc3h11a | -0.520 | 0.013 |
| miR-301a | 1450424_a_at | Il18bp | 0.520 | 0.013 |
| miR-301a | 1426233_at | Map2k4 | 0.519 | 0.013 |
| miR-301a | 1433558_at | Dab2ip | -0.519 | 0.013 |
| miR-301a | 1454776_at | Ehmt1 | -0.518 | 0.013 |
| miR-301a | 1423095_s_at | Crbn | 0.518 | 0.014 |
| miR-301a | AFFX-GapdhMur/M32599_5_at | Gapdh | 0.517 | 0.014 |
| miR-301a | 1435445_at | Ccnt2 | -0.516 | 0.014 |
| miR-301a | 1454142_a_at | Pwp1 | 0.516 | 0.014 |
| miR-301a | 1440092_at | NA | -0.516 | 0.014 |
| miR-301a | 1451226_at | Pex6 | -0.516 | 0.014 |
| miR-301a | 1420868_s_at | LOC100042343 /// Tmed2 | 0.516 | 0.014 |
| miR-301a | 1443521_at | NA | -0.516 | 0.014 |
| miR-301a | 1430542_a_at | EG433923 /// LOC667030 /// Slc25a5 | 0.515 | 0.014 |
| miR-301a | 1450015_x_at | Sgpp1 | 0.515 | 0.014 |
| miR-301a | 1455884_at | Dpp9 | -0.515 | 0.014 |
| miR-301a | 1451302_at | 1110012L19Rik | 0.515 | 0.014 |
| miR-301a | 1444538_at | NA | -0.514 | 0.014 |
| miR-301a | 1439841_at | Zfyve27 | -0.514 | 0.014 |
| miR-301a | 1418250_at | Arl4d /// LOC100044157 | 0.513 | 0.015 |
| miR-301a | 1441574_at | NA | -0.513 | 0.015 |
| miR-301a | 1458708_at | NA | -0.513 | 0.015 |
| miR-301a | 1451504_at | Chchd3 | 0.513 | 0.015 |
| miR-301a | 1437741_at | Rab21 | 0.512 | 0.015 |
| miR-301a | 1451223_a_at | Btf3l4 | 0.512 | 0.015 |
| miR-301a | 1448256_at | Gosr1 | 0.512 | 0.015 |
| miR-301a | 1425273_s_at | Emp2 | 0.511 | 0.015 |
| miR-301a | 1437675_at | C130026L21Rik /// Slc8a1 | -0.511 | 0.015 |
| miR-301a | 1448699_at | Mrpl54 | 0.511 | 0.015 |
| miR-301a | 1424389_at | Nupl1 | 0.510 | 0.015 |
| miR-301a | 1427379_at | Pnpla6 | -0.510 | 0.015 |
| miR-301a | 1428542_at | Eif1ad | 0.510 | 0.015 |
| miR-301a | 1424520_at | 2010305A19Rik | 0.510 | 0.015 |
| miR-301a | 1421550_a_at | Trim34 | 0.510 | 0.015 |
| miR-301a | 1420668_a_at | Yipf2 | -0.509 | 0.015 |
| miR-301a | 1439074_a_at | Son | 0.509 | 0.016 |
| miR-301a | 1418911_s_at | Acsl4 | 0.508 | 0.016 |
| miR-301a | 1427349_x_at | 2810021G02Rik | 0.508 | 0.016 |
| miR-301a | AFFX-GapdhMur/M32599_M_at | Gapdh | 0.508 | 0.016 |
| miR-301a | 1455285_at | Slc31a1 | 0.507 | 0.016 |
| miR-301a | 1440061_at | Rbx1 | -0.507 | 0.016 |
| miR-301a | 1449626_s_at | Acbd4 | -0.507 | 0.016 |
| miR-301a | 1437729_at | EG665189 | -0.506 | 0.016 |
| miR-301a | 1422792_at | Pafah1b2 | 0.506 | 0.016 |
| miR-301a | 1460191_at | Ykt6 | 0.506 | 0.016 |
| miR-301a | 1438204_at | Hist1h1c | -0.506 | 0.016 |
| miR-301a | 1429186_a_at | Cdadc1 | 0.506 | 0.016 |
| miR-301a | 1423800_at | Dars | 0.505 | 0.016 |
| miR-301a | 1417665_a_at | Cpsf1 | -0.505 | 0.017 |
| miR-301a | 1450412_at | Tbl2 | 0.505 | 0.017 |
| miR-301a | 1437958_at | Xpr1 | -0.505 | 0.017 |
| miR-301a | 1440253_at | Psmd11 | 0.505 | 0.017 |
| miR-301a | 1428186_at | Kctd6 | -0.505 | 0.017 |
| miR-301a | 1430778_a_at | Nubp1 | 0.504 | 0.017 |
| miR-301a | 1420889_at | Hccs | 0.504 | 0.017 |
| miR-301a | 1458409_at | C86595 | -0.504 | 0.017 |
| miR-301a | 1451184_at | Hnrpa3 /// LOC100045099 /// LOC545592 | 0.503 | 0.017 |
| miR-301a | 1455552_at | Snapc4 | -0.503 | 0.017 |
| miR-301a | 1431345_a_at | Taf1b | 0.503 | 0.017 |
| miR-301a | 1423545_a_at | Zfp207 | 0.503 | 0.017 |
| miR-301a | 1432403_at | 4933402C06Rik | 0.503 | 0.017 |
| miR-301a | 1424355_a_at | Sin3b | 0.503 | 0.017 |
| miR-301a | 1443361_at | NA | -0.502 | 0.017 |
| miR-301a | 1451205_at | Psmb4 | 0.502 | 0.017 |
| miR-301a | 1429588_at | 2810474O19Rik | -0.502 | 0.017 |
| miR-301a | 1458710_at | NA | -0.502 | 0.017 |
| miR-301a | 1429096_at | 2810455D13Rik | -0.502 | 0.017 |
| miR-301a | 1458056_at | Sfrs12 | -0.502 | 0.017 |
| miR-301a | 1451292_at | Zfp212 | -0.501 | 0.017 |
| miR-301a | 1427468_at | Ppp3cb | 0.501 | 0.017 |
| miR-301a | 1450908_at | Eif4e | 0.501 | 0.017 |
| miR-301a | 1431028_a_at | Pank1 | -0.501 | 0.018 |
| miR-301a | 1438496_a_at | Ddx26b | -0.501 | 0.018 |
| miR-301a | 1423403_at | Mapkbp1 | -0.501 | 0.018 |
| miR-301a | 1451005_at | Sumo1 | 0.500 | 0.018 |
| miR-301a | 1459746_at | NA | -0.500 | 0.018 |
| miR-301a | 1423907_a_at | Ndufs8 | 0.500 | 0.018 |
| miR-301a | 1436372_a_at | Pdxdc1 | 0.500 | 0.018 |
| miR-301a | 1456623_at | Tpm1 | -0.500 | 0.018 |
| miR-301a | 1417350_at | Pldn | 0.500 | 0.018 |
| miR-301a | 1416973_at | EG434401 /// Nhp2l1 | 0.499 | 0.018 |
| miR-301a | 1448464_at | Ykt6 | 0.499 | 0.018 |
| miR-301a | 1424338_at | Slc6a13 | 0.499 | 0.018 |
| miR-301a | 1418258_s_at | Dynll2 | -0.499 | 0.018 |
| miR-301a | 1457711_at | Ranbp3 | -0.499 | 0.018 |
| miR-301a | 1444034_at | NA | -0.499 | 0.018 |
| miR-301a | 1420828_s_at | Ywhaq | 0.498 | 0.018 |
| miR-301a | 1424239_at | 2310066E14Rik | -0.498 | 0.018 |
| miR-301a | 1426503_a_at | Rnf121 | 0.498 | 0.018 |
| miR-301a | 1418144_a_at | Pip5k1a | -0.497 | 0.019 |
| miR-301a | 1440263_at | Nrp | -0.497 | 0.019 |
| miR-301a | 1460726_at | Adss | 0.497 | 0.019 |
| miR-301a | 1450534_x_at | H2-K1 | 0.497 | 0.019 |
| miR-301a | 1417118_a_at | Ard1 | 0.497 | 0.019 |
| miR-301a | 1460409_at | Cpt1a | 0.497 | 0.019 |
| miR-301a | 1427904_s_at | 2410091C18Rik | 0.496 | 0.019 |
| miR-301a | 1425085_at | 6330416L07Rik | 0.496 | 0.019 |
| miR-301a | 1453189_at | Ube2i | -0.496 | 0.019 |
| miR-301a | 1434288_at | Bivm | 0.496 | 0.019 |
| miR-301a | 1433609_s_at | Surf2 | 0.496 | 0.019 |
| miR-301a | 1428745_a_at | 2310003L22Rik | 0.496 | 0.019 |
| miR-301a | 1452801_at | Pigk | 0.496 | 0.019 |
| miR-301a | 1449928_at | Dynlt3 | 0.495 | 0.019 |
| miR-301a | 1419030_at | Ero1l | 0.495 | 0.019 |
| miR-301a | 1437628_s_at | Rhoa | 0.495 | 0.019 |
| miR-301a | 1442416_at | D930001B02 | -0.495 | 0.019 |
| miR-301a | 1424253_at | 1810073G14Rik | -0.495 | 0.019 |
| miR-301a | 1447314_at | NA | -0.495 | 0.019 |
| miR-301a | 1433682_at | Arhgef17 | 0.495 | 0.019 |
| miR-301a | 1460737_at | Igbp1 | 0.494 | 0.019 |
| miR-301a | 1425072_at | Skp2 | -0.494 | 0.019 |
| miR-301a | 1424517_at | Ccdc12 | 0.494 | 0.019 |
| miR-301a | 1435646_at | Ikbkg | 0.494 | 0.019 |
| miR-301a | 1449888_at | Epas1 /// LOC100048537 | 0.494 | 0.020 |
| miR-301a | 1443880_at | Zbtb39 | -0.494 | 0.020 |
| miR-301a | 1423772_x_at | Slc25a5 | 0.493 | 0.020 |
| miR-301a | 1439477_at | Ube2b | -0.493 | 0.020 |
| miR-301a | 1416685_s_at | Fbl /// LOC100044829 | 0.493 | 0.020 |
| miR-301a | 1452328_s_at | Pja2 | 0.493 | 0.020 |
| miR-301a | 1453564_a_at | Vps24 | 0.493 | 0.020 |
| miR-301a | 1442581_at | Ksr1 | -0.493 | 0.020 |
| miR-301a | 1457282_x_at | Tubgcp5 | -0.493 | 0.020 |
| miR-301a | 1429187_at | NA | 0.493 | 0.020 |
| miR-301a | 1423970_at | Thoc3 | 0.493 | 0.020 |
| miR-301a | 1417918_at | Mrpl11 | 0.492 | 0.020 |
| miR-301a | 1451056_at | Psmd7 | 0.492 | 0.020 |
| miR-301a | 1450846_at | Bzw1 | 0.492 | 0.020 |
| miR-301a | 1452059_at | Slc35f5 | 0.492 | 0.020 |
| miR-301a | 1433860_at | 6030458C11Rik | -0.492 | 0.020 |
| miR-301a | 1416800_at | Trpm7 | 0.491 | 0.020 |
| miR-301a | 1419251_at | Eps15 | 0.491 | 0.020 |
| miR-301a | 1448336_at | Drg1 | 0.491 | 0.020 |
| miR-301a | 1448165_at | Casp2 | 0.490 | 0.021 |
| miR-301a | 1416521_at | Sepw1 | 0.490 | 0.021 |
| miR-301a | 1416020_a_at | Atp5g1 /// LOC100041835 | 0.490 | 0.021 |
| miR-301a | 1455113_at | Armc8 | -0.489 | 0.021 |
| miR-301a | 1438545_at | EG433923 /// EG623402 /// LOC630624 /// LOC667030 /// Slc25a5 | 0.489 | 0.021 |
| miR-301a | 1445862_at | 4921513D23Rik | -0.489 | 0.021 |
| miR-301a | 1429244_at | 1500011B03Rik /// 2610524H06Rik | 0.489 | 0.021 |
| miR-301a | 1425942_a_at | Gpm6b | 0.489 | 0.021 |
| miR-301a | 1421971_a_at | Mrps34 | 0.489 | 0.021 |
| miR-301a | 1434178_at | Mll3 | -0.489 | 0.021 |
| miR-301a | 1448736_a_at | Hprt1 | 0.489 | 0.021 |
| miR-301a | 1448634_at | Ralbp1 | -0.488 | 0.021 |
| miR-301a | 1419372_at | Gosr2 | 0.487 | 0.021 |
| miR-301a | 1418502_a_at | Oxr1 | 0.487 | 0.022 |
| miR-301a | 1450107_a_at | Renbp | 0.487 | 0.022 |
| miR-301a | 1444217_at | Mrpl38 | -0.487 | 0.022 |
| miR-301a | 1426414_a_at | Rnf7 | 0.487 | 0.022 |
| miR-301a | 1448951_at | Tnfrsf1b | 0.487 | 0.022 |
| miR-301a | 1444578_at | NA | -0.486 | 0.022 |
| miR-301a | 1436760_a_at | EG629732 /// LOC100039036 /// LOC100040298 /// LOC100047156 /// LOC100048094 /// OTTMUSG00000000623 /// Rps8 | 0.486 | 0.022 |
| miR-301a | 1418023_at | Narg1 | 0.486 | 0.022 |
| miR-301a | 1416110_at | Slc35a4 | 0.486 | 0.022 |
| miR-301a | 1450208_a_at | Elmo1 | 0.486 | 0.022 |
| miR-301a | 1442535_at | NA | -0.486 | 0.022 |
| miR-301a | 1428520_at | 1110032A13Rik | -0.485 | 0.022 |
| miR-301a | 1428153_at | Mrps10 | 0.485 | 0.022 |
| miR-301a | 1432045_at | Tssk5 | -0.485 | 0.022 |
| miR-301a | 1423047_at | Tollip | -0.485 | 0.022 |
| miR-301a | 1455282_x_at | Alas1 | -0.485 | 0.022 |
| miR-301a | 1417659_at | Vps29 | 0.485 | 0.022 |
| miR-301a | 1448928_at | Hdac6 | -0.485 | 0.022 |
| miR-301a | 1449070_x_at | Apcdd1 | 0.485 | 0.022 |
| miR-301a | 1450711_at | Brd4 | -0.485 | 0.022 |
| miR-301a | 1457751_at | 4832420A03Rik /// Rsf1 | 0.485 | 0.022 |
| miR-301a | 1438771_at | Brd1 /// LOC100045983 | -0.485 | 0.022 |
| miR-301a | 1423082_at | Derl1 | 0.485 | 0.022 |
| miR-301a | 1440952_at | NA | -0.484 | 0.022 |
| miR-301a | 1427351_s_at | Igh-6 | -0.484 | 0.022 |
| miR-301a | 1422547_at | Ranbp1 | 0.484 | 0.022 |
| miR-301a | 1425561_at | Trnt1 | 0.483 | 0.023 |
| miR-301a | 1436706_at | Tmem32 | 0.483 | 0.023 |
| miR-301a | 1424741_s_at | Creb3 | 0.483 | 0.023 |
| miR-301a | 1427030_at | Ccdc52 | -0.483 | 0.023 |
| miR-301a | 1417779_at | 2310079N02Rik | 0.483 | 0.023 |
| miR-301a | 1448579_at | Glg1 | -0.483 | 0.023 |
| miR-301a | 1416974_at | Stam2 | 0.483 | 0.023 |
| miR-301a | 1446644_at | NA | -0.483 | 0.023 |
| miR-301a | 1419189_at | Vti1a | 0.483 | 0.023 |
| miR-301a | 1418285_at | Efnb1 | -0.483 | 0.023 |
| miR-301a | 1421335_a_at | Egfl7 | -0.483 | 0.023 |
| miR-301a | 1433980_at | Htatip | 0.482 | 0.023 |
| miR-301a | 1456054_a_at | Pum1 | -0.482 | 0.023 |
| miR-301a | 1423423_at | Pdia3 | 0.482 | 0.023 |
| miR-301a | 1457797_at | AI605517 | -0.482 | 0.023 |
| miR-301a | 1426275_a_at | Uxs1 | 0.482 | 0.023 |
| miR-301a | 1451540_at | Mpi | 0.482 | 0.023 |
| miR-301a | 1417762_a_at | Rpl8 | 0.482 | 0.023 |
| miR-301a | 1454695_at | Wdr18 | -0.482 | 0.023 |
| miR-301a | 1423322_at | Lin7c | 0.482 | 0.023 |
| miR-301a | 1429735_at | 1110003F05Rik | -0.482 | 0.023 |
| miR-301a | 1417453_at | Cul4b | 0.481 | 0.023 |
| miR-301a | 1444609_at | NA | -0.481 | 0.023 |
| miR-301a | 1425331_at | Zfp106 | 0.481 | 0.023 |
| miR-301a | 1424653_at | Tspan15 | 0.481 | 0.023 |
| miR-301a | 1458280_at | NA | -0.481 | 0.023 |
| miR-301a | 1444354_at | NA | -0.481 | 0.023 |
| miR-301a | 1423058_at | Capza2 | 0.481 | 0.024 |
| miR-301a | 1416109_at | Fuca1 | 0.480 | 0.024 |
| miR-301a | 1435559_at | Myo6 | -0.480 | 0.024 |
| miR-301a | 1430343_at | Nup205 | 0.480 | 0.024 |
| miR-301a | 1428515_at | 2410012H22Rik | 0.480 | 0.024 |
| miR-301a | 1450396_at | Stag2 | 0.480 | 0.024 |
| miR-301a | 1417087_at | Glg1 | -0.480 | 0.024 |
| miR-301a | 1417034_at | Trappc6a | 0.479 | 0.024 |
| miR-301a | 1445148_at | NA | -0.479 | 0.024 |
| miR-301a | 1444473_at | NA | -0.479 | 0.024 |
| miR-301a | 1428700_at | P2ry13 | 0.479 | 0.024 |
| miR-301a | 1447491_at | NA | -0.479 | 0.024 |
| miR-301a | 1448309_at | Ap3m1 | 0.479 | 0.024 |
| miR-301a | 1426094_at | Rhbdl1 | -0.479 | 0.024 |
| miR-301a | 1422793_at | Pafah1b2 | 0.479 | 0.024 |
| miR-301a | 1437199_at | NA | -0.478 | 0.024 |
| miR-301a | 1457073_at | NA | -0.478 | 0.024 |
| miR-301a | 1424258_at | Polr2d | 0.478 | 0.024 |
| miR-301a | 1416684_at | Fbl /// LOC100044829 | 0.478 | 0.024 |
| miR-301a | 1428146_s_at | Acaa2 | 0.478 | 0.024 |
| miR-301a | 1426374_at | 2410166I05Rik | 0.478 | 0.024 |
| miR-301a | 1416909_at | Pigyl | 0.478 | 0.025 |
| miR-301a | 1430020_x_at | Hnrnpa1 | 0.478 | 0.025 |
| miR-301a | 1435823_x_at | Egfl7 | -0.478 | 0.025 |
| miR-301a | 1429476_s_at | Dnaja2 | 0.478 | 0.025 |
| miR-301a | 1422491_a_at | Bnip2 | 0.478 | 0.025 |
| miR-301a | 1424122_s_at | Commd1 | 0.478 | 0.025 |
| miR-301a | 1450130_at | Xpr1 | 0.477 | 0.025 |
| miR-301a | 1436181_at | Ddef2 | -0.477 | 0.025 |
| miR-301a | 1426692_at | Ccdc97 | -0.477 | 0.025 |
| miR-301a | 1417868_a_at | Ctsz | 0.477 | 0.025 |
| miR-301a | 1416770_at | Stk25 | 0.477 | 0.025 |
| miR-301a | 1427334_s_at | 2810474O19Rik | -0.477 | 0.025 |
| miR-301a | 1448141_at | 1110014J01Rik | 0.477 | 0.025 |
| miR-301a | 1416732_at | Top2b | 0.477 | 0.025 |
| miR-301a | 1440824_at | NA | -0.476 | 0.025 |
| miR-301a | 1453156_s_at | Zadh1 | 0.476 | 0.025 |
| miR-301a | 1420616_at | Ash2l | 0.476 | 0.025 |
| miR-301a | 1429487_at | Ppp1r12a | -0.476 | 0.025 |
| miR-301a | 1433433_at | Myst2 | 0.476 | 0.025 |
| miR-301a | 1435427_x_at | BC037112 | -0.476 | 0.025 |
| miR-301a | 1452190_at | Prcp | 0.476 | 0.025 |
| miR-301a | 1441969_at | Trim36 | -0.476 | 0.025 |
| miR-301a | 1426975_at | Os9 | 0.476 | 0.025 |
| miR-301a | 1424500_at | Utp6 | 0.476 | 0.025 |
| miR-301a | 1424620_at | D13Wsu177e | 0.476 | 0.025 |
| miR-301a | 1428957_at | Tmem177 | 0.476 | 0.025 |
| miR-301a | 1448501_at | Tspan6 | 0.475 | 0.025 |
| miR-301a | 1424029_at | Tspyl4 | -0.475 | 0.025 |
| miR-301a | 1423248_at | Nktr | -0.475 | 0.025 |
| miR-301a | 1430053_a_at | Ola1 | 0.475 | 0.025 |
| miR-301a | 1450016_at | Ccng1 | 0.475 | 0.026 |
| miR-301a | 1444430_at | NA | -0.475 | 0.026 |
| miR-301a | 1438971_x_at | Ube2h | -0.475 | 0.026 |
| miR-301a | 1445512_at | NA | -0.475 | 0.026 |
| miR-301a | 1452461_a_at | Gnptab | 0.474 | 0.026 |
| miR-301a | 1434776_at | Sema5a | 0.474 | 0.026 |
| miR-301a | 1436202_at | Malat1 | -0.474 | 0.026 |
| miR-301a | 1455069_x_at | Slc25a4 | 0.474 | 0.026 |
| miR-301a | 1419168_at | Mapk6 | 0.474 | 0.026 |
| miR-301a | 1418912_at | Plxdc2 | 0.474 | 0.026 |
| miR-301a | 1454174_a_at | C330007P06Rik | 0.474 | 0.026 |
| miR-301a | 1418433_at | Cab39 | 0.474 | 0.026 |
| miR-301a | 1416997_a_at | Hap1 | -0.474 | 0.026 |
| miR-301a | 1416791_a_at | Nxf1 | -0.473 | 0.026 |
| miR-301a | 1429066_at | 4930565B19Rik | -0.473 | 0.026 |
| miR-301a | 1424129_at | Mfsd1 | 0.473 | 0.026 |
| miR-301a | 1450017_at | Ccng1 | 0.473 | 0.026 |
| miR-301a | 1436131_at | Siglech | 0.473 | 0.026 |
| miR-301a | 1425576_at | Ahcyl1 | 0.473 | 0.026 |
| miR-301a | 1430980_a_at | Eif4a1 | 0.472 | 0.026 |
| miR-301a | 1452673_at | Ranbp3 | -0.472 | 0.026 |
| miR-301a | 1432384_a_at | Mettl6 | 0.472 | 0.027 |
| miR-301a | 1424928_at | 2210018M11Rik | -0.472 | 0.027 |
| miR-301a | 1417772_at | Grhpr | 0.471 | 0.027 |
| miR-301a | 1435946_at | Sepsecs | 0.471 | 0.027 |
| miR-301a | 1441522_at | Dcun1d2 | -0.471 | 0.027 |
| miR-301a | 1454243_at | Ick | -0.471 | 0.027 |
| miR-301a | 1423133_at | Cwc15 | 0.471 | 0.027 |
| miR-301a | 1445003_at | Farsa | -0.471 | 0.027 |
| miR-301a | 1423908_at | Ndufs8 | 0.471 | 0.027 |
| miR-301a | 1448589_at | Ndufb5 | 0.471 | 0.027 |
| miR-301a | 1431997_at | 3000002C10Rik | 0.471 | 0.027 |
| miR-301a | 1460295_s_at | Il6st | 0.471 | 0.027 |
| miR-301a | 1423041_a_at | Bzw1 | 0.471 | 0.027 |
| miR-301a | 1450937_at | Lin7c | 0.470 | 0.027 |
| miR-301a | 1451283_at | 1810073G14Rik | 0.470 | 0.027 |
| miR-301a | 1415674_a_at | Trappc4 | 0.470 | 0.027 |
| miR-301a | 1437022_at | D130059P03Rik | -0.470 | 0.027 |
| miR-301a | 1451458_at | Tmem2 | 0.470 | 0.027 |
| miR-301a | 1457707_at | Mctp2 | 0.470 | 0.027 |
| miR-301a | 1448642_at | Pcbp1 | 0.470 | 0.027 |
| miR-301a | 1453726_s_at | 2810407C02Rik | 0.470 | 0.027 |
| miR-301a | 1424924_at | Sec63 | 0.470 | 0.027 |
| miR-301a | 1429199_s_at | Saal1 | 0.469 | 0.028 |
| miR-301a | 1416917_at | 1700123O20Rik | 0.469 | 0.028 |
| miR-301a | 1438419_at | Rbm16 | -0.469 | 0.028 |
| miR-301a | 1460704_at | Rfng | -0.469 | 0.028 |
| miR-301a | 1426723_at | Wdr48 | -0.469 | 0.028 |
| miR-301a | 1437056_x_at | Crispld2 | -0.469 | 0.028 |
| miR-301a | 1418067_at | Cfl2 | 0.469 | 0.028 |
| miR-301a | 1439800_at | Clip1 | -0.469 | 0.028 |
| miR-301a | 1451090_a_at | Eif2s3x | 0.469 | 0.028 |
| miR-301a | 1418292_at | Asna1 | 0.468 | 0.028 |
| miR-301a | 1423202_a_at | Ncor1 | -0.468 | 0.028 |
| miR-301a | 1441013_at | C81521 | -0.468 | 0.028 |
| miR-301a | 1431431_a_at | LOC100045796 /// Nfs1 | 0.468 | 0.028 |
| miR-301a | 1434054_at | Mafg | -0.468 | 0.028 |
| miR-301a | 1416019_at | Dr1 | 0.467 | 0.028 |
| miR-301a | 1455207_at | 2410017P09Rik | 0.467 | 0.028 |
| miR-301a | 1450385_at | Kpna3 | 0.467 | 0.029 |
| miR-301a | 1441594_at | NA | -0.467 | 0.029 |
| miR-301a | 1435885_s_at | Itsn1 | -0.467 | 0.029 |
| miR-301a | 1455204_at | Pitpnc1 | -0.466 | 0.029 |
| miR-301a | 1452113_a_at | Rab23 | 0.466 | 0.029 |
| miR-301a | 1430780_a_at | Pmm1 | 0.466 | 0.029 |
| miR-301a | 1439214_a_at | Api5 | 0.466 | 0.029 |
| miR-301a | 1423053_at | Arf4 | 0.466 | 0.029 |
| miR-301a | 1415991_a_at | Klhdc3 | -0.466 | 0.029 |
| miR-301a | 1451397_at | Gigyf2 | -0.466 | 0.029 |
| miR-301a | 1427785_x_at | Solh | -0.466 | 0.029 |
| miR-301a | 1425665_a_at | Srp54a /// Srp54b /// Srp54c | 0.466 | 0.029 |
| miR-301a | 1424050_s_at | Fgfr1 | -0.466 | 0.029 |
| miR-301a | 1416060_at | Tbc1d15 | 0.466 | 0.029 |
| miR-301a | 1426585_s_at | Mapk1 | 0.465 | 0.029 |
| miR-301a | 1424006_at | Aarsd1 | 0.465 | 0.029 |
| miR-301a | 1430622_at | 4833423F13Rik | -0.465 | 0.029 |
| miR-301a | 1456216_at | NA | -0.465 | 0.029 |
| miR-301a | 1418064_at | Tfpt | 0.465 | 0.029 |
| miR-301a | 1435032_at | Golgb1 | -0.465 | 0.029 |
| miR-301a | 1451393_at | Pex26 | -0.465 | 0.029 |
| miR-301a | 1420652_at | Ate1 | 0.465 | 0.029 |
| miR-301a | 1459240_at | Tcf25 | -0.465 | 0.029 |
| miR-301a | 1450907_at | Spcs2 | 0.465 | 0.029 |
| miR-301a | 1450180_a_at | Rara | 0.465 | 0.029 |
| miR-301a | 1441656_at | B930068K11Rik | -0.465 | 0.029 |
| miR-301a | 1428508_at | Tbc1d2b | -0.465 | 0.029 |
| miR-301a | 1421872_at | Rab24 | 0.464 | 0.029 |
| miR-301a | 1435782_at | LOC668206 | 0.464 | 0.029 |
| miR-301a | 1429533_at | Immt | 0.464 | 0.029 |
| miR-301a | 1421871_at | Sh3bgrl | 0.464 | 0.030 |
| miR-301a | 1416386_a_at | M6pr | 0.464 | 0.030 |
| miR-301a | 1431113_at | Rbm4b | -0.464 | 0.030 |
| miR-301a | 1460575_at | Eif2a | 0.464 | 0.030 |
| miR-301a | 1431400_a_at | Gas7 | 0.463 | 0.030 |
| miR-301a | 1422863_s_at | Pdlim5 | 0.463 | 0.030 |
| miR-301a | 1423181_s_at | Clns1a /// ENSMUSG00000056003 /// LOC100040211 | 0.463 | 0.030 |
| miR-301a | 1422697_s_at | Jarid2 | -0.463 | 0.030 |
| miR-301a | 1458247_s_at | Dctn5 | -0.463 | 0.030 |
| miR-301a | 1416212_at | Magoh | 0.463 | 0.030 |
| miR-301a | 1450845_a_at | Bzw1 | 0.463 | 0.030 |
| miR-301a | 1448266_at | Edf1 | 0.463 | 0.030 |
| miR-301a | 1460402_at | Brpf1 | -0.463 | 0.030 |
| miR-301a | 1459890_s_at | 1110008P14Rik | 0.463 | 0.030 |
| miR-301a | 1426948_at | Tpr | 0.462 | 0.030 |
| miR-301a | 1438535_at | Phip | -0.462 | 0.030 |
| miR-301a | 1416651_at | Znhit2 | 0.462 | 0.030 |
| miR-301a | 1458616_at | NA | -0.462 | 0.030 |
| miR-301a | 1417177_at | Galk1 | 0.462 | 0.030 |
| miR-301a | 1424080_at | Dcps | 0.462 | 0.030 |
| miR-301a | 1450038_s_at | Usp9x | 0.462 | 0.030 |
| miR-301a | 1423088_at | Tmod3 | 0.462 | 0.030 |
| miR-301a | 1451121_a_at | Gltscr2 | 0.462 | 0.030 |
| miR-301a | 1422959_s_at | Zfp313 | 0.462 | 0.031 |
| miR-301a | 1423254_x_at | Rps27l | 0.462 | 0.031 |
| miR-301a | 1422451_at | Mrps21 | 0.461 | 0.031 |
| miR-301a | 1434419_s_at | Tardbp | 0.461 | 0.031 |
| miR-301a | 1455480_s_at | Ube2d3 | 0.461 | 0.031 |
| miR-301a | 1421876_at | Mapk9 | 0.461 | 0.031 |
| miR-301a | 1419367_at | Decr1 | 0.461 | 0.031 |
| miR-301a | 1426918_at | Itgb1 | 0.461 | 0.031 |
| miR-301a | 1451314_a_at | Vcam1 | 0.460 | 0.031 |
| miR-301a | 1423744_x_at | Eif2s3x | 0.460 | 0.031 |
| miR-301a | 1449262_s_at | Lin7c | 0.460 | 0.031 |
| miR-301a | 1453949_s_at | Lypla1 | 0.460 | 0.031 |
| miR-301a | 1452712_at | Hnrpa3 | 0.460 | 0.031 |
| miR-301a | 1420113_s_at | 2410022L05Rik | -0.460 | 0.031 |
| miR-301a | 1425676_a_at | Elovl1 | 0.459 | 0.032 |
| miR-301a | 1452000_s_at | Sars | 0.459 | 0.032 |
| miR-301a | 1417727_at | Sfrs9 | 0.459 | 0.032 |
| miR-301a | 1434213_x_at | Ndufs8 | 0.459 | 0.032 |
| miR-301a | 1433938_at | Trp53bp2 | 0.458 | 0.032 |
| miR-301a | 1439016_x_at | Sprr2a | 0.458 | 0.032 |
| miR-301a | 1456880_at | NA | -0.458 | 0.032 |
| miR-301a | 1424837_at | Rnf113a1 | 0.458 | 0.032 |
| miR-301a | 1456253_s_at | Klhl17 /// Plekhn1 | -0.457 | 0.032 |
| miR-301a | 1460165_at | Ppp1ca | 0.457 | 0.032 |
| miR-301a | 1441688_at | 6430537I21Rik | -0.457 | 0.032 |
| miR-301a | 1422510_at | Ctdspl | -0.457 | 0.032 |
| miR-301a | 1444143_at | NA | -0.457 | 0.032 |
| miR-301a | 1442760_x_at | NA | -0.457 | 0.033 |
| miR-301a | 1425448_x_at | Atp6v0b | 0.457 | 0.033 |
| miR-301a | 1448563_at | Phb | 0.457 | 0.033 |
| miR-301a | 1447019_at | Cmah | -0.457 | 0.033 |
| miR-301a | 1417126_a_at | Rpl22l1 | 0.457 | 0.033 |
| miR-301a | 1448668_a_at | Irak1 | -0.456 | 0.033 |
| miR-301a | 1418114_at | Rbpj | 0.456 | 0.033 |
| miR-301a | 1446566_at | NA | -0.456 | 0.033 |
| miR-301a | 1435481_at | Zfp653 | -0.456 | 0.033 |
| miR-301a | 1417902_at | Slc19a2 | 0.456 | 0.033 |
| miR-301a | 1427245_at | Arfgap1 | -0.456 | 0.033 |
| miR-301a | 1426164_a_at | Usf1 | 0.456 | 0.033 |
| miR-301a | 1426266_s_at | Zbtb8os | 0.456 | 0.033 |
| miR-301a | 1453022_at | Gpihbp1 | -0.456 | 0.033 |
| miR-301a | 1424732_s_at | Tmem192 | 0.456 | 0.033 |
| miR-301a | 1423894_a_at | Dalrd3 | -0.456 | 0.033 |
| miR-301a | 1458309_at | NA | -0.456 | 0.033 |
| miR-301a | 1422432_at | Dbi | 0.456 | 0.033 |
| miR-301a | 1439465_x_at | Agbl5 | -0.456 | 0.033 |
| miR-301a | 1418625_s_at | 3000002C10Rik /// EG433184 /// EG433273 /// EG544878 /// EG545741 /// EG666342 /// EG666488 /// Gapdh /// LOC100039214 /// LOC100039229 /// LOC100039258 /// LOC100039556 /// LOC100039762 /// LOC100039840 /// LOC100040053 /// LOC100040109 /// LOC100040634 /// LOC100041204 /// LOC100041325 /// LOC100041748 /// LOC100042025 /// LOC100042349 /// LOC100042375 /// LOC100043724 /// LOC100044454 /// LOC100044707 /// LOC100044981 /// LOC100045908 /// LOC100046067 /// LOC100046224 /// LOC100047637 /// LOC100048253 /// LOC100048291 /// LOC100048329 /// LOC100048639 /// LOC433845 /// LOC435292 /// LOC629081 /// LOC665922 /// LOC666891 /// LOC668010 /// LOC668275 /// LOC674324 /// LOC676112 /// OTTMUSG00000005300 /// RP23-403O15.4 | 0.456 | 0.033 |
| miR-301a | 1423108_at | Slc25a20 | 0.456 | 0.033 |
| miR-301a | 1416439_at | 2410015N17Rik | 0.455 | 0.033 |
| miR-301a | 1427720_a_at | Rrp1 | 0.455 | 0.033 |
| miR-301a | 1455655_a_at | Tardbp | 0.455 | 0.033 |
| miR-301a | 1451519_at | Rnf2 | 0.455 | 0.034 |
| miR-301a | 1426400_a_at | Capns1 | 0.454 | 0.034 |
| miR-301a | 1422884_at | Snrpd3 | 0.454 | 0.034 |
| miR-301a | 1437576_at | 2810427A07Rik | -0.454 | 0.034 |
| miR-301a | 1421276_a_at | Dst | 0.454 | 0.034 |
| miR-301a | 1460545_at | Thrap3 | 0.454 | 0.034 |
| miR-301a | 1459622_at | Gm22 | -0.454 | 0.034 |
| miR-301a | 1420396_at | Cd160 | 0.454 | 0.034 |
| miR-301a | 1428212_x_at | EG665562 /// EG667682 /// LOC100042740 /// LOC100047426 /// LOC638399 /// LOC673582 /// LOC675018 /// Rpl31 | 0.454 | 0.034 |
| miR-301a | 1422799_at | Bat2 | -0.454 | 0.034 |
| miR-301a | 1423051_at | Hnrnpu | 0.454 | 0.034 |
| miR-301a | 1433604_x_at | Aldoa | 0.454 | 0.034 |
| miR-301a | 1420651_at | Ate1 | 0.453 | 0.034 |
| miR-301a | 1453175_at | Zbtb25 | 0.453 | 0.034 |
| miR-301a | 1417561_at | Apoc1 | 0.453 | 0.034 |
| miR-301a | 1460458_at | Crispld2 | -0.452 | 0.035 |
| miR-301a | 1421504_at | Sp4 | -0.452 | 0.035 |
| miR-301a | 1419950_s_at | Tnpo3 | -0.452 | 0.035 |
| miR-301a | 1423451_at | Pgrmc1 | 0.452 | 0.035 |
| miR-301a | 1429214_at | Adamtsl2 | -0.452 | 0.035 |
| miR-301a | 1456172_at | NA | -0.452 | 0.035 |
| miR-301a | 1439656_at | Pafah1b1 | -0.452 | 0.035 |
| miR-301a | 1427560_at | Six5 | -0.452 | 0.035 |
| miR-301a | 1442447_at | NA | -0.451 | 0.035 |
| miR-301a | 1438663_at | Bat2d | -0.451 | 0.035 |
| miR-301a | 1418192_at | Mnt | -0.451 | 0.035 |
| miR-301a | 1448373_at | Mrpl18 | 0.451 | 0.035 |
| miR-301a | 1416374_at | Ap3m1 | 0.451 | 0.035 |
| miR-301a | 1452030_a_at | Hnrnpr | 0.451 | 0.035 |
| miR-301a | 1421051_s_at | Vps25 | 0.450 | 0.035 |
| miR-301a | 1425051_at | Isoc1 | 0.450 | 0.035 |
| miR-301a | 1423114_at | Ube2d3 | 0.450 | 0.035 |
| miR-301a | 1426496_at | Wdr55 | 0.450 | 0.035 |
| miR-301a | 1448286_at | Hsd17b10 | 0.450 | 0.035 |
| miR-301a | 1448916_at | LOC100047868 /// Mafg | -0.450 | 0.035 |
| miR-301a | 1428194_at | Usp9x | 0.450 | 0.036 |
| miR-301a | 1452427_s_at | Ptplad1 | 0.450 | 0.036 |
| miR-301a | 1428869_at | Nolc1 | 0.450 | 0.036 |
| miR-301a | 1441370_at | Tmcc1 | -0.450 | 0.036 |
| miR-301a | 1456352_a_at | Sf3b2 | 0.450 | 0.036 |
| miR-301a | 1451726_at | Mtmr6 | 0.450 | 0.036 |
| miR-301a | 1426343_at | Stt3b | 0.450 | 0.036 |
| miR-301a | 1450522_a_at | H1f0 | 0.450 | 0.036 |
| miR-301a | 1418143_at | Vps45 | 0.450 | 0.036 |
| miR-301a | 1439192_at | Nova2 | -0.449 | 0.036 |
| miR-301a | 1450769_s_at | Stard5 | 0.449 | 0.036 |
| miR-301a | 1426832_at | Ddx26b | -0.449 | 0.036 |
| miR-301a | 1436316_at | 9430029L20Rik | -0.449 | 0.036 |
| miR-301a | 1454875_a_at | Rbbp4 | 0.449 | 0.036 |
| miR-301a | 1433209_at | 2210017G18Rik | -0.448 | 0.036 |
| miR-301a | 1422791_at | Pafah1b2 | 0.448 | 0.036 |
| miR-301a | 1447320_x_at | LOC100039220 /// Rpo1-3 | 0.448 | 0.036 |
| miR-301a | 1423927_at | Slc35b2 | 0.448 | 0.036 |
| miR-301a | 1448268_at | Tmed9 | 0.448 | 0.036 |
| miR-301a | 1417322_at | Dohh | 0.448 | 0.037 |
| miR-301a | 1457268_at | Dot1l | -0.448 | 0.037 |
| miR-301a | 1424275_s_at | LOC100046003 /// Trim41 | -0.448 | 0.037 |
| miR-301a | 1452075_at | Pus10 | 0.448 | 0.037 |
| miR-301a | 1434844_at | Hexdc | -0.448 | 0.037 |
| miR-301a | 1417398_at | Rras2 | 0.448 | 0.037 |
| miR-301a | 1428971_at | Ccny /// LOC100044842 | 0.447 | 0.037 |
| miR-301a | 1429834_a_at | 1110014N23Rik | 0.447 | 0.037 |
| miR-301a | 1452860_at | Fbxl17 | -0.447 | 0.037 |
| miR-301a | 1435874_at | Prkab2 | -0.447 | 0.037 |
| miR-301a | 1440274_at | NA | -0.447 | 0.037 |
| miR-301a | 1456808_at | NA | -0.447 | 0.037 |
| miR-301a | 1440034_at | Stam2 | -0.447 | 0.037 |
| miR-301a | 1456738_s_at | Brp16 | -0.447 | 0.037 |
| miR-301a | 1415852_at | Impdh2 | 0.447 | 0.037 |
| miR-301a | 1453367_a_at | Abhd12 | 0.446 | 0.037 |
| miR-301a | 1427978_at | 4732418C07Rik | -0.446 | 0.037 |
| miR-301a | 1437297_at | Chd8 | -0.446 | 0.037 |
| miR-301a | 1450083_at | Cnot4 | -0.446 | 0.037 |
| miR-301a | 1453448_at | 2310067E19Rik | -0.446 | 0.037 |
| miR-301a | 1438276_at | NA | -0.446 | 0.037 |
| miR-301a | 1423211_at | Nola3 | 0.446 | 0.037 |
| miR-301a | 1449062_at | Khk | 0.446 | 0.038 |
| miR-301a | 1447631_at | Myst2 | -0.446 | 0.038 |
| miR-301a | 1428000_at | Tmem60 | 0.446 | 0.038 |
| miR-301a | 1445914_at | Nrf1 | -0.446 | 0.038 |
| miR-301a | 1423409_a_at | 2500003M10Rik | 0.446 | 0.038 |
| miR-301a | 1426483_at | Prkrir | 0.446 | 0.038 |
| miR-301a | 1460049_s_at | 1500015O10Rik | -0.446 | 0.038 |
| miR-301a | 1435161_at | Sepsecs | -0.446 | 0.038 |
| miR-301a | 1455564_at | Bcr | -0.445 | 0.038 |
| miR-301a | 1425097_a_at | Zfp106 | 0.445 | 0.038 |
| miR-301a | 1425674_a_at | Ssu72 | 0.445 | 0.038 |
| miR-301a | 1447481_at | NA | -0.445 | 0.038 |
| miR-301a | 1460034_at | Samd4b | -0.445 | 0.038 |
| miR-301a | 1455676_x_at | Tial1 | -0.445 | 0.038 |
| miR-301a | 1434066_at | Gtf3c1 | -0.445 | 0.038 |
| miR-301a | 1415818_at | Anxa6 | 0.445 | 0.038 |
| miR-301a | 1438682_at | Pik3r1 | -0.445 | 0.038 |
| miR-301a | 1424681_a_at | Psma5 | 0.445 | 0.038 |
| miR-301a | 1434352_at | B630005N14Rik | 0.445 | 0.038 |
| miR-301a | 1457598_at | Glrx3 | -0.445 | 0.038 |
| miR-301a | 1437470_at | Pknox1 | -0.444 | 0.038 |
| miR-301a | 1444624_at | Ubap1 | -0.444 | 0.038 |
| miR-301a | 1438246_at | Csnk1g1 | -0.444 | 0.038 |
| miR-301a | 1427058_at | Eif4a1 | 0.444 | 0.038 |
| miR-301a | 1435675_at | Tbc1d12 | 0.444 | 0.038 |
| miR-301a | 1452341_at | Echs1 | 0.444 | 0.039 |
| miR-301a | 1446022_at | NA | -0.443 | 0.039 |
| miR-301a | 1433998_at | 4933427D14Rik | -0.443 | 0.039 |
| miR-301a | 1435755_at | 1110001A16Rik | 0.443 | 0.039 |
| miR-301a | 1453171_s_at | Ppm1a | -0.443 | 0.039 |
| miR-301a | 1450676_at | Tceb3 | 0.443 | 0.039 |
| miR-301a | 1416629_at | Slc1a5 | -0.443 | 0.039 |
| miR-301a | 1437859_x_at | Eif5a | 0.443 | 0.039 |
| miR-301a | 1426635_at | Acbd3 | 0.443 | 0.039 |
| miR-301a | 1456201_at | 4632427E13Rik | -0.443 | 0.039 |
| miR-301a | 1439931_at | Gsk3b | -0.443 | 0.039 |
| miR-301a | 1424380_at | Vps37b | -0.443 | 0.039 |
| miR-301a | 1451381_at | 1810020D17Rik | 0.443 | 0.039 |
| miR-301a | 1455798_at | Galk2 | 0.442 | 0.039 |
| miR-301a | 1440314_at | NA | -0.442 | 0.039 |
| miR-301a | 1416589_at | Sparc | 0.442 | 0.039 |
| miR-301a | 1437061_at | Mbd1 | -0.442 | 0.039 |
| miR-301a | 1423232_at | Etv4 | -0.442 | 0.039 |
| miR-301a | 1448769_at | Slc35b1 | -0.442 | 0.040 |
| miR-301a | 1455157_a_at | BC039210 | -0.442 | 0.040 |
| miR-301a | 1454642_a_at | Commd3 | 0.442 | 0.040 |
| miR-301a | 1426366_at | Eif2c2 | -0.442 | 0.040 |
| miR-301a | 1429123_at | Rab27a | -0.442 | 0.040 |
| miR-301a | 1424663_at | BC017647 | -0.442 | 0.040 |
| miR-301a | 1424804_at | BC020002 | 0.442 | 0.040 |
| miR-301a | 1447883_x_at | Map1lc3a | -0.441 | 0.040 |
| miR-301a | 1433882_at | Cnot10 | -0.441 | 0.040 |
| miR-301a | 1423531_a_at | Hnrnpa1 | 0.441 | 0.040 |
| miR-301a | 1437102_at | Ythdf1 | 0.441 | 0.040 |
| miR-301a | 1431061_s_at | Peli1 | 0.441 | 0.040 |
| miR-301a | 1415778_at | Morf4l2 | 0.441 | 0.040 |
| miR-301a | 1453721_a_at | Slc31a2 | 0.441 | 0.040 |
| miR-301a | 1429776_a_at | Dnajb6 | 0.440 | 0.040 |
| miR-301a | 1431428_a_at | Nosip | 0.440 | 0.040 |
| miR-301a | 1455013_at | Arih2 | -0.440 | 0.040 |
| miR-301a | 1425975_a_at | Mapk8ip3 | -0.440 | 0.040 |
| miR-301a | 1449095_at | Vps54 | 0.440 | 0.040 |
| miR-301a | 1417890_at | Pdxp | -0.440 | 0.040 |
| miR-301a | 1415952_at | Mark2 | -0.440 | 0.040 |
| miR-301a | 1450978_at | Dvl1 | -0.440 | 0.041 |
| miR-301a | 1416343_a_at | Lamp2 | 0.440 | 0.041 |
| miR-301a | 1426611_at | Psmc2 | 0.439 | 0.041 |
| miR-301a | 1437485_at | Nos1ap | -0.439 | 0.041 |
| miR-301a | 1436858_at | Mbnl2 | 0.439 | 0.041 |
| miR-301a | 1416789_at | Idh3g | 0.439 | 0.041 |
| miR-301a | 1460032_at | NA | -0.439 | 0.041 |
| miR-301a | 1434057_at | Ndufb6 | 0.439 | 0.041 |
| miR-301a | 1433612_at | Ap2s1 | 0.439 | 0.041 |
| miR-301a | 1421082_s_at | Banf1 | 0.439 | 0.041 |
| miR-301a | 1451744_a_at | Zadh1 | 0.438 | 0.041 |
| miR-301a | 1426361_at | Zc3h11a | -0.438 | 0.041 |
| miR-301a | 1422528_a_at | Zfp36l1 | -0.438 | 0.041 |
| miR-301a | 1428380_at | 0610007C21Rik | 0.438 | 0.041 |
| miR-301a | 1434540_a_at | Clta | 0.438 | 0.041 |
| miR-301a | 1428126_a_at | 4921506J03Rik | -0.438 | 0.041 |
| miR-301a | 1438047_at | Zfp384 | -0.438 | 0.042 |
| miR-301a | 1416332_at | Cirbp | 0.438 | 0.042 |
| miR-301a | 1450815_s_at | 2410018M08Rik /// Chchd2 /// LOC100045688 | 0.438 | 0.042 |
| miR-301a | 1425373_a_at | LOC100047604 /// Psmg2 | 0.437 | 0.042 |
| miR-301a | 1459651_s_at | Nup54 | 0.437 | 0.042 |
| miR-301a | 1428140_at | Oxct1 | -0.437 | 0.042 |
| miR-301a | 1418228_at | Nfu1 | 0.437 | 0.042 |
| miR-301a | 1418196_at | Tep1 | -0.437 | 0.042 |
| miR-301a | 1428440_at | Slc25a12 | 0.437 | 0.042 |
| miR-301a | 1423975_s_at | Numa1 | -0.437 | 0.042 |
| miR-301a | 1444258_at | NA | -0.437 | 0.042 |
| miR-301a | 1418000_a_at | Itm2b | 0.437 | 0.042 |
| miR-301a | 1428617_at | Hcfc2 | -0.437 | 0.042 |
| miR-301a | 1460169_a_at | Pctk1 | -0.437 | 0.042 |
| miR-301a | 1429617_at | Cyld | 0.436 | 0.042 |
| miR-301a | AFFX-b-ActinMur/M12481_5_at | Actb | 0.436 | 0.042 |
| miR-301a | 1426061_x_at | NA | -0.436 | 0.042 |
| miR-301a | 1447062_at | NA | -0.436 | 0.042 |
| miR-301a | 1436299_at | Gls | 0.436 | 0.043 |
| miR-301a | 1426004_a_at | Tgm2 | 0.436 | 0.043 |
| miR-301a | 1455261_at | Luc7l | -0.436 | 0.043 |
| miR-301a | 1447926_at | Arl5a | 0.436 | 0.043 |
| miR-301a | 1450102_a_at | Amfr /// LOC100046262 | 0.436 | 0.043 |
| miR-301a | 1423937_at | Kctd5 | 0.435 | 0.043 |
| miR-301a | 1455898_x_at | Slc2a3 | -0.435 | 0.043 |
| miR-301a | 1428417_at | 3110050N22Rik | -0.435 | 0.043 |
| miR-301a | 1455070_at | Dcp2 | -0.435 | 0.043 |
| miR-301a | 1454817_at | Utp18 | 0.435 | 0.043 |
| miR-301a | 1431465_s_at | Fyttd1 | 0.435 | 0.043 |
| miR-301a | 1437718_x_at | Fmod | -0.435 | 0.043 |
| miR-301a | 1438063_at | Mphosph9 | -0.435 | 0.043 |
| miR-301a | 1420948_s_at | Atrx | 0.434 | 0.043 |
| miR-301a | 1452101_at | Blmh | 0.434 | 0.043 |
| miR-301a | 1418570_at | Ncstn | 0.434 | 0.043 |
| miR-301a | 1450153_at | Gopc | 0.434 | 0.043 |
| miR-301a | 1450638_at | Pdcd5 | 0.434 | 0.044 |
| miR-301a | 1450727_a_at | Poldip2 | 0.434 | 0.044 |
| miR-301a | 1454683_at | Sfrs8 | -0.434 | 0.044 |
| miR-301a | 1453841_at | 2310050P20Rik | -0.434 | 0.044 |
| miR-301a | 1423261_at | 1500015O10Rik | -0.433 | 0.044 |
| miR-301a | 1437398_a_at | Aldh9a1 | 0.433 | 0.044 |
| miR-301a | 1416593_at | Glrx | 0.433 | 0.044 |
| miR-301a | 1434004_at | Dhps | 0.433 | 0.044 |
| miR-301a | 1426620_at | Chst10 | 0.433 | 0.044 |
| miR-301a | 1423849_a_at | Clk3 | -0.433 | 0.044 |
| miR-301a | 1416512_at | Nubp2 | 0.433 | 0.044 |
| miR-301a | 1455600_at | Rps3 | 0.433 | 0.044 |
| miR-301a | 1434293_at | Hectd3 | -0.433 | 0.044 |
| miR-301a | 1424390_at | Nupl1 | 0.433 | 0.044 |
| miR-301a | 1422845_at | Canx | 0.433 | 0.044 |
| miR-301a | 1456544_at | Tmem38b | 0.433 | 0.044 |
| miR-301a | 1428228_at | Pgm3 | -0.433 | 0.044 |
| miR-301a | 1452430_s_at | LOC100048559 /// Sfrs1 | 0.433 | 0.044 |
| miR-301a | 1442216_at | NA | -0.433 | 0.044 |
| miR-301a | 1430123_a_at | Akr1a4 | 0.432 | 0.044 |
| miR-301a | 1416994_at | Ttc1 | 0.432 | 0.044 |
| miR-301a | 1436026_at | Zfp703 | -0.432 | 0.045 |
| miR-301a | 1448489_at | Pafah2 | -0.432 | 0.045 |
| miR-301a | 1429534_a_at | Immt | 0.432 | 0.045 |
| miR-301a | 1456131_x_at | Dag1 | -0.432 | 0.045 |
| miR-301a | 1423446_at | Dapk3 | 0.432 | 0.045 |
| miR-301a | 1452545_a_at | Itgb1 | 0.432 | 0.045 |
| miR-301a | 1422539_at | Extl2 | 0.432 | 0.045 |
| miR-301a | 1423532_at | Rnf44 | -0.432 | 0.045 |
| miR-301a | 1455850_at | 2310003H01Rik | -0.432 | 0.045 |
| miR-301a | 1429218_at | Det1 | 0.432 | 0.045 |
| miR-301a | 1450008_a_at | Ctnnb1 | 0.432 | 0.045 |
| miR-301a | 1440998_at | 2310021P13Rik | -0.432 | 0.045 |
| miR-301a | 1435812_at | Unc50 | -0.432 | 0.045 |
| miR-301a | 1453806_at | Ndufb2 | 0.432 | 0.045 |
| miR-301a | 1417451_a_at | Ppia | 0.431 | 0.045 |
| miR-301a | 1438062_at | 4832420A03Rik /// Rsf1 | 0.431 | 0.045 |
| miR-301a | 1417937_at | Dact1 | 0.431 | 0.045 |
| miR-301a | 1438764_at | Anxa7 | -0.431 | 0.045 |
| miR-301a | 1422553_at | Pten | 0.431 | 0.045 |
| miR-301a | 1451959_a_at | Vegfa | 0.431 | 0.045 |
| miR-301a | 1446814_at | NA | 0.431 | 0.045 |
| miR-301a | 1418275_a_at | Elf2 | 0.430 | 0.046 |
| miR-301a | 1422414_a_at | Calm1 /// Calm2 /// Calm3 | 0.430 | 0.046 |
| miR-301a | 1443069_at | NA | -0.430 | 0.046 |
| miR-301a | 1436019_a_at | Trappc1 | 0.430 | 0.046 |
| miR-301a | 1418295_s_at | Dgat1 | -0.430 | 0.046 |
| miR-301a | 1422572_at | Rhog | 0.430 | 0.046 |
| miR-301a | 1428753_a_at | Dgcr6 | 0.430 | 0.046 |
| miR-301a | 1446688_at | NA | -0.430 | 0.046 |
| miR-301a | 1437224_at | Rtn4 | -0.429 | 0.046 |
| miR-301a | 1448175_at | Ehd1 | 0.429 | 0.046 |
| miR-301a | 1416278_a_at | Atp5o /// LOC100047429 | 0.429 | 0.046 |
| miR-301a | 1423548_s_at | Ergic3 | 0.429 | 0.046 |
| miR-301a | 1444660_at | 4932438A13Rik | -0.429 | 0.046 |
| miR-301a | 1440151_s_at | Edf1 | 0.429 | 0.046 |
| miR-301a | 1425678_a_at | Snrk | -0.429 | 0.046 |
| miR-301a | 1424492_at | Trpc2 | -0.429 | 0.046 |
| miR-301a | 1459838_s_at | Btbd11 | 0.429 | 0.046 |
| miR-301a | 1420867_at | LOC100042343 /// Tmed2 | 0.429 | 0.047 |
| miR-301a | 1421968_a_at | Nipa2 | 0.429 | 0.047 |
| miR-301a | 1448357_at | EG666609 /// Snrpg | 0.428 | 0.047 |
| miR-301a | 1433661_at | Nlrx1 | -0.428 | 0.047 |
| miR-301a | 1438264_a_at | Tpp2 | 0.428 | 0.047 |
| miR-301a | 1419278_at | Usp48 | -0.428 | 0.047 |
| miR-301a | 1443603_at | NA | -0.428 | 0.047 |
| miR-301a | 1428390_at | Wdr43 | 0.428 | 0.047 |
| miR-301a | 1425937_a_at | Hexim1 | -0.428 | 0.047 |
| miR-301a | 1416269_at | Atp5j2 | 0.428 | 0.047 |
| miR-301a | 1449857_at | 1200011I18Rik | 0.427 | 0.047 |
| miR-301a | 1422320_x_at | Phxr5 | -0.427 | 0.047 |
| miR-301a | 1420629_a_at | Dnaja3 | 0.427 | 0.047 |
| miR-301a | 1433774_x_at | Cog1 | -0.427 | 0.047 |
| miR-301a | 1431645_a_at | Gdi2 | 0.427 | 0.047 |
| miR-301a | 1422971_at | Gcn5l2 | 0.427 | 0.047 |
| miR-301a | 1420619_a_at | Aes | 0.427 | 0.047 |
| miR-301a | 1442399_at | NA | -0.427 | 0.048 |
| miR-301a | 1448272_at | Btg2 | -0.427 | 0.048 |
| miR-301a | 1423079_a_at | LOC100043869 /// LOC100044630 /// LOC546321 /// Tomm20 | 0.427 | 0.048 |
| miR-301a | 1431228_s_at | 4930526I15Rik | -0.426 | 0.048 |
| miR-301a | 1428766_at | Rnmtl1 | 0.426 | 0.048 |
| miR-301a | 1417961_a_at | Trim30 | 0.426 | 0.048 |
| miR-301a | 1416583_at | Bad | 0.426 | 0.048 |
| miR-301a | 1430634_a_at | Pfkp | 0.426 | 0.048 |
| miR-301a | 1422796_at | Prep | 0.426 | 0.048 |
| miR-301a | 1421895_at | Eif2s3x /// LOC100048746 | 0.426 | 0.048 |
| miR-301a | 1423382_a_at | Hnrpf /// LOC637008 | 0.426 | 0.048 |
| miR-301a | 1453915_a_at | Slc37a3 | 0.426 | 0.048 |
| miR-301a | 1425455_a_at | Churc1 | 0.426 | 0.048 |
| miR-301a | 1434690_at | Lycat | 0.426 | 0.048 |
| miR-301a | 1444056_at | NA | -0.425 | 0.048 |
| miR-301a | 1417248_at | Ralbp1 | -0.425 | 0.048 |
| miR-301a | 1432007_s_at | Ap2a2 | -0.425 | 0.049 |
| miR-301a | 1418988_at | Pex7 | 0.425 | 0.049 |
| miR-301a | 1431062_a_at | Exoc4 | 0.425 | 0.049 |
| miR-301a | 1416459_at | Arf2 | 0.425 | 0.049 |
| miR-301a | 1432207_a_at | Toe1 | 0.425 | 0.049 |
| miR-301a | 1460675_at | Igsf8 | -0.425 | 0.049 |
| miR-301a | 1427702_at | Zfp1 | -0.425 | 0.049 |
| miR-301a | 1455131_at | Opa3 | -0.424 | 0.049 |
| miR-301a | 1423858_a_at | Hmgcs2 | 0.424 | 0.049 |
| miR-301a | 1427425_at | 9130208E07Rik | -0.424 | 0.049 |
| miR-301a | 1441181_at | NA | -0.424 | 0.049 |
| miR-301a | 1417922_at | Kbtbd4 | 0.424 | 0.049 |
| miR-301a | 1426313_at | Bre | 0.424 | 0.049 |
| miR-301a | 1450116_at | D3Ertd300e | 0.424 | 0.049 |
| miR-301a | 1417385_at | Npepps | -0.424 | 0.049 |
| miR-301a | 1423091_a_at | Gpm6b | 0.424 | 0.049 |
| miR-301a | 1448940_at | LOC100045519 /// Trim21 | 0.424 | 0.049 |
| miR-301a | 1423589_at | Arpc4 | 0.424 | 0.049 |
| miR-301a | 1450399_at | Psen1 | 0.424 | 0.049 |
| miR-301a | 1426110_a_at | Lpar1 | 0.424 | 0.050 |
| miR-301a | 1452637_a_at | Bola1 | 0.424 | 0.050 |
| miR-301a | 1450890_a_at | Abi1 | 0.423 | 0.050 |
| miR-301a | 1430614_at | 4632415K11Rik | 0.423 | 0.050 |
| miR-301a | 1416381_a_at | Prdx5 | 0.423 | 0.050 |
| miR-301a | 1449295_at | Sap30bp | 0.423 | 0.050 |
| miR-301a | 1423432_at | Phip | -0.423 | 0.050 |
| miR-301a | 1455479_a_at | Ube2d3 | 0.423 | 0.050 |
| miR-301a | 1450506_a_at | Isg20l1 | 0.423 | 0.050 |
