# Supplementary material for: Genetic variation in hippocampal microRNA expression differences in C57BL/6 J X DBA/2 J (BXD) recombinant inbred mouse strains
Source: BMC Genomics. 2012 Sep 13;13:476. doi: 10.1186/1471-2164-13-476 (PMC3496628; doi:10.1186/1471-2164-13-476)
Supplement: Additional file 2 — Table S2. Genes with miRNA target sites under the QTL for miRNA gene expression. [file 1471-2164-13-476-S2.doc]

| **miRNA** | **chromosome** | **gene** | **target sites** | **top hit**  **(minimum p-value)** | |
| --- | --- | --- | --- | --- | --- |
|  |  |  |  | **r** | **p** |
| **miR301a** | chr1 | Ube2w | 1 | .196 | .358 |
| **miR301a** | chr1 | Ccdc115 | 1 | -.132 | .539 |
| **miR301a** | chr1 | Cox5b | 2 | .071 | .740 |
| **miR301a** | chr1 | Wdr75 | 1 | na | na |
| **miR301a** | chr1 | Abi2 | 2 | -.135 | .530 |
| **miR301a** | chr1 | Ndufs1 | 1 | -.088 | .681 |
| **miR301a** | chr1 | Idh1 | 2 | .252 | .234 |
| **miR301a** | chr1 | Cul3 | 5 | .292 | .167 |
| **miR301a** | chr1 | Atg16l1 | 1 | na | na |
| **miR301a** | chr1 | Rnpepl1 | 1 | .026 | .904 |
|  |  |  |  |  |  |
| **miR212** | chr3 | Bxdc1 | 1 | na | na |
| **miR212** | chr3 | Acad9 | 2 | -.446 | **.029*** |
|  |  |  |  |  |  |
| **miR15b** | chr9 | Nlrx1 | 1 | na | na |
| **miR15b** | chr9 | Arcn1 | 1 | -.136 | .525 |
| **miR15b** | chr9 | Bud13 | 1 | na | na |
|  |  |  |  |  |  |
| **miR301a** | chr17 | Smchd1 | 1 | -.040 | .852 |
| **miR301a** | chr17 | Lycat | 1 | .126 | .558 |
| **miR301a** | chr17 | Slc30a6 | 1 | .229 | .282 |
| **miR301a** | chr17 | Prepl | 2 | .086 | .691 |

Supplemental Table 2. Genes with miRNA target sites under the QTL for miRNA gene expression.

There were no predicted miR-301a target sites in genes under the QTL for miR-301a expression on chromosome 11. na= no gene expression available for the gene is question.
