# Supplementary material for: Genetic variation in hippocampal microRNA expression differences in C57BL/6 J X DBA/2 J (BXD) recombinant inbred mouse strains
Source: BMC Genomics. 2012 Sep 13;13:476. doi: 10.1186/1471-2164-13-476 (PMC3496628; doi:10.1186/1471-2164-13-476)
Supplement: Additional file 3 — Table S3. Summary of all significant correlations of miRNA expression and phenotype measures (p-values < 0.05). [file 1471-2164-13-476-S3.doc]

**Supplemental Table 3. Summary of all significant correlations of miRNA expression and phenotype measures (p-values < 0.05)**

| **miRNA** | **Category** | **Trait name** | **r-value** | **p-value** |
| --- | --- | --- | --- | --- |
| *miR-15b* | General Behavior | Acoustic startle - Background startle response to 65 dB white noise | -0.46 | 0.05 |
| *miR-15b* | General Behavior | Total movement frequencies | -0.60 | 0.00 |
| *miR-15b* | Morphine | Novel open field periphery locomotion 30-45 min [cm] | 0.49 | 0.03 |
| *miR-15b* | General Behavior | Shirpa 29 -- | 0.42 | 0.03 |
| *miR-31* | Cocaine | Novel open field center locomotion 30-45 min [count beam breaks] | 0.46 | 0.04 |
| *miR-31* | Cocaine | Novel open field center locomotion 30-45 min [cm] | 0.47 | 0.04 |
| *miR-31* | Cocaine | Novel open field periphery rears 45-60 min [count beam breaks] | 0.46 | 0.04 |
| *miR-31* | Cocaine | Cocaine Sensitization 10 mg/kg cocaine– Sensitization total open field locomotion minus Cocaine total locomotion (cm in 1 hr) | 0.62 | 0.00 |
| *miR-31* | Cocaine | Open field - locomotion 0-15 min [count beam breaks] | 0.51 | 0.02 |
| *miR-31* | Cocaine | Open field - locomotion 15-30 min post saline[count beam breaks] | 0.45 | 0.05 |
| *miR-31* | Cocaine | Open field locomotion 0-15 min post saline 10 mg/kg [cm] | 0.47 | 0.04 |
| *miR-31* | Ethanol | Blood ethanol concentration following 2.25 mg/kg ethanol [mg/dl] | 0.71 | 0.00 |
| *miR-31* | General Behavior | Light-dark box - total distance traveled in dark compartment [cm] | 0.49 | 0.03 |
| *miR-31* | General Behavior | Light-dark box activity - total distance traveled in both light and dark compartments [cm] | 0.51 | 0.03 |
| *miR-31* | General Behavior | Light-dark box activity - Number of transitions between light and dark [n transitions] | 0.51 | 0.03 |
| *miR-31* | General Behavior | Activity in a novel environment, baseline activity in fear conditioning apparatus [cm travelled] | 0.63 | 0.00 |
| *miR-31* | General Behavior | Fear response - Activity during 1st tone shock pairing [s] | 0.52 | 0.02 |
| *miR-31* | General Behavior | Conditioned Fear Response - Activity in 30 s interval post 3rd tone shock pairing [s] | 0.51 | 0.02 |
| *miR-31* | General Behavior | Open field anxiety - Percentage center time [%] | 0.68 | 0.00 |
| *miR-31* | General Behavior | Open field - total distance traveled 0-5 min [cm] | 0.56 | 0.01 |
| *miR-31* | General Behavior | Open field - total distance traveled 10-15 min [cm] | 0.48 | 0.04 |
| *miR-31* | General Behavior | Open field - total distance traveled 5-10 min [cm] | 0.49 | 0.03 |
| *miR-31* | General Behavior | Open field - total time in perimeter less corner [s] | -0.56 | 0.01 |
| *miR-31* | General Behavior | Open field -total distance in the perimeter [cm] | -0.61 | 0.01 |
| *miR-31* | General Behavior | Open field total distance traveled [cm] | 0.51 | 0.03 |
| *miR-31* | Morphine | Novel open field periphery locomotion 30-45 min [count beam breaks] | 0.52 | 0.02 |
| *miR-34c* | Cocaine | Cocaine induced open field locomotion 0-15 min post 2nd day 10 mg/kg cocaine [count beam breaks] | 0.50 | 0.02 |
| *miR-34c* | Cocaine | Cocaine induced open field locomotion 15-30 min post 2nd day 10 mg/kg cocaine [count beam breaks] | 0.53 | 0.02 |
| *miR-34c* | Cocaine | Cocaine induced open field locomotion 0-15 min post 2nd 10 mg/kg cocaine [cm] | 0.50 | 0.02 |
| *miR-34c* | Cocaine | Cocaine induced open field locomotion 15-30 min post 2nd 10 mg/kg cocaine [cm] | 0.52 | 0.02 |
| *miR-34c* | Cocaine | Novel open field center rearing 0-15 min [count beam breaks] | 0.45 | 0.05 |
| *miR-34c* | Cocaine | Cocaine Sensitization 10 mg/kg cocaine - total open field locomotion [count beam breaks] | 0.47 | 0.04 |
| *miR-34c* | Cocaine | Cocaine Sensitization 10 mg/kg cocaine - total open field locomotion [cm/1 hr] | 0.46 | 0.04 |
| *miR-34c* | General Behavior | Closed arm duration (EPM) | -0.58 | 0.00 |
| *miR-34c* | General Behavior | Open arm duration (EPM) | 0.43 | 0.03 |
| *miR-34c* | General Behavior | Total distance travelled in center | 0.40 | 0.04 |
| *miR-212* | Cocaine | Cocaine induced open field locomotion (15-30 min post 10 mg/kg cocaine) [count beam breaks] | 0.44 | 0.05 |
| *miR-212* | Cocaine | Cocaine induced open field locomotion 0-15 min post 2nd day 10 mg/kg cocaine [count beam breaks] | 0.52 | 0.02 |
| *miR-212* | Cocaine | Cocaine induced open field locomotion 15-30 min post 2nd day 10 mg/kg cocaine [count beam breaks] | 0.50 | 0.02 |
| *miR-212* | Cocaine | Cocaine induced open field locomotion 30-45 min post 2nd 10 mg/kg cocaine [count beam breaks] | 0.50 | 0.03 |
| *miR-212* | Cocaine | Cocaine induced open field locomotion 45-60 min post 2nd 10 mg/kg cocaine [count beam breaks] | 0.45 | 0.04 |
| *miR-212* | Cocaine | Cocaine induced open field locomotion 0-15 min post 2nd 10 mg/kg cocaine [cm] | 0.50 | 0.02 |
| *miR-212* | Cocaine | Cocaine induced open field locomotion 15-30 min post 2nd 10 mg/kg cocaine [cm] | 0.49 | 0.03 |
| *miR-212* | Cocaine | Cocaine induced open field locomotion 30-45 min post 2nd 10 mg/kg cocaine [cm] | 0.49 | 0.03 |
| *miR-212* | Cocaine | Cocaine Sensitization 10 mg/kg cocaine - total open field locomotion [count beam breaks] | 0.51 | 0.02 |
| *miR-212* | Cocaine | Cocaine Sensitization 10 mg/kg cocaine - total open field locomotion [cm/1 hr] | 0.50 | 0.02 |
| *miR-212* | Cocaine | Cocaine Sensitization 10 mg/kg cocaine– Sensitization total open field locomotion minus Cocaine total locomotion (cm in 1 hr) | 0.50 | 0.03 |
| *miR-212* | Ethanol | Distance traveled 15-20 min after saline [cm] | 0.54 | 0.02 |
| *miR-212* | General Behavior | Light-dark box - total distance traveled in dark compartment [cm] | 0.47 | 0.04 |
| *miR-212* | General Behavior | Activity in a novel environment, the altered context of a fear conditioning apparatus [cm travelled] | 0.51 | 0.03 |
| *miR-212* | General Behavior | Activity in a novel environment, baseline activity in fear conditioning apparatus [cm travelled] | 0.49 | 0.03 |
| *miR-212* | General Behavior | Context-fear conditioning, activity in the shock-paired context [cm travelled] | 0.61 | 0.01 |
| *miR-212* | General Behavior | Conditioned Fear Response - Activity in 30 s interval post 3rd tone shock pairing [s] | 0.51 | 0.03 |
| *miR-212* | General Behavior | Open field - total distance traveled 0-5 min [cm] | 0.47 | 0.04 |
| *miR-212* | General Behavior | Acoustic startle - Background startle response to 65 dB white noise | -0.48 | 0.04 |
| *miR-212* | General Behavior | Frequencies to food area, 0- 10 min | 0.42 | 0.03 |
| *miR-212* | General Behavior | Mean speed, 0-10 mins | 0.48 | 0.01 |
| *miR-212* | General Behavior | Total movement frequencies, 0-10 min (HCA) | -0.54 | 0.00 |
| *miR-212* | General Behavior | Total movement frequencies, 10-60 min (HCA) | -0.53 | 0.01 |
| *miR-301a* | General Behavior | Elevated Plus Maze - time spent in the closed arm | -0.45 | 0.02 |
| *miR-301a* | General Behavior | Cue Conditioning - Activity suppression after 3rd tone/shock pairing [s] | -0.48 | 0.04 |
| *miR-301a* | General Behavior | Open field anxiety - Percentage center time [%] | 0.56 | 0.01 |
| *miR-301a* | General Behavior | Open field - total distance traveled 0-5 min [cm] | 0.47 | 0.04 |
| *miR-301a* | General Behavior | Open field - total time in perimeter less corner [s] | -0.52 | 0.02 |
| *miR-301a* | General Behavior | Zero Maze Anxiety – Percentage time spent in open quadrants [%] | -0.52 | 0.02 |
| *miR-301a* | General Behavior | Frequencies to food area, 0- 10 min | 0.42 | 0.03 |
| *miR-301a* | General Behavior | Latency to enter light chamber | -0.42 | 0.03 |
| *miR-301a* | General Behavior | Puzzle box plug test training | -0.39 | 0.05 |
| *miR-301a* | General Behavior | Puzzle box plug test memory | -0.48 | 0.01 |
